# Field-based and laboratory monitoring tools in aerobic gymnastics: a scoping review of measurement properties, physiological relevance, and change following training or competitive exposure

**DOI:** 10.3389/fphys.2026.1920787

**Published:** 2026-09-08

**Authors:** Lihong Liang, Yudan Peng, Yao Xia

**Affiliations:** 1 Geely University of China, Chengdu, China; 2 Gingko College of Hospitality Management, Chengdu, China

**Keywords:** aerobic gymnastics, athletic performance, exercise test, gymnastics, reproducibility of results

## Abstract

**Objective:**

To map field- and laboratory-based assessment and monitoring methods in aerobic gymnastics and summarize their measurement properties, physiological and biomechanical relevance, formal responsiveness evidence, and observed change following training or competitive exposure.

**Methods:**

This systematic scoping review included studies of aerobic gymnasts reporting eligible monitoring tools and outcomes. PubMed, Scopus, and Web of Science were searched through 15 June 2026. Methodological appraisal used Cochrane RoB 2 for randomized trials, design-specific Joanna Briggs Institute checklists for non-randomized studies, an adapted COSMIN-informed framework for measurement-property evidence, and PROBAST for prediction-model and algorithmic components. Findings were descriptively synthesized, and the evidence map was generated from unique report–cell contributions across predefined assessment or exposure groups and outcome domains.

**Results:**

Fifty eligible reports representing 49 study families were included. Evidence concentrated on strength-power and physical-fitness assessment, physiological/metabolic monitoring, and biomechanical, technical, and landing-load analysis. Balance, flexibility, anthropometry, psychophysiological testing, and digital or wearable systems formed smaller, fragmented domains. Training studies generally reported improvements in aerobic, anaerobic, jump, strength, or functional outcomes, whereas congested training was associated with adverse power and landing-load responses. Several studies reported changes in performance, physiological, functional, or biomechanical outcomes following training, rehabilitation, competitive preparation, or congested exposure. However, no included report provided sufficient evidence to establish formal responsiveness as a measurement property. The SAGAT had the most complete preliminary sport-specific evidence, including test–retest reliability, a minimal detectable change estimate, and convergent associations with anaerobic-performance measures, together with observed change across training phases.

**Conclusions:**

Assessment and monitoring in aerobic gymnastics are multidimensional, but most methods remain incompletely validated, and practical application should be considered provisional and decision-specific.

**Systematic Review Registration:**

https://osf.io, identifier p8m4x.

## 1 Introduction

Aerobic gymnastics is a competitive gymnastics discipline comprising Individual Men, Individual Women, Mixed Pair, Trio, Group, Aerobic Dance, and Aerobic Step under the current Fédération Internationale de Gymnastique framework (Alves et al., 2015; Yang, 2026). Although these events share high-intensity choreographed exercise and sport-specific technical content, they differ in athlete number and sex composition, synchronization and collaboration requirements, spatial organization, and, in Aerobic Step, apparatus-specific movement constraints. The present review was designed to cover aerobic gymnastics as a whole rather than one event category. Nevertheless, applicability was interpreted according to the populations and event contexts actually studied; evidence obtained from a group routine, an individual event, an isolated difficulty element, or a youth female sample was not assumed to transfer directly to other categories, sexes, age divisions, or competitive levels. Monitoring and assessment in this sport are necessarily multidimensional because relevant constructs include performance capacity, physiological and energetic demand, strength and power, balance, flexibility, landing load, technical execution, and adaptation to training (Aleksandraviciene et al., 2015; Kyselovičová and Zemková, 2024; Ma et al., 2024c). Interpreting any result also requires evidence concerning measurement properties, because validity, reliability, measurement error, and responsiveness determine whether an observed value can reasonably represent athlete status or change (Mokkink et al., 2010b, 2010a).

Direct physiological work in aerobic gymnasts has reported the use of graded treadmill testing, oxygen uptake, blood lactate, and energy-system partitioning to characterize competitive group exercise (Aleksandraviciene et al., 2015). The study compared international-level national-team gymnasts with national-level university gymnasts. The international-level group showed a numerically greater relative anaerobic contribution, but the between-group differences in energy-system contribution were not statistically significant; this finding should therefore be interpreted as descriptive rather than evidence of a performance-level effect (Aleksandraviciene et al., 2015). Alves et al. (2015) subsequently developed the Specific Aerobic Gymnastics Anaerobic Test as a discipline-specific field assessment of anaerobic performance in aerobic gymnastics. These findings indicate that aerobic-gymnastics assessment cannot be reduced to a single generic aerobic or strength test when the intended interpretation concerns the combined physiological and technical demands of the routine (Aleksandraviciene et al., 2015; Alves et al., 2015).

Recent training and monitoring studies have used tools such as countermovement jump testing; the Specific Aerobic Gymnastics Anaerobic Test; the 20-m multistage fitness test; Y-Balance testing; repeated-jump testing; isokinetic strength testing; Wingate testing; and landing-force assessment to evaluate aerobic gymnasts (Kyselovičová and Zemková, 2024; Ma et al., 2024a, 2024b, 2024c). A randomized 8-week study reported that both jumping interval training and running high-intensity interval training improved aerobic and anaerobic performance in youth female aerobic gymnasts, with jumping interval training showing additional jumping-performance advantages (Ma et al., 2024a). A subsequent study reported that once-weekly jumping interval training enhanced aerobic, anaerobic, and jumping performance in aerobic gymnastics (Ma et al., 2024b). A 2-year elite-athlete follow-up reported improvements in dynamic balance and lower-limb strength endurance but not significant improvements in lower-limb explosive power or anaerobic performance (Kyselovičová and Zemková, 2024). Congested-training monitoring work evaluated strength levels and landing forces in young female aerobic gymnasts during periods of increased training exposure (Ma et al., 2024c).

Coach observation, judging scores, routine video review, and technical analysis remain central to the evaluation of execution, artistry, synchronization, difficulty, and competitive outcome. These approaches do not, however, directly quantify internal physiological strain, neuromuscular status, mechanical loading, recovery, or whether an observed change exceeds expected biological and measurement variation. Field-based assessments may provide standardized and repeatable information with limited equipment and disruption to training, whereas laboratory methods can address narrower physiological, biomechanical, or criterion-related questions that cannot be resolved through observation alone. Such measurements should complement, rather than replace, coaching and judging information, and their selection should be linked to a clearly defined practical or research decision (Currell and Jeukendrup, 2008; Halson, 2014; Bourdon et al., 2017; Gabbett et al., 2017).

Despite this growth, the available evidence is fragmented across physiological characterization, measurement-development work, single-athlete follow-up, intervention trials, and injury-risk studies (Aleksandraviciene et al., 2015; Alves et al., 2015; Kyselovičová and Zemková, 2024; Ma et al., 2024a; Yang, 2026). A systematic review focused on physical fitness and training factors associated with injury risk in aerobic gymnastics and concluded that standardized prospective surveillance and robust analytic approaches are still needed (Yang, 2026). That review does not substitute for a focused map of field and laboratory monitoring instruments, because injury risk, measurement properties, physiological relevance, and observed change following training are related but analytically different questions. Consequently, it remains uncertain which monitoring tools have discipline-specific evidence for measurement quality, which have a clear physiological or biomechanical interpretation in aerobic gymnastics, and which are responsive enough to track training adaptation or changes in competitive exposure (Aleksandraviciene et al., 2015; Alves et al., 2015; Kyselovičová and Zemková, 2024; Ma et al., 2024c). Consequently, two longitudinal questions require separate consideration. The first is whether an instrument has been formally evaluated for responsiveness, that is, whether it can validly detect change in its intended construct. The second is whether a study merely observed a difference in the instrument’s score after training, competitive preparation, rehabilitation, maturation, or altered loading. Observed change may be practically informative, but it does not establish responsiveness unless expected changes were specified and evaluated against an appropriate comparator, external anchor, or measurement-error estimate.

The rationale for this scoping review is therefore not simply to catalog tests used in aerobic gymnastics. Its purpose is to separate whether a method has been used in the discipline; whether its measurement properties permit defensible interpretation; whether its output has a clear physiological, biomechanical, technical, or training-load meaning; whether formal responsiveness has been evaluated; and whether changes have been observed following training or competitive exposure without constituting formal responsiveness evidence. The review aimed to identify and classify field- and laboratory-based assessment and monitoring methods; chart their reported measurement properties; summarize their physiological, biomechanical, performance, and training-load relevance; distinguish formal responsiveness evidence from intervention-related or longitudinal change; and identify priorities for future validation.

## 2 Methods

This review was conducted as a systematic scoping review because its primary purpose was to identify, classify, and map the available evidence on monitoring tools rather than to estimate a single pooled intervention effect. PRISMA 2020 (Page et al., 2021) was used as the overarching systematic review reporting framework, together with PRISMA-ScR (Tricco et al., 2018), the extension specifically developed for scoping reviews. The conduct of the review was also informed by the updated JBI methodological guidance for scoping reviews (Peters et al., 2020). The review protocol was registered on the Open Science Framework (OSF) platform (registration: osf.io/p8m4x; registered on 12/06/2026).

The protocol was registered on the Open Science Framework on 12 June 2026, before the final database searches and formal screening. The final searches were executed on 15 June 2026. The formal screening phase was conducted from 15 to 17 June 2026. Two reviewers independently and in parallel screened all titles and abstracts between 15 and 16 June. Records retained by either reviewer proceeded to report-level eligibility assessment, and the same two reviewers independently assessed the reports between 16 and 17 June. Thus, the dates represent the full active working periods for each stage rather than only the dates on which the final files were completed. The reviewers assessed the same records independently; the screening set was not divided between them. A third reviewer was available to adjudicate unresolved disagreements.

Data charting commenced immediately after completion of the eligibility assessment and was conducted independently by two reviewers between 17 and 20 June. Methodological appraisal was completed independently by two reviewers between 21 and 22 June, followed by consensus verification. Evidence synthesis, quantitative tabulation, and preparation of the evidence map were undertaken between 22 and 23 June.

The review question was “What field-based and laboratory assessment or monitoring methods have been used in aerobic gymnastics, and what evidence is available regarding their measurement properties, physiological and biomechanical relevance, formal responsiveness, and observed change following training or competitive exposure?”

The review used the Population–Concept–Context framework. The population was defined as aerobic gymnasts or athletes explicitly described as practicing sports aerobics, sport aerobics, or competitive aerobics. The concept encompassed any field-based or laboratory monitoring tool, test, instrument, protocol, sensor-derived variable, or physiological or biomechanical index. The context encompassed training, testing, competition, laboratory assessment, field assessment, rehabilitation, injury-risk monitoring, and performance monitoring in aerobic gymnastics.

No substantive changes were made to the review question, population definition, core eligibility criteria, databases searched, or original search date from the registration to the current process conducted. No formal screening, extraction, appraisal, or synthesis was conducted before registration. However, methodological refinements were introduced after registration. Cochrane RoB 2 was applied to randomized trials and PROBAST to prediction-model or algorithmic components to supplement the originally planned JBI and COSMIN-informed appraisal approach. The evidence-map categories were operationalized after examination of the extracted evidence, and additional fields were introduced for study-family linkage, participant overlap, testing schedules, participant accounting, and quantitative description of the evidence base. Formal responsiveness was also distinguished from observed change following training or exposure. These modifications affected appraisal, classification, synthesis, or reporting but did not change the original eligibility decisions. The original OSF registration was preserved unchanged, and a dated amendment log documenting each modification and its rationale was uploaded to the OSF project on 05/08/2026.

### 2.1 Eligibility criteria

Eligible studies included participants of any age or sex who were engaged in aerobic gymnastics, sports aerobics, sport aerobics, or competitive aerobics, including athletes within formal developmental training pathways. In our context, competitive aerobic gymnastics means the recognized sport discipline and its organized development structure. It does not require that every participant had already competed at the time of assessment. General aerobic exercise, recreational fitness aerobics, dance fitness, and other group-exercise formats were not considered part of the eligible population.

The broad inclusion of age groups, sexes, training stages, event categories, and competitive levels was intentional because the review aimed to map the full distribution of the evidence rather than define a homogeneous reference population. These strata were extracted and considered during interpretation, and findings from one athlete population were not assumed to apply directly to another. Studies involving mixed gymnastics disciplines, mixed sports, or athlete and non-athlete comparison groups were eligible only when aerobic-gymnastics data were separately extractable. Data from artistic gymnastics, rhythmic gymnastics, trampoline gymnastics, acrobatic gymnastics, other sports, or non-athletic controls were not included in aerobic-gymnastics-specific counts or conclusions. Studies were eligible when they reported at least one of the following: (1) measurement-property evidence, including content, construct, or criterion validity; reliability; reproducibility; measurement error; or formal responsiveness; (2) physiological or biomechanical relevance, including associations with oxygen uptake, blood lactate concentration, heart rate, energy-system contribution, landing forces, technical performance, or injury-related variables; or (3) observed change in an assessment outcome following training, detraining, rehabilitation, competitive preparation, maturation, congested training, another intervention or exposure, or longitudinal follow-up.

Each report was assigned to one of four applicability categories: (A) directly confirmed competitive or ranked aerobic-gymnastics population; (B) formal aerobic-gymnastics developmental pathway, without requiring current competition participation; (C) aerobic-gymnastics discipline confirmed but competitive tier not reported; or (D) non-athlete instrument-development evidence explicitly targeting an aerobic-gymnastics population. Reports for which the sport discipline could not be confirmed were to be excluded. Category D evidence was retained separately and was not included in athlete-population denominators or generalized as athlete-based monitoring evidence.

Eligible concepts included field-based and laboratory tools used to monitor or assess physiological capacity, energetic demand, anaerobic performance, aerobic capacity, strength, power, repeated-jump ability, balance, flexibility, motor control, landing forces, biomechanical loading, training load, fatigue, readiness, performance adaptation, or other athlete-monitoring constructs relevant to aerobic gymnastics. Studies were eligible when they reported at least one of the following: measurement properties, including validity, reliability, reproducibility, measurement error, sensitivity, or responsiveness; physiological or biomechanical relevance, including associations with oxygen uptake, blood lactate concentration, heart rate, energy-system contribution, landing forces, technical performance, or injury-related variables; or observed change following training, detraining, congested training periods, intervention exposure, competitive preparation, or longitudinal adaptation.

Eligible study designs included measurement-property studies, observational cross-sectional studies, prospective or retrospective cohort studies, intervention trials, quasi-experimental studies, longitudinal follow-up studies, case series, and case reports containing relevant monitoring data. Systematic and narrative reviews were excluded from the evidence synthesis but were used for citation searching and contextual interpretation. Editorials, commentaries, opinion pieces, non-human studies, purely qualitative studies without monitoring data, and studies lacking sufficient methodological information to identify the population or monitoring tool were excluded.

No publication-date restriction was applied because aerobic gymnastics is a relatively specialized field with a limited evidence base. No language restriction was applied at the search stage.

### 2.2 Information sources and search strategy

PubMed, Scopus, and the Web of Science Core Collection were searched from database inception to 15 June 2026. PubMed was accessed through the National Library of Medicine interface, Scopus through the Elsevier interface, and the Web of Science Core Collection through the Clarivate interface. The reference lists of included studies and relevant reviews were also examined to identify potentially eligible reports that had not been retrieved through the electronic database searches.

The electronic strategy combined terms representing the population and sport discipline (such as aerobic gymnastics, sports aerobics, sport aerobics, competitive aerobics, gymnast, and gymnasts) with terms representing assessment, monitoring, physical and physiological performance, biomechanical loading, measurement properties, and observed change following training. The strategy was developed initially in PubMed and translated to the field codes and syntax of Scopus and the Web of Science Core Collection. The searches used database-specific free-text terms and field codes. PubMed terms were searched in the title and abstract fields, Scopus terms in the title, abstract, and keyword fields, and Web of Science terms in the Topic field. No controlled-vocabulary terms, including MeSH terms, were used. No truncation, proximity operator, or NOT term was applied beyond the phrase searching and Boolean combinations reproduced in **Table 1**.

**Table 1:** Search syntax per each database.

Database and interface	Coverage and search date	Exact search syntax	Limits and filters
PubMed (National Library of Medicine)	Database inception to 15 June 2026	(“gymnastics”[Title/Abstract] OR “gymnast”[Title/Abstract] OR “gymnasts”[Title/Abstract] OR “aerobic gymnastics”[Title/Abstract] OR “aerobic gymnasts”[Title/Abstract] OR “competitive aerobics”[Title/Abstract]) AND (“performance”[Title/Abstract] OR “fitness”[Title/Abstract] OR “physiology”[Title/Abstract] OR “physiological”[Title/Abstract] OR “aerobic”[Title/Abstract] OR “endurance”[Title/Abstract] OR “capacity”[Title/Abstract] OR “oxygen uptake”[Title/Abstract] OR “VO2max”[Title/Abstract] OR “anaerobic”[Title/Abstract] OR “neuromuscular”[Title/Abstract] OR “strength”[Title/Abstract] OR “power”[Title/Abstract] OR “jump”[Title/Abstract] OR “load”[Title/Abstract] OR “force”[Title/Abstract] OR “forces”[Title/Abstract] OR “responses”[Title/Abstract] OR “flexibility”[Title/Abstract] OR “flexible”[Title/Abstract] OR “balance”[Title/Abstract] OR “agility”[Title/Abstract] OR “training”[Title/Abstract] OR “workout”[Title/Abstract] OR “workload”[Title/Abstract] OR “monitoring”[Title/Abstract] OR “monitor”[Title/Abstract] OR “evaluation”[Title/Abstract] OR “evaluating”[Title/Abstract] OR “evaluate”[Title/Abstract] OR “measurement”[Title/Abstract] OR “measurements”[Title/Abstract] OR “test”[Title/Abstract] OR “testing”[Title/Abstract] OR “tests”[Title/Abstract] OR “assessment”[Title/Abstract] OR “assessments”[Title/Abstract] OR “assessing”[Title/Abstract] OR “validation”[Title/Abstract] OR “validity”[Title/Abstract] OR “validate”[Title/Abstract] OR “reliability”[Title/Abstract] OR “reliable”[Title/Abstract] OR “reproducibility”[Title/Abstract] OR “reproducible”[Title/Abstract] OR “responsiveness”[Title/Abstract] OR “sensitivity”[Title/Abstract] OR “sensitive”[Title/Abstract])	No publication-date, language, study-design, or document-type limits; no NOT operator.
Scopus (Elsevier)	Database inception to 15 June 2026	TITLE-ABS-KEY(gymnastics OR gymnast OR gymnasts OR “aerobic gymnastics” OR “aerobic gymnast” OR “aerobic gymnasts” OR “sports aerobics” OR “sport aerobics” OR “competitive aerobics”) AND TITLE-ABS-KEY(performance OR fitness OR physiology OR physiological OR aerobic OR endurance OR capacity OR “oxygen uptake” OR “VO2max” OR anaerobic OR neuromuscular OR strength OR power OR jump OR load OR force OR forces OR responses OR flexibility OR flexible OR balance OR agility OR training OR workout OR workload OR monitoring OR monitor OR evaluation OR evaluating OR evaluate OR measurement OR measurements OR test OR testing OR tests OR assessment OR assessments OR assessing OR validation OR validity OR validate OR reliability OR reliable OR reproducibility OR reproducible OR responsiveness OR sensitivity OR sensitive)	No publication-date, language, study-design, or document-type limits; no NOT operator.
Web of Science Core Collection (Clarivate)	Database inception to 15 June 2026	TS=(gymnastics OR gymnast OR gymnasts OR “aerobic gymnastics” OR “aerobic gymnast” OR “aerobic gymnasts” OR “sports aerobics” OR “sport aerobics” OR “competitive aerobics”) AND TS=(performance OR fitness OR physiology OR physiological OR aerobic OR endurance OR capacity OR “oxygen uptake” OR “VO2max” OR anaerobic OR neuromuscular OR strength OR power OR jump OR load OR force OR forces OR responses OR flexibility OR flexible OR balance OR agility OR training OR workout OR workload OR monitoring OR monitor OR evaluation OR evaluating OR evaluate OR measurement OR measurements OR test OR testing OR tests OR assessment OR assessments OR assessing OR validation OR validity OR validate OR reliability OR reliable OR reproducibility OR reproducible OR responsiveness OR sensitivity OR sensitive)	No publication-date, language, study-design, or document-type limits; no NOT operator.

The complete copy-and-paste search syntax for each database is provided in **Table 1**. No publication-date restriction was applied; therefore, each database was searched from inception to the date of the final search. No language restriction was applied during eligibility assessment. Records were not excluded because of publication language when sufficient bibliographic information and a retrievable full text were available for assessment. However, the electronic search terms were primarily formulated in English, and the selected international databases may provide incomplete coverage of journals and reports published only in regional languages. No study-design or document-type filter was applied. The original strategies were independently reviewed by one researcher with expertise in gymnastics, who was not involved in study selection.

To supplement the electronic database searches, backward citation searching was performed by examining the reference lists of all included reports and relevant systematic or narrative reviews. Potentially eligible references identified through this process were assessed using the same population, concept, context, and study-design criteria as database-derived records. Citation searching was intended to identify studies that might have been missed because of inconsistent terminology, incomplete indexing, or publication in less widely indexed journals. It was used as a supplementary retrieval method and was not considered a substitute for searching additional bibliographic or regional databases.

### 2.3 Record management and selection process

All records were exported with complete bibliographic information and available abstracts into EndNote. Duplicates were removed through automated matching of the DOI, PMID, title, author, publication year, and journal fields, followed by manual verification.

Two reviewers independently screened all titles and abstracts against the prespecified eligibility criteria. Each record was classified as eligible, ineligible, or uncertain without access to the other reviewer’s decision. Records retained by either reviewer, including records classified as uncertain, proceeded to full-text assessment. The same two reviewers subsequently assessed the full texts independently and documented the principal reason for exclusion when a report did not meet the eligibility criteria.

After each screening stage, the independent decisions were compared. Disagreements were resolved by returning to the complete record and applying the prespecified Population–Concept–Context framework and eligibility criteria. The reviewers first discussed the relevant eligibility criterion and the information reported in the source document. When consensus could not be reached, a third reviewer independently examined the report and made the final decision. Agreement was evaluated before consensus separately for title-and-abstract screening and full-text assessment using Cohen’s κ. Agreement between the two reviewers before consensus was κ = 0.87 at title-and-abstract screening and κ = 0.94 at full-text assessment. All remaining disagreements were resolved through discussion, and no full-text decisions required adjudication by the third reviewer.

### 2.4 Data charting and data collection process

A standardized data-charting form was developed *a priori* and piloted independently by two reviewers on an initial subset of included studies. Differences identified during piloting were discussed, and the extraction instructions were clarified before the remaining studies were charted. Two reviewers then independently extracted the study characteristics, assessment protocols, measurement properties, outcome data, and appraisal-relevant information from each included report.

The independently completed forms were compared field by field. Discrepancies were resolved by returning to the source report, including the main text, tables, figures, appendices, and supplementary files where available. Numerical discrepancies were checked against the original unit, assessment time point, analysis population, and reported summary statistic. When consensus could not be reached, a third reviewer adjudicated the disputed entry. No single agreement coefficient was calculated for data charting because the form contained heterogeneous categorical, numerical, and narrative fields; agreement was managed and documented at the individual extraction-field level.

During data charting, the included reports were assessed for potential companion publications and participant overlap. For each report, the authors compared the author group, study site, recruitment setting, recruitment or data-collection period, ethics approval, participant characteristics, sample size, competitive level, intervention or exposure, assessment schedule, and reported outcomes. Shared authorship or institutional affiliation alone was not considered sufficient evidence that reports arose from the same cohort.

Reports were classified as confirmed companion publications, probable companion publications, possible or uncertain overlap, related research-program publications without demonstrated overlap, or distinct studies. Confirmed or probable companion reports required several concordant characteristics, such as the same site, sample size, demographic and qualification profile, recruitment period, intervention context, and participant flow. Possible overlap was assigned when reports originated from the same recruitment program and had closely similar participants or calendar periods, but participant identifiers or explicit cross-referencing were unavailable.

EndNote’s rule-based matching functions were used to identify potential duplicate records based on DOI, PMID, title, authorship, publication year, and journal information. All proposed duplicate removals were manually verified. No machine learning prioritization system or automated tool independently made title-and-abstract screening, full-text eligibility, data extraction, methodological appraisal, or study-family decisions. Final decisions at each stage were made by the reviewers. Microsoft Excel was used for data management, coding, and descriptive tabulation, and a Python script was used to generate the evidence map from the finalized report-level coding matrix. No AI tool was used to make final inclusion, exclusion, or methodological-appraisal decisions.

Two reviewers independently completed the study-family assessment using the full reports. Their classifications were compared after completion of the independent assessment. Disagreements were resolved by re-examining the publications and applying the predefined linkage criteria. When consensus could not be reached, the third reviewer independently assessed the reports and made the final decision. All eligible publications were retained because related reports could provide non-duplicated outcomes. However, companion reports were linked under a common study-family identifier, and the distinction between publication-level and cohort-level evidence was maintained throughout the synthesis.

### 2.5 Data items

Data concerning assessment and monitoring tools included the name of the monitoring tool or test, its classification as field-based or laboratory-based, the equipment used, the assessment protocol, outcome variables, scoring method, timing relative to training or competition, and available feasibility information.

To organize the heterogeneous assessment methods represented in the included literature, a domain-based classification framework was applied during evidence synthesis. Each study was classified according to the principal construct addressed by its objective, assessment protocol, and reported outcomes. The monitoring and assessment domains were physiological, metabolic, and cardiorespiratory; anaerobic and sport-specific performance; neuromuscular, strength, power, and physical fitness; balance, flexibility, coordination, and functional movement; biomechanical, kinematic, technical, and landing-load; anthropometric, morphological, and body composition; psychophysiological, perceptual, and neurocognitive; training load, fatigue, recovery, and readiness; health and injury risk; and multidimensional or composite athlete profiling.

A study was assigned to more than one domain when it assessed substantively distinct constructs rather than several variables representing the same underlying construct. The primary domain was determined from the principal study objective and main outcomes, whereas additional domains were retained when they made a distinct contribution to the study. “Multidimensional or composite athlete profiling” was used when several domains were integrated within a common assessment framework and no single construct was clearly predominant. This classification describes what was measured and was used to organize the evidence presented in **Table 1**; it does not indicate that a method was specifically designed, validated, or implemented for repeated athlete monitoring. The domain classification was introduced as an organizational refinement and did not affect the eligibility or inclusion of studies.

Where reported, measurement-property data included reliability, reproducibility, validity, criterion or construct validation, concurrent associations, measurement error, smallest detectable change, minimal important change, responsiveness, sensitivity to change, limits of agreement, intraclass correlation coefficients, coefficients of variation, standard errors of measurement, correlation coefficients, regression coefficients, and standardized effect estimates.

Population-level data used to evaluate applicability included age and age category, sex distribution, aerobic-gymnastics event category, competitive or qualification level, team or federation status, training history, current versus former-athlete status, and developmental training stage. Information extracted for the study-family assessment comprised study site, recruitment or data-collection period, participant characteristics, sample size, ethics approval or registration identifier, intervention or exposure, assessment schedule, reported outcomes, author overlap, related publications cited by the report, and the final relationship judgment. Information that was not reported was recorded as such and was not inferred solely from author or institutional overlap.

Change-related evidence was coded into four mutually interpretable categories: formal responsiveness evidence; observed change following planned training, rehabilitation, or altered exposure; descriptive longitudinal change or repeated monitoring; and repeated measurement undertaken for reliability, agreement, association, surveillance, or prediction without an expected change in the construct.

Formal responsiveness required an explicitly longitudinal measurement-property purpose, prespecified hypotheses concerning the expected direction or magnitude of change, and comparison with an appropriate external anchor, comparator measure, or other longitudinal validity criterion. Measurement-error information, such as the standard error of measurement or smallest detectable change, was also considered when relevant to the interpretation of change. Statistical significance, percentage change, or a pre–post effect estimate alone was insufficient.

Observed change was recorded when an outcome differed after training, rehabilitation, competition preparation, congested loading, or another exposure, irrespective of whether the change was statistically significant.

### 2.6 Outcomes

The primary outcome of the review was a structured map of the field-based and laboratory monitoring tools used in aerobic gymnastics, classified according to their context of use. Secondary outcomes included a summary of reported measurement properties; a synthesis of physiological and biomechanical relevance; a separate appraisal of formal responsiveness evidence; a description of changes observed following training, rehabilitation, competitive preparation, altered exposure, or longitudinal follow-up; and an evidence-gap map identifying areas in which evidence was absent, sparse, or inconsistent.

### 2.7 Effect measures

The review extracted effect estimates appropriate to each study design, including correlation and regression coefficients, mean differences, standardized mean differences, percentage changes, intraclass correlation coefficients, coefficients of variation, standard errors of measurement, minimal detectable changes, limits of agreement, and p-values.

### 2.8 Methodological quality appraisal

Critical appraisal was conducted independently by two reviewers using the framework appropriate to each study design and methodological purpose. Before formal appraisal, the reviewers piloted the relevant tools on 10 studies representing different designs and discussed the interpretation of each item. The full appraisals were then completed without access to the other reviewer’s judgments. Disagreements were resolved by referring to the tool guidance and the complete study report. Unresolved judgments were adjudicated by a third reviewer.

Randomized parallel-group trials with verifiable random allocation were assessed using the Cochrane Risk of Bias 2 tool for the effect of assignment to intervention. The five domains concerned the randomization process, deviations from intended interventions, missing outcome data, outcome measurement, and selection of the reported result. Signalling-question responses and domain-level judgments were retained, and the tool algorithm informed the overall judgment. Reports that used the term random but did not provide enough information to verify sequence generation were classified conservatively as quasi-experimental for appraisal.

Non-randomized studies were assessed with the design-specific JBI critical appraisal checklist: cohort, quasi-experimental, analytical cross-sectional, case series, or case report. Each item was reported as Yes, No, Unclear, or Not applicable. The JBI judgments were not summed and were not converted into numerical percentages or custom low-, moderate-, or high-concern categories.

Measurement properties were defined using the COSMIN taxonomy. Reliability and measurement error were examined using the design principles of the COSMIN Risk of Bias tool for studies of reliability or measurement error. Because the included instruments were predominantly performance tests, physiological measures, or equipment-derived outputs rather than patient-reported outcome measures, the full PROM-specific COSMIN procedure was not applied. Criterion validity, construct validity, content validity, and responsiveness were evaluated with an explicitly adapted, property-specific framework. The retained, modified, excluded, and added criteria, together with all decision rules, are reported in **Supplementary Table 3A**. Each property was evaluated separately, and no aggregate COSMIN study score was generated.

Prediction-model and algorithmic components were assessed using PROBAST. The appraisal included 20 signaling questions across participants, predictors, outcome, and analysis domains, together with domain-level and overall risk-of-bias judgments and applicability concerns. Digital technology was not treated as a study design: intervention-effect components were assessed with RoB 2 or the relevant JBI checklist, whereas an embedded prediction or classification model was assessed separately with PROBAST. Thus, Luo et al. (2025) contributed two complementary appraisals. Complete study-level item and domain judgments, operational definitions, and rationales are provided in the **Supplementary Material**. No study was excluded on the basis of methodological appraisal.

### 2.9 Synthesis methods

The synthesis was descriptive. Studies were grouped according to monitoring setting as field-based, laboratory-based, or mixed. Within these categories, studies were organized according to the monitoring and assessment domains. Multiple domain assignments were retained when a study evaluated substantively distinct constructs, whereas studies integrating several constructs without a clearly predominant domain were classified as multidimensional or composite athlete profiling. The synthesis then summarized the population studied, assessment protocol, reported measurement properties, physiological or biomechanical interpretation, observed change following training or competitive exposure, and practical feasibility for each method and domain.

Population characteristics were synthesized descriptively alongside the monitoring domains. Findings were interpreted within the age, sex, event category, training stage, and competitive level represented in the source study. No pooled reference values or formal comparisons among youth and adult athletes, female and male athletes, developmental and established competitors, or aerobic-gymnastics event categories were undertaken because the relevant subgroups were small, incompletely reported, and methodologically heterogeneous.

The distribution of monitoring domains was organized in studies with direct evidence of competitive participation, formal ranking, team or federation membership, or a stated regional, national, international, or elite level.

#### 2.9.1 Evidence-map construction

The evidence gap map was constructed from the data collection form in which one evidence row represented one explicitly reported pairing of a specific assessment method, monitoring approach, intervention or exposure, and outcome. The complete dataset contained 156 coded evidence rows. Each row retained the report identifier, cohort identifier, original assessment or exposure category, specific method, original outcome domain, specific outcome, evidence type, and supporting notes.

For mapping, the original methods and exposures were consolidated into eight groups: (A) anthropometric, morphological, and body-composition assessment; (B) physiological, metabolic, and cardiorespiratory monitoring; (C) anaerobic and sport-specific performance testing; (D) strength, power, and physical-fitness assessment; (E) balance, flexibility, coordination, and functional-movement assessment; (F) biomechanical, kinematic, technical, and loading analysis; (G) psychophysiological, neurocognitive, and digital-system assessment; and (H) training-intervention and exposure-monitoring evidence. The final category was retained because some reports evaluated changes in outcomes following a training, rehabilitation, competitive preparation, or loading exposure rather than evaluating a standalone measurement instrument. These rows were coded as observed change after exposure and not as formal responsiveness evidence.

Outcomes were consolidated into six domains: (1) measurement properties; (2) physiological, aerobic, and anaerobic response; (3) strength, power, morphology, and functional preparedness; (4) biomechanical loading, technical performance, and digital outputs; (5) balance, flexibility, coordination, and motor control; and (6) injury, training-load, and psychophysiological readiness.

**Figure 1:**
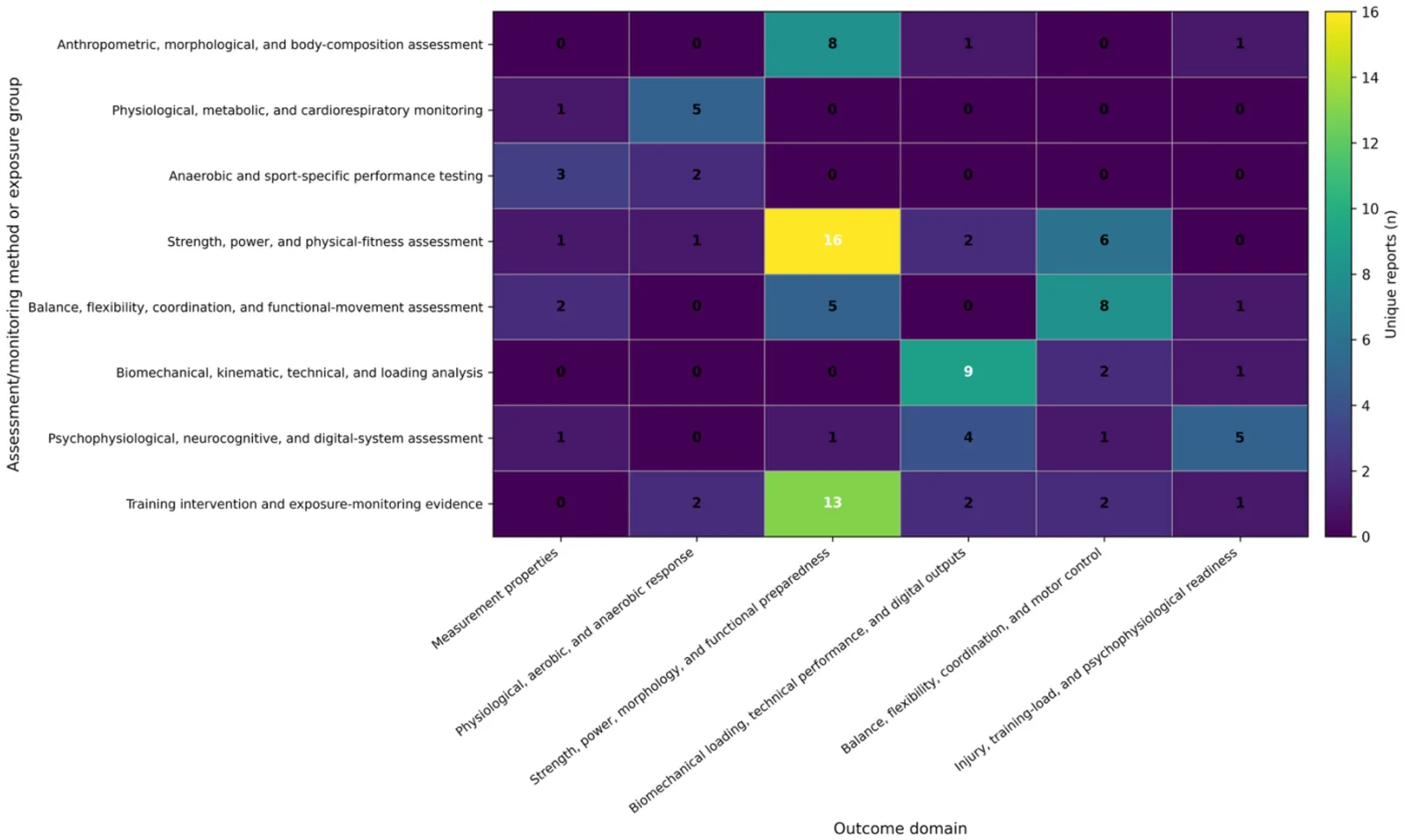
Evidence map by assessment/monitoring method or exposure group and outcome domain. Each cell reports the number of unique reports that contributed at least one coded evidence row to the corresponding method/exposure-group × outcome-domain combination. A report was counted no more than once within an individual cell but could contribute to multiple cells when it evaluated different methods, exposures, or outcome domains. The values do not represent numbers of participants, independent cohorts, comparisons, tools, interventions, or effect estimates. Darker shading indicates a greater number of contributing reports.

The unit displayed in **Figure 1** was the unique report–cell contribution. Each report was counted no more than once within a given method/exposure-group × outcome-domain cell, irrespective of the number of variables, instruments, intervention arms, comparisons, measurement occasions, or evidence rows contributing to that cell. A report could contribute to more than one cell when it examined substantively different assessment or exposure groups or outcome domains. Consequently, the sum of all cells exceeds the number of included reports and should not be interpreted as a count of independent studies, participants, cohorts, tools, interventions, or effect estimates. The coding dataset was maintained in Microsoft Excel and exported in comma-separated-value format. **Figure 1** was generated from the coding matrix using a Python script (Python 3.13.5).

## 3 Results

### 3.1 Study selection

**Figure 2:**
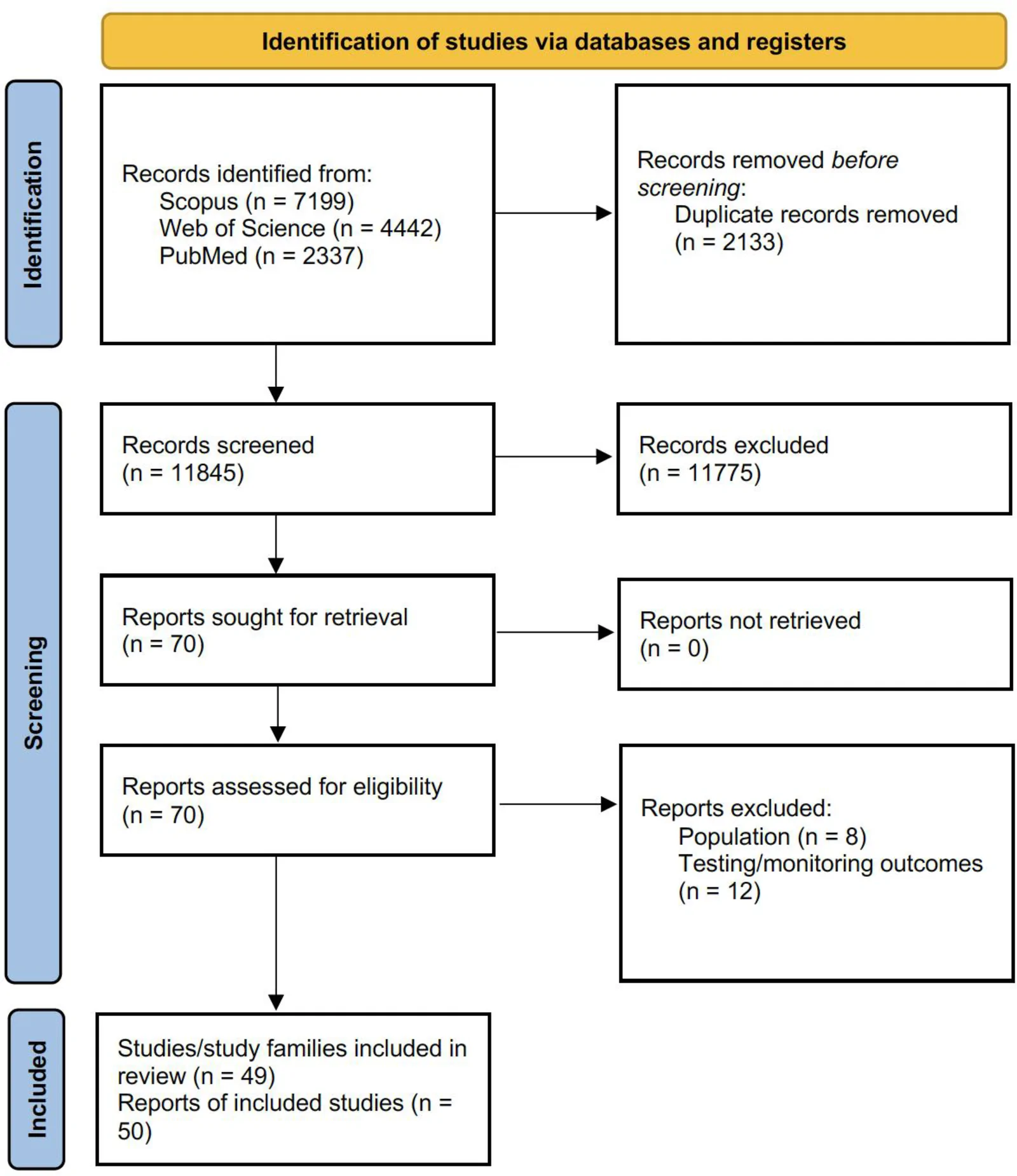
PRISMA 2020 flow diagram.

The searches identified 13,978 records: 7,199 from Scopus, 4,442 from the Web of Science Core Collection, and 2,337 from PubMed. After removing 2,133 duplicate records, 11,845 unique records remained for title and abstract screening. Of these, 11,775 were excluded, and 70 reports were sought and successfully retrieved for full-text eligibility assessment. Twenty reports were excluded at this stage: 12 because they did not include eligible testing or monitoring outcomes and eight because the study population was ineligible. The final review included 50 eligible reports, representing 49 study families (**Figure 2**).

### 3.2 Characteristics of included studies

The characteristics of the included studies are summarized in **Table 2**. The 50 included reports did not necessarily represent 50 independent studies or participant cohorts. The study-family audit identified the two Kokarev et al. (2023a, b) publications as probable companion reports from the same Ukrainian national-team cohort. These reports were retained because they presented different physical- and technical-fitness outcomes, but they were linked as one study family. The 50 reports therefore represented 49 confirmed or probable study families.

**Table 2:** Characteristics of included studies.

Study	Country and setting	Population	Athlete’s level	Design	Exposure duration	Main outcomes	Monitoring/assessment domain(s)
(Abalo-Núñez et al., 2018)	Spain; field or clinical anthropometric testing with season injury follow-up.	51 competitive aerobic gymnasts (45 women, 6 men), mean age 13.61 years, with national, international, and regional competition levels; comparison group of 22 athletes from other sports was also reported.	Competitive	Observational longitudinal	Repeated or longitudinal exposure over one season.	Season injury status; quadriceps angle, bilateral weight-bearing, thigh perimeter; logistic regression prediction models and group comparisons.	Health and injury risk; anthropometric, morphological, and body composition
(Albano et al., 2021)	Italy; laboratory or instrumented testing context using two BTS P-6000 force platforms; exact testing room/site not reported.	Two gold-level Junior B aerobic gymnasts, one male and one female, both aged 16 years.	Competitive	Preliminary pilot case series/methodological tool proposal.	Acute single-session testing after a general warm-up.	Vertical ground reaction force, peak force, rate of force development, symmetry index, contact time, and flight time.	Neuromuscular, strength, power, and physical fitness; biomechanical, kinematic, technical, and landing-load
(Aleksandraviciene et al., 2015)	Lithuania; laboratory treadmill testing and competitive group-routine assessment with portable gas exchange, heart-rate monitoring, and blood lactate.	15 adult female aerobic gymnasts: 6 international-level national-team members and 9 national-level university athletes.	Competitive	Observational comparative physiological study.	Acute testing over two sessions separated by one week; competitive routine averaged 105 s with 10-min recovery monitoring.	Peak oxygen uptake, ventilatory threshold, heart rate, oxygen uptake, lactate, total energy cost, and aerobic/anaerobic energy contribution during routine.	Physiological, metabolic, and cardiorespiratory
(Alves et al., 2015)	Brazil; field-based aerobic-gymnastics testing with laboratory Wingate comparators and simulated competition.	42 high-level female aerobic gymnastics athletes; sub-study samples used sample size = 8, sample size = 10, and sample size = 30 from the same overall recruited sample.	Competitive	Measurement-property study with convergent construct-validity, test–retest reliability, measurement-error, and training-cycle change substudies.	Specific Aerobic Gymnastics Anaerobic Test lasts 80–90 s; reliability trials were 3 days apart; sensitivity was assessed from before preparatory cycle to competition cycle.	Specific Aerobic Gymnastics Anaerobic Test time, Wingate power, plasma lactate response, intraclass correlation coefficient, minimal detectable change, and observed change between preparatory and competition phases.	Anaerobic and sport-specific performance; physiological, metabolic, and cardiorespiratory
(Artemieva et al., 2019)	Ukraine; Children’s Youth Sports School No. 13 in Kharkiv; youth sports school training and testing setting.	32 girls aged 7 to 8 years engaged in sports aerobics/aerobic gymnastics at the initial training stage, allocated to main and control groups of 16 each.	Structured training and developmental stage	Year-long pedagogical quasi-experiment/training-responsiveness study.	Repeated exposure across approximately one year; three sessions per week, 120 min per session; breathing exercises comprised 20% of total session time.	Vital capacity, resting heart rate, Harvard Step Test Index, coordination tests, strength tests, speed tests, speed-strength tests, and flexibility tests.	Neuromuscular, strength, power, and physical fitness; balance, flexibility, coordination, and functional movement; physiological, metabolic, and cardiorespiratory
(Ayziaatullova et al., 2022)	Russia; Lesgaft National State University of Physical Education, Sport and Health, Saint Petersburg.	Sports-aerobics athletes aged 9 to 11 years; L-sit performance; *n* = 28.	Structured training and developmental stage	Cross-sectional anthropometric and technical-performance association study.	Acute single assessment.	L-sit hold time by body-dimension type and correlations between body-segment ratios and L-sit hold time.	Anthropometric, morphological, and body composition; biomechanical, kinematic, technical, and landing-load
(Bota and Urzeală, 2013)	Romania; National University of Physical Education and Sports Club, Bucharest; pre-season training mesocycle before National Championships.	11 high-performance junior female aerobic gymnasts aged 12 to 14 years with eight years of sport experience.	Competitive	Pre–post neuromuscular-control training study with embedded heart-rate routine monitoring.	Heart-rate monitoring during two consecutive routines; simulator training twice weekly for one month, with 2 × 3 series of 10 repetitions at 50%–60% maximum strength capacity.	Heart-rate values by difficulty element and neuromuscular-control scores on a 1-to-10 scale with Six Sigma interpretation.	Physiological, metabolic, and cardiorespiratory; balance, flexibility, coordination, and functional movement
(Bota et al., 2014)	Romania; senior Romanian national aerobic-gymnastics team, with National University of Physical Education and Sport and National Institute for Research in Sport support.	8 senior high-level aerobic gymnasts from Romania’s national team; age and sex distribution not reported.	Competitive	Pilot biomechanical and technical-analysis study using inertial motion capture.	Acute single-session technical monitoring.	Segment rotations, knee projections, center-of-mass, neck and pelvis projections, knee angle over time, shoulder and pelvis rotations, neck-pelvis-to-vertical angle, upper leg angle, and qualitative technical-error causes.	Biomechanical, kinematic, technical, and landing-load
(Budiarti et al., 2022)	Indonesia; Yogyakarta State University; expert/rater-based test-development setting.	Expert/rater panel of 10 raters; instrument targets national development aerobic-gymnastics category aged 9 to 11 years.	Non athlete but used to test instrument	Research-and-development measurement-property study using content validity and interrater reliability.	Single instrument-development assessment; no athlete follow-up.	Aiken’s V for 12 items, Cronbach’s Alpha, single- and average-measures intraclass correlation coefficients, and scoring range from 0 to 8 for 0°–180°.	Balance, flexibility, coordination, and functional movement
(Chayun, 2024)	Russian Federation; Tyumen Oblast sports club psychophysiological testing setting.	38 female Tyumen Oblast aerobic gymnasts aged 9–17 years across 9–11, 12–14, and 15–17 year categories.	Structured training and developmental stage	Cross-sectional psychophysiological monitoring and qualimetric age-category comparison.	Single end-of-season testing session.	Reaction time, correct responses, moving-object reaction balance, and functional mobility of nervous processes.	Psychophysiological, perceptual, and neurocognitive
(Chayun and Sharovarova, 2025)	Russian Federation; University of Tyumen laboratory cardiopulmonary exercise testing setting.	Three female youth aerobic gymnasts aged 12–13 years at first assessment, with more than 7 years of systematic training.	Structured training and developmental stage	Longitudinal observational case series across an annual training cycle.	Repeated testing in February 2022, February 2023, and August 2023.	Maximal oxygen consumption; heart rate and velocity at maximal oxygen consumption; aerobic and anaerobic thresholds.	Physiological, metabolic, and cardiorespiratory
(Chen, 2025)	China; competitive aerobics school-team and youth-training-center testing setting.	56 competitive aerobics athletes: 20 female and 36 male; total age 18.26 ± 1.54 years.	Competitive	Monitoring and prediction-model development/validation study.	Single competitive aerobics individual-event testing dataset with modeling and verification split.	Energy expenditure, metabolic equivalent, oxygen uptake, heart rate, substrate use, accelerometer counts, correlations with metabolic measures, and model prediction error.	Physiological, metabolic, and cardiorespiratory
(D’Anna et al., 2019)	Italy; indoor calibrated video-analysis setting on wooden surface; exact institution not fully reported.	Four senior Italian national-team aerobic gymnasts, two women and two men, aged 18–22 years.	Competitive	Pilot within-subject biomechanical comparison.	Acute single-session technical testing after standardized warm-up.	Jump height, flight time, knee joint crouching angle.	Biomechanical, kinematic, technical, and landing-load
(Ding, 2023)	China; gymnastics club training and field jump-test setting; exact site not reported.	Aerobic gymnastics/aerobics athletes; methods report 20 athletes but figure captions state *N* = 40	Discipline confirmed, but not reported the competitive level	Randomized or quasi-experimental training intervention.	Fourteen-week intervention.	Three-step approach-run first-level right-step jump performance; baseline physical-fitness comparability tests.	Neuromuscular, strength, power, and physical fitness
(Filippova et al., 2006)	Russia; morphofunctional and field/laboratory testing context	Ninety-three female sports-aerobics athletes aged 9–22 years, trained 2–10 years and 12–18 hours per week; compared with 63 schoolgirl/student controls.	Competitive	Observational cross-sectional comparative study.	Cross-sectional assessment after 2–10 years of sports-aerobics exposure; no intervention.	Morphology, physical work capacity, maximum oxygen consumption, cardiovascular efficiency, respiratory indices, strength, power, and flexibility.	Multidimensional or composite athlete profiling
(Ge et al., 2025)	China; Exercise Biomechanics Laboratory of Ningbo University.	30 male competitive aerobics athletes, including 15 high-level and 15 low-level athletes with at least five years of experience.	Competitive	Cross-sectional laboratory biomechanical comparative study.	Acute single-session landing assessment with ten successful trials.	Lower-limb joint kinematics and kinetics, muscle activation and force, coactivation, joint stiffness, energy dissipation, anterior tibial shear force, and patellofemoral joint contact force.	Biomechanical, kinematic, technical, and landing-load
(Guo et al., 2026)	China; controlled computer-based visual-attention testing context.	104 healthy young adults analyzed: 24 table tennis players, 25 action video game players, 28 aerobic gymnastics athletes, and 27 non-trained college students.	Discipline confirmed, but not reported the competitive level	Cross-sectional experience-group comparison using a modified Useful Field of View paradigm.	Acute single-session computer-based testing.	Presentation-duration thresholds for central identification, divided attention, and selective attention at 10° and 16° eccentricity.	Psychophysiological, perceptual, and neurocognitive
(Hao et al., 2022)	China.	30 competitive aerobics athletes without reported history of sports injuries.	Competitive	Eight-week intervention study with experimental and control groups; randomization stated in abstract but methods are incompletely reported.	Repeated eight-week exposure.	Core stability, core-strength test battery, and Group C difficulty-movement completion quality.	Neuromuscular, strength, power, and physical fitness; biomechanical, kinematic, technical, and landing-load
(Kokarev et al., 2023a)	Ukraine; national-team training and field special physical fitness testing	24 qualified Ukrainian national-team aerobic gymnasts aged 18 to 26 years; elite qualification categories reported.	Competitive	Quasi-experimental training intervention with two groups and a cross-experiment phase	Repeated exposure across preparatory/direct pre-competition training; 5 to 6 sessions per week, 2 to 2.5 hours, approximately three months with staged/cross-experiment testing.	Special physical fitness tests: leg swings, flexibility block, straddle L-support, feet lifting to rail, push-ups, standing long jump, and pistol squats.	Neuromuscular, strength, power, and physical fitness; balance, flexibility, coordination, and functional movement
(Kokarev et al., 2023b)	Ukraine; national-team training and aerobic-gymnastics technical testing setting	Highly skilled Ukrainian national-team aerobic gymnasts aged 18 to 26 years; methods report 24 athletes, but technical outcome table reports sample size = 10 per group.	Competitive	Quasi-experimental annual-cycle training intervention after an ascertaining phase	Repeated exposure across annual training cycle; testing at beginning and end of preparatory period and end of competition period.	Technical scores for straddle jump, vertical split, straddle L-support with 360° turn, lateral balance, jump with 360° turn, artistic readiness, and choreographic-compositional readiness.	Biomechanical, kinematic, technical, and landing-load; balance, flexibility, coordination, and functional movement
(Kozina et al., 2017)	Ukraine; Kharkiv national-team/reserve functional, psychophysiological, and vestibular testing context	24 female sports-aerobics athletes who were members of the national team and its reserve in Kharkiv.	Competitive	Cross-sectional multidimensional readiness assessment with factor analysis	Acute single assessment/cross-sectional monitoring.	Factor structure of integral readiness: parasympathicotonia, mobility of nervous system, force, and sense of time.	Multidimensional or composite athlete profiling
(Kyselovičová et al., 2023)	Slovakia; National Sport Centre laboratory.	Seven elite adolescent female aerobic gymnasts were separately extractable from a total sample of 18 elite female gymnasts across aerobic, artistic, and rhythmic disciplines.	Competitive	Cross-sectional comparative and correlational laboratory study.	Acute single-session laboratory testing.	Dynamic balance, isokinetic knee strength/power/work, normalized peak torque, and Y-Balance-isokinetic strength correlations.	Balance, flexibility, coordination, and functional movement; neuromuscular, strength, power, and physical fitness
(Kyselovičová and Zemková, 2024)	Slovakia; National Sports Centre laboratory; elite national-team monitoring context.	One elite adult female aerobic gymnast, aged 22 years at baseline, followed over 24 months between European Aerobics Championships.	Competitive	Longitudinal single-athlete repeated-measures follow-up.	Repeated exposure over two years; T0, T1 after one year, and T2 after two years.	Dynamic balance, postural coordination, vertical jump power, repeated-jump fatigue index, isokinetic strength/power, Wingate performance, lactate, and body composition.	Multidimensional or composite athlete profiling
(Lamošová et al., 2021)	Slovakia; two aerobic gymnastics clubs; field or federation test setting.	Twenty-three girls aged 6 to 11 years involved in aerobic gymnastics; mean baseline age 8.04 years and mean sport age 3.07 years.	Structured training and developmental stage	One-year longitudinal pre–post cohort study without a control group.	Repeated exposure across 1 year; mean training 5.02 hours per week excluding holidays.	Body height, body weight, body mass index, balance, shuttle run, standing long jump, bent arm hang, straddle support, back extension, hanging knee tucks, modified push-ups, sit-ups, and 2-min shuttle endurance.	Multidimensional or composite athlete profiling
(Lamošová et al., 2020)	Slovakia; laboratory-based three-dimensional kinematic analysis using SIMI Motion 3D.	One senior Slovakian national-team female aerobic gymnast, age 21 years, sport age 16 years.	Competitive	Descriptive biomechanical case study.	Acute single-session analysis of two difficulty elements; exact number of trials and total testing duration not reported.	Phase duration, leading-leg velocity and acceleration, center-of-mass height, split angle, standing-leg angle, and technical errors.	Biomechanical, kinematic, technical, and landing-load
Maria Laroëre et al., 2024)	Czech Republic; Prague aerobic gymnastics club and quiet testing room.	Twelve experienced female aerobic gymnasts aged 9 to 22 years; mean age 14.70 years; most with Czech national-level competition experience.	Competitive	Acute within-participant experimental comparison.	Acute single-session exposure with four trials per focus condition.	L-support execution deductions and inter-rater reliability of execution scoring.	Biomechanical, kinematic, technical, and landing-load; Psychophysiological, perceptual, and neurocognitive
(Li, 2025)	China; university competitive-aerobics training centers and AI/IIoT system architecture.	Reported sample includes competitive aerobics athletes aged 18 to 22 years from four training-center groups; denominators are inconsistently described but tables report 100 athletes per group.	Competitive	Technology-development and quasi-experimental evaluation with survey and index-system development.	Repeated/system exposure; exact intervention duration unclear; system training dataset described as 12 weeks.	Body control ability, training difficulty, performance style, athlete satisfaction, and decision-support index architecture.	Multidimensional or composite athlete profiling
(Lopes et al., 2024)	Brazil; Federal University of Lavras high-performance aerobic gymnastics team.	Six adult professional female Brazilian aerobic gymnastics athletes; median age 22.5 years and median height 1.60 m.	Competitive	Repeated-measure body-composition method-comparison study.	Two measurements 30 days apart	Body mass, body mass index, fat mass, fat-free mass, skeletal muscle mass, total body water, body fat percentage, and Bland-Altman agreement.	Anthropometric, morphological, and body composition
(Luo et al., 2025)	China; provincial-level teams and university squads; training system implemented in aerobic gymnastics training with biomechanics laboratory testing.	36 competitive aerobic gymnastics athletes, 18 males and 18 females, mean age 20.8 years, with at least 3 years of competitive experience.	Competitive	Parallel-group randomized controlled trial with embedded algorithmic validation.	Repeated 8-week intervention, 4 sessions per week, 90 min per session.	Algorithmic landing-quality classification, latency, countermovement jump peak ground reaction force, maximum power, rate of force development, drop-jump reactive strength index, and force-output consistency.	Biomechanical, kinematic, technical, and landing-load; neuromuscular, strength, power, and physical fitness
(Ma et al., 2024a)	China; aerobic gymnastics teams with indoor testing and training context; exact team location not reported.	73 youth female regional-level aerobic gymnasts, mean age 16.2 years, 4 to 5 training sessions per week.	Competitive	Randomized controlled trial.	Repeated 8-week intervention; experimental groups completed two supplemental sessions per week before regular training.	Specific Aerobic Gymnastics Anaerobic Test, countermovement jump height, and 20-m multistage fitness test distance.	Anaerobic and sport-specific performance; physiological, metabolic, and cardiorespiratory; neuromuscular, strength, power, and physical fitness
(Ma et al., 2024c)	China; regional aerobic gymnastics club; weekly ForceDecks testing in a quiet room and training-load monitoring in the regular training context.	50 young female regional-level aerobic gymnasts, mean age 16.2 years, trained/developmental classification.	Competitive	Prospective cohort/training-load monitoring study.	Four-week monitoring period; high-frequency group accumulated 2,295 min and standard group accumulated 1,845 min.	Countermovement jump peak power and landing force, squat jump peak power and negative displacement, land-and-hold time to stabilization and peak landing force, and session-rating of perceived exertion training load.	Training load, fatigue, recovery, and readiness; neuromuscular, strength, power, and physical fitness; biomechanical, kinematic, technical, and landing-load
(Ma et al., 2024b)	China; three aerobic gymnastics clubs	69 youth female regional-level aerobic gymnasts	Competitive	Single-blind randomized controlled trial	Repeated exposure over 8 weeks	Specific Aerobic Gymnastics Anaerobic Test completion time, countermovement jump height, and 20-m multistage fitness distance	Anaerobic and sport-specific performance; physiological, metabolic, and cardiorespiratory; neuromuscular, strength, power, and physical fitness
(Mehrtash et al., 2015)	Iran; aerobic gymnastics training context, exact facility not reported	18 boys aged 10–12 years with prior gymnastics experience beginning specific aerobic gymnastics training	Structured training and developmental stage	Single-group repeated-measures training study	Repeated exposure over approximately 24 weeks	Upper-body, trunk, lower-limb, and whole-body motor ability tests	neuromuscular, strength, power, and physical fitness
(Niu, 2022)	China; virtual-reality aerobics simulation/training system	24 aerobics athletes; demographics and competitive level not reported	Discipline confirmed, but not reported the competitive level	Quasi-experimental technology-system evaluation	Repeated exposure over 8 weeks	Technical level scores for presentation, correctness, coherence, coordination, and rhythm	Biomechanical, kinematic, technical, and landing-load
(Mihaela and Dragomir, 2021)	Romania; training-room field/instrumented testing with OptoJump, Polar Team 2, and Lactate Pro 2.	13 junior aerobic gymnasts, 7 female and 6 male athletes, aged 14–16 years.	Competitive	Descriptive methodological testing-protocol study.	Acute single-session testing.	Jump count, contact time, jump height, take-off power, maximum heart rate, and post-effort capillary blood lactate.	Neuromuscular, strength, power, and physical fitness; physiological, metabolic, and cardiorespiratory
(Panfilova, 2025)	Russia; gym/private-room psychophysiological testing context before training.	24 female aerobic gymnastics athletes aged 12–17 years: Age Group 1 sample size = 10 and Age Group 2 sample size = 14.	Structured training and developmental stage	Cross-sectional age-group comparison using computerized psychophysiological testing.	Acute single-session testing in the off-season.	Psychomotor reaction time, precise responses, distraction tolerance, tapping intervals, nervous-system mobility, balance, and intensity.	Psychophysiological, perceptual, and neurocognitive
(Sang, 2023)	Philippines; athletes from an Institute of Physical Education aerobics team	18 aerobics or competitive-aerobics athletes, experimental group sample size = 9 and control group sample size = 9; exact sex distribution not reported.	Discipline confirmed, but not reported the competitive level	Randomized controlled training study with limited reporting.	Repeated 12-week intervention exposure.	Functional Movement Screen total and subtest scores; 10 special physical fitness indicators including push-up, long-jump, flexibility, support, and turn/split tests.	Balance, flexibility, coordination, and functional movement; neuromuscular, strength, power, and physical fitness
(Sun, 2022)	China; college or university aerobics training/testing context.	10 young female competitive aerobics athletes aged 18–20 years, described as second-level athletes with 3–4 years of training; selected after pre-testing 20 players.	Competitive	Small controlled pre–post core-strength training study; reporting is consistent with quasi-experimental controlled intervention.	Repeated exposure; 3–4 sessions per week, 1.5 hours each; total intervention duration not reported.	Core-strength and special physical fitness indicators: two-foot single-arm push-ups, acute angle and leg support, hanging leg lift, split-leg jump, and standing high body forward bending.	Neuromuscular, strength, power, and physical fitness; Balance, flexibility, coordination, and functional movement
(Tibenská and Medeková, 2014)	Slovakia; junior aerobic-gymnastics training/testing context.	12 female junior aerobic-gymnastics competitors, mean baseline age 14.08 ± 1.19 years, range 13–17 years; training five times per week for 120 min.	Competitive	Two-year longitudinal descriptive monitoring and performance-profiling study.	Repeated exposure over two one-year preparation cycles with nine 3-month assessment intervals.	Body height, body weight, body mass index, body fat percentage, active body weight, sit-ups, pull-ups, modified push-ups, standing long jump, backwards tandem walking, shuttle run, Jacík’s test, and competition ranking/points.	Multidimensional or composite athlete profiling
(Todorova et al., 2021)	Ukraine; Municipal children’s and youth sports school No. 13, Kharkiv.	24 female aerobic gymnasts aged 12–14 years at the specialized basic preparation stage; main group sample size = 12 and control group sample size = 12.	Structured training and developmental stage	One-year controlled pedagogical experiment evaluating an author-designed annual training model.	Repeated exposure across one annual macrocycle; methods report six 150-min sessions per week, while the model table varies weekly training days, sessions, and duration.	Special physical preparedness, technical preparedness, coordination, strength, speed, speed-strength, flexibility, functional capacity, Harvard Step Test index, and competition readiness.	Multidimensional or composite athlete profiling
(Trisanda et al., 2022)	Indonesia; Rumbai gymnastics hall, Pekanbaru.	Eight Riau aerobic gymnastics athletes; sex-specific results imply four male and four female athletes; age and competitive level not reported.	Discipline confirmed, but not reported the competitive level	Single-group pre–post quasi-experimental intervention.	Repeated exposure; 12 meetings.	Standing broad jump leg muscle power.	Neuromuscular, strength, power, and physical fitness
(Viktorovich and Vladimirovna, 2019)	Russia and Poland; Russian national team monitoring during preparation for the 2017 World Games in Omsk, Moscow, and Wroclaw.	18 highly skilled sports-aerobics athletes from the Russian national team: 8 women and 10 men, including one honored master of sports, eight international masters of sports, and nine masters of sports.	Competitive	Prospective longitudinal psychophysiological monitoring study.	Repeated monitoring across three preparation/competition stages.	Complex visual-motor response, nervous-process mobility, response to moving object, static and dynamic tremometry, Zung depression scores, self-assessment, and competition-outcome interpretation.	Psychophysiological, perceptual, and neurocognitive; Training load, fatigue, recovery, and readiness
(Wang, 2015)	China; Hunan University of Science and Engineering affiliation; exact testing site not reported.	Competitive aerobics athletes; sample details not reported; one group and three illustrative athletes discussed.	Discipline confirmed, but not reported the competitive level	Methodological fuzzy comprehensive evaluation/descriptive profiling study.	Acute or cross-sectional assessment/modeling.	Comprehensive body-function score; body shape/function/physical quality components; athlete rankings.	Multidimensional or composite athlete profiling
(Wu, 2023)	China; sports-college setting; corresponding institution in Ganzhou, Jiangxi.	Four female sports-college gymnasts born in 2001.	Discipline confirmed, but not reported the competitive level	Small uncontrolled pre–post intervention study.	Repeated exposure from March to July 2021	Core composite index, handstand/inversion control time, special quality data, 8-point star-test model.	Neuromuscular, strength, power, and physical fitness; Balance, flexibility, coordination, and functional movement
(You, 2023)	China; competitive aerobics training base.	Forty young competitive aerobics athletes aged 16–18 years.	Competitive	Randomized controlled pre-test/post-test intervention.	Repeated exposure: 12 weeks.	Core-strength tests and Group B difficulty-movement execution.	Neuromuscular, strength, power, and physical fitness; Biomechanical, kinematic, technical, and landing-load
(Yu and Liu, 2024)	China; laboratory motion-capture and surface electromyography testing.	Sixteen mixed-sex university aerobic gymnasts grouped as high-level or average.	Competitive	Cross-sectional comparative biomechanical study.	Acute single-session testing; three C715 trials per athlete.	C715 stage duration, joint angles, velocities, and integral electromyography.	Biomechanical, kinematic, technical, and landing-load
(Yudho et al., 2024)	Indonesia; wearable-sensor stability testing setting not specified.	Eight participants: four active and four retired/former aerobic gymnastics athletes.	Discipline confirmed, but not reported the competitive level	Case-study cross-sectional comparison.	Acute single-session exposure; approximately 10 s per task.	Sagittal and frontal plane postural-stability variables and internal correlations.	Balance, flexibility, coordination, and functional movement
(Yusupava et al., 2021)	Ukraine; children’s and youth sports school No. 13, Kharkiv.	Twenty young female aerobic gymnasts at the initial-preparation stage; age reported inconsistently as 7–9 years in the abstract and 7–8 years in the methods.	Structured training and developmental stage	Cross-sectional exploratory factor-analysis profiling study.	Acute or cross-sectional assessment.	Speed-strength, coordination, strength, flexibility, speed, special endurance, anthropometry, heart rate, vital capacity, PWC170, and technical-element tests.	Multidimensional or composite athlete profiling
(Zhang, 2023)	China;	Twenty aerobics/aerobic-gymnastics athletes with ankle joint injuries; sex and competitive level not reported.	Discipline confirmed, but not reported the competitive level	Two-group pre-test/post-test intervention, described as random sampling to experimental and control groups.	Repeated exposure: 8 weeks, 3 sessions per week.	Lumbar/core joint movement amplitudes and injured ankle static balance parameters.	Health and injury risk; Balance, flexibility, coordination, and functional movement
(Zhao et al., 2025)	China; university team and regional professional team testing during regular training sessions.	Forty-two high-level competitive aerobics athletes, 18 male and 24 female, aged 19.3 ± 2.1 years; all at least national second-class ranking.	Competitive	Prospective injury-surveillance and predictive-modeling study.	Six-month in-season injury surveillance plus acute sport-specific sensor testing.	Lower-limb injury incidence, ordinal injury-risk classification, model accuracy, macro-F1, macro-AUC, high-risk recall, Brier score, and posterior biomechanical predictor estimates.	Health and injury risk; Biomechanical, kinematic, technical, and landing-load

Monitoring/assessment domains were assigned according to the construct addressed by the principal study objective and outcomes. Multiple domains are reported when a study assessed substantively distinct constructs. “Multidimensional or composite athlete profiling” was used when several domains were integrated without a clearly dominant construct. Domain classification describes what was measured; it does not indicate that the method was designed or validated for repeated athlete monitoring.

Included studies were randomized trials, quasi-experimental training studies, observational cross-sectional and longitudinal designs, measurement-property studies, case-series or case-study reports, biomechanical analyses, and prediction-model or digital-system studies. Populations were most often youth, junior, national-level, regional-level, or high-level aerobic gymnasts, with several studies restricted to female athletes and several reporting mixed-sex or incompletely described samples. China contributed 18 reports, followed by Ukraine and Russia or the Russian Federation with six each, Slovakia with five, Romania and Indonesia with three each, Italy and Brazil with two each, and Spain, Lithuania, the Czech Republic, Iran, and the Philippines with one each. Twenty reports involved adult-only samples, 18 involved youth-only samples, four included mixed or transition-age samples, and seven did not report age sufficiently for classification. One additional report concerned instrument development using expert raters rather than athletes. Population verification classified 31 reports as directly involving competitive, ranked, national-team, federation-affiliated, or otherwise explicitly competition-level athletes. Nine reports examined athletes within a formal aerobic gymnastics developmental pathway, and nine confirmed aerobic gymnastics as the sport discipline but did not report the athletes’ competitive tier. One additional report (Budiarti et al., 2022) evaluated a flexibility instrument intended for the national-development category using an expert/rater panel rather than an athlete sample.

Eligibility was discipline-wide, but the available evidence was not distributed evenly across event categories or athlete strata. The event category was frequently absent or incompletely reported, and the available studies did not permit formal comparison among individual men, individual women, mixed pair, trio, group, aerobic dance, and aerobic step. The evidence was also concentrated in youth or junior athletes, female samples, and athletes described using heterogeneous terms such as regional, national, international, high-level, or elite. Accordingly, the synthesis retains the competitive-level terminology reported by each source and does not assume transferability across event category, sex, age division, or performance caliber.

**Table 3** presents the monitoring tools, interventions, exposures, and delivery characteristics extracted for each included study. Most tools were classified as field-based or applied-setting assessments, followed by laboratory-based tools or mixed field-based and laboratory assessments. The mapped tools covered anthropometric and alignment measures; field-based physical-fitness batteries; the Specific Aerobic Gymnastics Anaerobic Test; Wingate testing; graded treadmill, oxygen-uptake, heart-rate, and blood-lactate monitoring; force-platform testing; countermovement jump, repeated-jump, isokinetic-strength, flexibility, balance, and functional movement tests; kinematic and biomechanical analyses; session rating of perceived exertion; psychophysiological tests; wearable-sensor models; and digital decision-support systems. Intervention or exposure studies most commonly evaluated special physical training, core-strength or conditioning programs, jumping interval training, high-intensity running interval training, resistance or plyometric interventions, breathing-exercise-supported training, simulator-based or virtual-reality-supported training, or congested training exposure.

**Table 3:** Monitoring-tool, intervention, exposure, and delivery characteristics.

Study	Monitoring tool, test, or intervention	Administration and delivery procedures	Setting	Testing occasions, intervention/exposure duration, and follow-up
(Abalo-Núñez et al., 2018)	Bilateral quadriceps angle goniometry, bilateral weight-bearing using two Jackson platforms, and thigh perimeter measurement. These were standardized as lower-limb anthropometric and biomechanical monitoring tools.	Individual anthropometric testing by the same assessor; measurements on two different days at the same time of day; each characteristic measured three times in both records.	Field or clinical anthropometric testing; exact site not reported.	Two measurement days; injury status followed over one competitive season (exact season length not reported).
(Albano et al., 2021)	Push Up Jump Test, Explosive Push Up Test, and Free Fall Test using two BTS P-6000 force platforms to evaluate upper-body force expression, symmetry, and landing management.	Athletes performed tests after a general warm-up. Force data were collected at 1000 Hz and processed with SmartAnalyzer software using a second-order Butterworth low-pass filter at 580 Hz.	Laboratory or instrumented testing context.	One testing session after a general warm-up; number of trials and total session duration not reported.
(Aleksandraviciene et al., 2015)	Maximal treadmill testing plus competitive routine monitoring with portable gas exchange, heart-rate monitoring, blood lactate, and energy-system calculations.	Treadmill test conducted individually in laboratory; group routine performed after standardized warm-up and rest with portable instrumentation.	Mixed laboratory and competitive-routine assessment.	Two testing sessions separated by one week: incremental treadmill testing and competitive-routine assessment; the routine averaged 105 s and recovery monitoring continued for 10 min.
(Alves et al., 2015)	Specific Aerobic Gymnast Anaerobic Test, a field-based repeated-sprint test using sport-specific tuck jumps, push-ups, L-supports, floor touches, and directional changes.	Individual athlete completes two six-bout sets as fast as possible; experienced coach must verify technical execution; verbal encouragement reported.	Field-based aerobic gymnastics training/testing area with laboratory and simulated-competition comparators.	SAGAT duration 80–90 s; test–retest assessments separated by 3 days; additional assessments conducted before the preparatory cycle and during the competition phase.
(Artemieva et al., 2019)	Author-designed special physical training methodology plus a field-test battery for special physical preparedness in young aerobic gymnasts.	Group-based youth sports school training; breathing exercises inserted in warm-up, main part, and after training.	Children’s Youth Sports School No. 13, Kharkiv.	One-year intervention; three 120-min sessions per week; breathing exercises comprised approximately 20% of session time.
(Ayziaatullova et al., 2022)	Anthropometric body-segment assessment and L-sit static-strength element hold-time testing.	Individual body-segment measurement and technical-performance assessment; protocol details limited.	Sports-aerobics assessment setting; exact facility not reported.	One cross-sectional assessment occasion; no longitudinal follow-up.
(Bota and Urzeală, 2013)	Polar heart-rate monitoring during routines and Ergosim simulator-based neuromuscular-control training/testing.	Heart-rate monitors attached during routines; simulator provided individualized visual feedback against an ideal movement model.	National University of Physical Education and Sports Club, Bucharest.	Heart rate monitored during two consecutive routines; simulator training delivered twice weekly for 1 month (2 x 3 series of 10 repetitions at 50-60% maximum strength capacity).
(Bota et al., 2014)	Xsens MVN BIOMECH inertial motion capture for technical-error analysis of group C jumps.	Whole-body inertial capture using 17 MEMS-IMU units and real-time MVN Studio visualization for coaches and athletes.	Romanian senior national-team training/testing context with National Institute for Research in Sport equipment.	One technical-analysis session; number of element trials and total session duration not reported.
(Budiarti et al., 2022)	Aerobic-gymnastics-specific flexibility test construction for national development category using a degree-scale image and best of two trials.	Intended field administration by coaches or teachers; evidence in this report comes from expert/rater assessment, not athlete testing.	Expert/rater instrument-development setting, Indonesia.	Single expert-rating/instrument-development phase; two test attempts; no athlete testing or athlete follow-up.
(Chayun, 2024)	UPFT 1/30 Psychophysiologist psychomotor-test battery including complex visual-motor reaction, reaction to a moving object, and functional mobility of nervous processes.	Individual device-based psychophysiological testing of female aerobic gymnasts across age categories at the end of the season.	Sports club psychophysiological testing setting.	One end-of-season testing session.
(Chayun and Sharovarova, 2025)	Incremental treadmill cardiopulmonary exercise testing with Quark CPET to assess maximal oxygen consumption and aerobic and anaerobic thresholds.	Individual laboratory testing repeated across three timepoints in a small youth-athlete case series.	University laboratory cardiopulmonary testing setting.	Three testing occasions: February 2022, February 2023, and August 2023.
(Chen, 2025)	Triaxial accelerometer, portable gas metabolism analyzer, heart-rate monitoring, and machine learning/regression modeling for competitive-aerobics energy-expenditure prediction.	Individual competitive aerobics event testing; data split into modeling and verification groups.	Competitive aerobics testing setting.	One sport-specific testing dataset, divided into model-development and verification samples; routine duration not fully reported.
(D’Anna et al., 2019)	Video-based kinematic analysis of two equal-feet pre-jump techniques for Group C jumps: with flight phase before crouching and without flight phase before crouching.	Individual technical testing after approximately 15-min standardized warm-up; HERO4 Black camera at 90 frames per second and Kinovea software; anatomical markers and space calibration used.	Instrumented video-analysis testing context.	One testing session after a standardized warm-up; two valid trials per test, with six jumps per technique included in analysis.
(Ding, 2023)	Elastic-band lower-limb strength training intervention intended to improve jump performance in aerobic gymnastics athletes.	Experimental group received elastic-band training; control group received routine training; exact supervision, exercises, and resistance progression not reported.	Training and field jump-test setting.	Pre- and post-intervention assessment over 14 weeks; session frequency, duration, resistance dose, and progression not reported.
(Filippova et al., 2006)	Morphofunctional, cardiorespiratory, respiratory, and sport-relevant physical-fitness monitoring battery.	Individual testing of female athletes and controls using anthropometry, somatotype assessment, step-test indices, heart-rate and blood-pressure measures, respiratory indices, and fitness tests.	Morphofunctional, cardiorespiratory, respiratory, and physical-fitness testing context.	One cross-sectional assessment after 2–10 years of sports-aerobics participation; no repeated within-athlete testing or follow-up.
(Ge et al., 2025)	Instrumented rotational-landing assessment using Vicon motion capture, AMTI force plates, surface electromyography, and OpenSim-derived musculoskeletal variables.	Individual laboratory testing after standardized warm-up and familiarization; participants performed 360° straight-body jumps and landed with both feet on force plates.	Exercise Biomechanics Laboratory of Ningbo University.	One landing-assessment session with 10 successful trials; at least 30 s rest between trials.
(Guo et al., 2026)	Modified Useful Field of View visual-attention task with central identification, divided attention, and selective attention subtests.	Individual computer-based testing using adaptive presentation-duration thresholds, keyboard and mouse responses, and chin-rest-stabilized viewing.	Controlled laboratory or computer-based testing setting in China.	One computer-based testing session; short practice before each subtest; no follow-up.
(Hao et al., 2022)	Systematic core-strength training added to traditional strength and Group C difficulty-movement training; outcomes included eight-point star offset, core-strength tests, and judged Group C difficulty movements.	Training was delivered over eight weeks; experimental group received added core training and control group traditional training; details of supervision and exercise list are sparse.	College or training setting; exact institution/site not reported.	Pre- and post-intervention assessments over 8 weeks; session frequency, duration, and adherence not reported.
(Kokarev et al., 2023a)	Innovative physical/functional training used TRX, Tabata, M.A.X., HIIT, Functional Step, and 6-D Sliding; outcomes measured by special physical fitness test battery.	Supervised national-team group training; comparison with traditional program; cross-experiment design.	National-team training and field physical-preparedness testing context.	Assessments across preparatory and direct pre-competition training; 5–6 sessions/week, 2-2.5 h/session, over approximately 3 months, with an additional cross-experiment phase.
(Kokarev et al., 2023b)	Innovative technical training used 6-D Sliding, Procedos, EXOS, and light platform; outcomes measured with technical control exercises and readiness scores.	Supervised national-team technical training; experimental program compared with classical training program.	National-team training and technical testing setting.	Assessments at the beginning and end of the preparatory period and at the end of the competition period within one annual training cycle.
(Kozina et al., 2017)	Multidimensional readiness battery combining autonomic, psychophysiological, physical, treadmill, and vestibular assessments with factor analysis.	Individual testing and subsequent statistical modeling; no training intervention.	Sports-aerobics functional, psychophysiological, and vestibular testing setting.	One multidimensional assessment occasion; total duration and test order not fully reported.
(Kyselovičová et al., 2023)	Y-Balance testing and Biodex isokinetic knee flexion/extension testing; body-composition assessment used for profiling and torque-context interpretation.	Individual laboratory testing with standardized warm-up, Y-Balance testing, rest, and Biodex dynamometry.	National Sport Centre laboratory, Slovakia.	One laboratory visit; Y-Balance best of three trials per direction and Biodex testing at 60, 180, and 300 degrees/s.
(Kyselovičová and Zemková, 2024)	Comprehensive high-performance monitoring battery: InBody, IMOOVE, Y-Balance, OptoJump vertical jumps, 60-second repeated jumps, Biodex isokinetic testing, and Wingate testing.	One elite athlete tested at the same laboratory and time of day across three annual timepoints; normal coach-designed training continued.	National Sports Centre laboratory, Slovakia.	Three assessment occasions: baseline, 12 months, and 24 months; total follow-up 2 years.
(Lamošová et al., 2020)	SIMI Motion 3D analysis of two Group D balance and flexibility difficulty elements: Free illusion to vertical split and Free illusion to free vertical split.	Single national-team athlete assessed with eight synchronized high-speed infrared cameras and 20 passive markers.	Laboratory-based three-dimensional kinematic analysis.	One assessment session involving two difficulty elements; number of trials and total session duration not reported.
(Lamošová et al., 2021)	Anthropometric measures and a 10-test Slovak Gymnastics Federation motor test battery for young aerobic gymnasts.	Field or club-based pre–post testing before and after one year of regular aerobic gymnastics training and competitions.	Two aerobic gymnastics clubs in Slovakia.	Pre- and post-assessment over 1 year of regular training (mean 5.02 h/week, excluding holidays).
(Maria Laroëre et al., 2024)	L-support static strength skill performed under external focus, internal focus, and no-focus conditions; execution judged using FIG-based criteria.	Individual repeated trials in a counterbalanced within-participant design; standardized instructions and rest intervals.	Quiet testing room in Prague club context.	One experimental session; four 4-s L-support trials per condition, 20 s between trials, and 3 min between conditions.
(Li, 2025)	AI/IIoT training decision system integrating wearable sensors, edge computing, cloud analytics, CNN-LSTM action recognition, and reinforcement-learning recommendations.	Web/mobile decision-support interface for coaches and athletes; traditional system comparator not clearly described.	University competitive-aerobics training centers and digital infrastructure.	System-development dataset described as 14,300 motion segments from 400 athletes over 12 weeks; actual implementation and follow-up duration unclear.
(Lopes et al., 2024)	Body-composition monitoring using skinfolds, ultrasound, and octapolar bioimpedance.	Individual morning assessments with standardized restrictions; same athletes measured at two timepoints.	Federal University of Lavras high-performance aerobic gymnastics team.	Two assessment occasions 30 days apart; no intervention.
(Luo et al., 2025)	Wearable-sensor intelligent training system using inertial measurement units, force plate, gradient-boosting landing-quality classifier, force-quality score, and adaptive progression rules; compared against three conventional/progressive/conditioning training approaches.	Group training with individualized real-time tablet feedback; coaches supervised and retained higher-level technical decisions; assessors blinded for testing/data processing.	Training environment with sensor/force-plate feedback plus biomechanics laboratory outcome testing.	Pre- and post-intervention assessments over 8 weeks; 4 sessions/week, 90 min/session.
(Ma et al., 2024a)	Jumping interval training and running high-intensity interval training added to regular aerobic gymnastics training; JIT used repeated maximal bilateral countermovement jumps, while HIIT used running intervals at 105% to 110% maximal aerobic speed.	Researcher-supervised supplemental sessions before regular training; usual control continued standard aerobic gymnastics training.	Indoor training and testing context; exact clubs/sites not reported.	Pre- and post-intervention assessments over 8 weeks; two supplemental sessions/week before regular training; 4–5 sets of 30–40 s with 30-s recovery.
(Ma et al., 2024c)	Jumping interval training with maximal continuous bilateral countermovement jumps paced at 1.0 to 1.1 jumps per second.	Researcher-supervised supplemental sessions before regular aerobic gymnastics training; once-weekly or twice-weekly dose.	Regional club training context and quiet-room force-platform assessment.	Pre- and post-intervention assessments over 8 weeks; one or two jumping-interval sessions/week; 4–5 sets of 30–40 s with 30-s recovery.
(Ma et al., 2024b)	High training frequency versus standard training frequency exposure, monitored with session-RPE and weekly ForceDecks squat jump, countermovement jump, and land-and-hold tests.	Real-world club training; researchers did not assign exposure; weekly testing before first session of each week after at least 24 hours rest.	Indoor club/team training context, exact sites not reported.	Weekly force-platform assessments over 4 weeks; high-frequency training during weeks 2-3; total exposure 20 sessions/2295 min versus 16 sessions/1845 min.
(Mehrtash et al., 2015)	Six-month specific aerobic gymnastics training plus repeated motor-ability field test battery.	National coach-supervised group training; routine, difficulty element, choreography, full-routine, and general physical preparation content.	Aerobic gymnastics training context in Iran; exact facility not reported.	Seven testing occasions, including baseline, across 6 months; 5 sessions/week, 3 h/session.
(Niu, 2022)	Virtual-reality aerobics simulation system for formation/pattern design, action generation, formation-change simulation, and movement-standardization feedback.	System-assisted training; delivery personnel and rater details not fully reported.	Virtual-reality simulation/training environment; exact site not reported.	Pre- and post-intervention assessments over an 8-week applied teaching period; session frequency and duration not reported.
(Panfilova, 2025)	NS-Psychotest.Sport computerized psychophysiological battery with Response to a Moving Object, Simple Hand-Eye Response, Distraction Tolerance, Tapping Test, and Acoustic-Motor Test.	Individual testing in a quiet private room before training; standardized computerized stimuli and hand-eye analyzer responses.	Gym/private-room psychophysiological testing context.	One 10-15-min off-season testing session.
(Mihaela and Dragomir, 2021)	Three-set maximal vertical-jump protocol combining OptoJump jump variables, heart-rate monitoring, and capillary blood lactate.	Athletes completed both-feet, right-foot, and left-foot maximal vertical jumping sets; heart rate recorded during effort and lactate measured after effort.	Training-room field/instrumented testing setting.	One testing session; three 30-s maximal-jump sets with 15-s recovery; lactate measured 2 min 30 s after exercise.
(Sang, 2023)	Functional training intervention and Functional Movement Screen/special physical fitness testing.	Experimental group received functional training in addition to standard training; control group received standard training only. FMS and special fitness tests were conducted pre and post.	Institute/team training and testing context; exact site not reported.	Pre- and post-intervention assessments over 12 weeks; session frequency, duration, and progression not reported.
(Sun, 2022)	Specialized core strength training with squats, weight-bearing squats, supine hip exercise, one-arm push exercise, abdominal-control exercise, push-ups, and upper-body strength work.	College/university training context; supervision and exact coach role not reported; both groups reportedly performed standard training.	College or university aerobics training/testing context.	Pre- and post-intervention assessment; 3–4 sessions/week, 1.5 h/session; total intervention duration not reported.
(Tibenská and Medeková, 2014)	Anthropometric and body-composition measures plus an eight-test motor-performance battery converted to Z-scores.	Standard anthropometric and motor testing during two-year sport preparation; exact assessors and club setting not reported.	Junior aerobic gymnastics training/testing context; exact club not reported.	Testing every 3 months across two 1-year preparation cycles; athletes trained five times/week for 120 min.
(Todorova et al., 2021)	Two-cycle annual training-cycle model emphasizing general physical preparation, special physical preparation, technical preparation, choreography, acrobatics, and author special exercise complexes.	Coach-led youth sports school training; main group received author model, control group followed standard program.	Municipal youth sports school No. 13, Kharkiv, Ukraine.	Pre- and post-assessment across one annual macrocycle; methods report six 150-min sessions/week, while the training model varies 3–6 days/week, 1–2 sessions/day, and 120–160 min/session.
(Trisanda et al., 2022)	Single-leg speed-hop plyometric training and standing broad jump field testing.	Delivered to Riau aerobic gymnastics athletes; supervision and attendance details incomplete.	Rumbai gymnastics hall, Pekanbaru, Indonesia.	Pre- and post-assessment across 12 training meetings; meeting frequency and duration not reported.
(Viktorovich and Vladimirovna, 2019)	UPFT-1/30-Psychophysiologist diagnostic system for readiness, psychomotor, tremometry, and psychological monitoring.	Individual diagnostics across training/preparation stages with progressively reduced test battery.	Russian national-team preparation locations: Omsk, Moscow, and Wroclaw.	Three monitoring occasions across preparation and competition stages; exact calendar interval not reported.
(Wang, 2015)	Fuzzy comprehensive body-function model with body function, body shape, and physical-quality components.	Model-based evaluation; exact assessors, measurement sources, and participant recruitment not reported.	China; exact testing context not reported.	One cross-sectional/modeling assessment; no intervention or follow-up.
(Wu, 2023)	Special lower-limb strength training plus core composite/posture assessment model.	Sports-college training/testing context; supervision and training content details incomplete.	Sports-college setting in China.	Pre- and post-assessment from March to July 2021; session frequency, duration, and exercise progression not reported.
(You, 2023)	Abdominal/core strength intervention versus traditional waist and abdominal training.	Training-base delivery with expert coach scoring; exact exercise list/fidelity not reported.	Competitive aerobics training base in China.	Pre- and post-intervention assessments over 12 weeks; session frequency, duration, intensity, and progression not reported.
(Yu and Liu, 2024)	Integrated C715 biomechanical assessment using Vicon motion capture and Noraxon surface electromyography.	Individual lab testing after standardized warm-up; three trials; two national judges scored trials; best/least-deduction trial analyzed.	Laboratory-based motion-capture and surface-electromyography context.	One laboratory testing session; three C715 trials per athlete.
(Yudho et al., 2024)	Wearable wireless inclinometer mounted at lower sternum to record sagittal and frontal body tilt.	Individual sensor testing with Bluetooth transmission every 0.1 s.	Wearable-sensor testing context; exact site not reported.	One sensor-testing session; three tasks, approximately 10 s per task.
(Yusupava et al., 2021)	Expanded complex preparedness test battery combining anthropometry, morphofunctional indicators, physical tests, technical element tests, musicality, and special endurance indicators.	Field-based assessment in a youth sport-school context; details on assessor training, blinding, and test order were not reported.	Children’s and youth sports school No. 13, Kharkiv, Ukraine.	One cross-sectional multidimensional assessment; session duration and number of trials per test not reported.
(Zhang, 2023)	Suspension rehabilitation training intended to challenge trunk and limb coordination, compared with traditional stretching recovery.	Training groups were reportedly maintained under similar daily routines and diet; supervision, adherence, and progression were not described.	Rehabilitation or training setting; exact site not reported.	Pre- and post-intervention assessments over 8 weeks; 3 sessions/week; session duration and load progression not reported.
(Zhao et al., 2025)	Multimodal wearable-sensor system using inertial measurement units, instrumented plantar-pressure insoles, and wireless surface electromyography, followed by deep feature extraction and Bayesian hierarchical ordered risk modeling.	Testing occurred during regular training sessions; sensors were synchronized at 200 Hz and placed bilaterally on lower-limb segments and muscles with lumbosacral sensing.	University and regional professional team sport-specific training/testing context in China.	Sport-specific sensor testing (two full 90-105-s routines and three sets of each high-risk sequence, with 3–5 min rest) plus 6-month in-season injury surveillance.

The most frequently represented tool family was strength, power, and physical-fitness assessment, addressed in 19 reports. Training-intervention and exposure-monitoring evidence was present in 17 reports; balance, flexibility, coordination, and functional-movement assessment in 11; and anthropometric, biomechanical, and psychophysiological or digital-system methods in nine reports each. Physiological, metabolic, and cardiorespiratory methods were represented in five reports, and anaerobic or sport-specific performance testing in four.

The most common outcome domain was strength, power, morphology, and functional preparedness, addressed in 28 reports. Balance, flexibility, coordination, and motor-control outcomes occurred in 19 reports; biomechanical loading, technical performance, and digital outputs in 15; physiological, aerobic, and anaerobic responses in ten; injury, training-load, and psychophysiological readiness in nine; and measurement properties in seven.

**Figure 1** presents the evidence map by assessment/monitoring method or exposure group and outcome domain. The 50 included reports generated 107 report–cell contributions because a single report could contribute to more than one cell, although it was counted no more than once within each cell. The counts therefore represent the distribution of published reports and not independent participants, cohorts, comparisons, tools, interventions, or effect estimates.

The largest concentration involved strength, power, and physical-fitness assessment mapped against strength, power, morphology, and functional-preparedness outcomes (16 reports). Training-intervention and exposure-monitoring evidence mapped against the same outcome domain was the second-largest cell (13 reports). Biomechanical, kinematic, technical, and loading analyses were most frequently linked to biomechanical loading, technical performance, and digital outputs (9 reports). Anthropometric and morphological assessment mapped against morphology and functional preparedness, and balance and functional-movement assessment mapped against balance, flexibility, coordination, and motor control, were each represented by eight reports. Physiological, metabolic, and cardiorespiratory methods were primarily linked to physiological, aerobic, and anaerobic responses (five reports).

Several cells contained no eligible report, indicating combinations for which no explicit aerobic-gymnastics evidence was identified. These zeros should be interpreted as gaps in the literature retrieved through the review procedures rather than evidence that the corresponding method–outcome relationship is absent.

### 3.3 Methodological appraisal within included studies

Critical-appraisal findings are summarized by study design and methodological purpose in **Table 4**, with complete item- and domain-level judgments in the **Supplementary Material**. The 50 included reports generated 51 appraisal units because the wearable-sensor study by Luo et al. (2025) was evaluated separately for its randomized intervention effect and its algorithmic classification component. JBI judgments were retained at the item level and were not converted into custom global concern categories.

**Table 4:** Methodological appraisal frameworks and principal findings.

Study design or methodological purpose	Appraisal framework and version	Studies, n	Judgment level reported	Principal recurring methodological concerns	Complete appraisal
Randomized parallel-group intervention effects	Cochrane RoB 2, parallel-group version	3	Result-level appraisal across five domains; signaling questions and domain/overall judgments reported	Incomplete allocation-concealment or protocol information, adherence/deviation reporting, missing-data handling, and selection of reported results	**Supplementary Table 5**
Quasi-experimental intervention or exposure studies	JBI Checklist for Quasi-Experimental Studies	17	Item-level Y/N/U/NA; no numerical score or global concern category	Unclear allocation and comparator procedures, single pre–post assessments, incomplete follow-up and fidelity reporting, and limited evidence of outcome-measure reliability	**Supplementary Table 2D**
Cohort studies	JBI Checklist for Cohort Studies	3	Item-level Y/N/U/NA; no numerical score or global concern category	Residual confounding, incomplete reporting of measurement reliability and follow-up, and limited handling of missing data	**Supplementary Table 2A**
Analytical cross-sectional studies	JBI Checklist for Analytical Cross-Sectional Studies	14	Item-level Y/N/U/NA; no numerical score or global concern category	Convenience or narrowly selected samples, limited identification/control of confounding, and insufficient reporting of measurement validity or reliability	**Supplementary Table 2E**
Case series	JBI Checklist for Case Series	4	Item-level Y/N/U/NA; no numerical score or global concern category	Unclear consecutive/complete inclusion, limited setting and sampling detail, and absent reliability or repeatability evidence	**Supplementary Table 2B**
Case reports	JBI Checklist for Case Reports	2	Item-level Y/N/U/NA; no numerical score or global concern category	Incomplete athlete history/timeline, limited follow-up or harm reporting, and restricted causal and generalizable interpretation	**Supplementary Table 2C**
Measurement-property studies	COSMIN taxonomy; COSMIN reliability/measurement-error tool; explicitly adapted criteria for content/construct/criterion validity and responsiveness	4	Each measurement property evaluated separately; criterion-level findings reported; no standard full COSMIN rating or aggregate study score	Small samples, incomplete repeat-test standardization, scarce measurement-error thresholds, lack of acceptable reference standards, and intervention-related change being more common than formal responsiveness evidence	**Supplementary Tables 3A, B**
Prediction-model or algorithmic components	PROBAST	4	Twenty signaling questions, four domain judgments, overall risk of bias, and applicability concerns	Small development samples, incomplete participant/model specification, limited missing-data and calibration reporting, overfitting risk, and absence of independent external validation	**Supplementary Tables 4A–C**

Across the 17 quasi-experimental studies, recurring limitations involved inadequately described allocation and comparator procedures, limited control of co-interventions and maturation, reliance on a single pre-intervention and post-intervention assessment, incomplete follow-up or fidelity reporting, and insufficient evidence that outcome measures were applied reliably. The 14 analytical cross-sectional studies were most often limited by convenience or narrowly selected samples, incomplete identification or control of confounding, and sparse reporting of measurement validity or reliability. Cohort studies were principally limited by residual confounding, incomplete follow-up or missing-data procedures, and measurement reporting, whereas case reports and case series often lacked clear consecutive or complete inclusion and detailed longitudinal context.

Four reports contributed explicit measurement-property evidence. The SAGAT had the most developed preliminary evidence, including test–retest reliability, a minimal detectable change estimate, and convergent associations with anaerobic-performance measures. Its score also changed between preparatory and competition phases. However, this phase-related difference was classified as observed training-cycle change rather than formal responsiveness because the study did not report a sufficiently explicit longitudinal-validity design, prespecified responsiveness hypotheses, or an external change anchor. Formal responsiveness was therefore not established for the SAGAT or for any other included instrument. The other measurement-property reports provided content-validity, rater-reliability, agreement, or method-comparison evidence but did not formally evaluate responsiveness. Across the broader evidence base, changes after interventions or longitudinal exposure were coded separately from measurement properties.

All four prediction-model or algorithmic studies were judged at high overall risk of bias under PROBAST because the analysis domain contained important concerns, including small development samples, incomplete handling of missing data, limited calibration reporting, risk of overfitting, internal validation only, and absence of independent external validation. The three randomized parallel-group trials were judged as having some concerns overall, mainly because of incomplete allocation concealment or protocol information, limited adherence and deviation reporting, missing-data handling, or selective-reporting uncertainty.

### 3.4 Results of individual studies

The results of individual studies are summarized in **Table 5**. At the individual-study level, measurement-property evidence was most developed for the Specific Aerobic Gymnastics Anaerobic Test, which reported concurrent associations with Wingate-derived anaerobic indices, high test–retest reliability, minimal detectable change, and sensitivity to training cycle change. Other measurement property evidence was narrower, including content validity or rater reliability for flexibility testing, method-comparison evidence for body-composition assessment, and exploratory reliability or agreement statistics for selected digital or instrumented tools. Physiological studies linked competitive routines or sport-specific tests to oxygen uptake, heart rate, blood lactate, energy-system contribution, and aerobic or anaerobic fitness variables. Biomechanical studies primarily described kinematic errors, landing forces, loading patterns, joint moments, stiffness, movement symmetry, or technical execution parameters, frequently in small or highly selected samples.

**Table 5:** Results of individual studies.

Study	Main comparison made	Main statistical values related with the main outcomes	Main findings
(Abalo-Núñez et al., 2018)	Injured versus uninjured aerobic gymnasts; predictive logistic regression models for season injury status using anthropometric and biomechanical variables.	Left quadriceps angle, body weight, and interaction model: left quadriceps angle odds ratio 5.075, 95% confidence interval 1.413 to 18.230, *p* = 0.013; body weight odds ratio 1.480, 95% confidence interval 1.044 to 2.099, *p* = 0.028; left quadriceps angle by weight odds ratio 0.974, 95% confidence interval 0.952 to 0.997, *p* = 0.026; AIC = 45.47; specificity 97.4%, sensitivity 58.3%, global correct classification 88.2%. Earlier model: left quadriceps angle odds ratio 1.298, *p* = 0.021; right-leg bilateral weight-bearing odds ratio 1.393, *p* = 0.060; left thigh perimeter odds ratio 0.762, *p* = 0.083; AIC = 47.48. Mann-Whitney U *p*-values: right quadriceps angle 0.005, left quadriceps angle 0.003, right-leg bilateral weight-bearing 0.025.	The study concludes that excessive quadriceps angle, especially the left quadriceps angle, may predispose competitive aerobic gymnasts to lower-limb injury, with body weight modifying the association.
(Albano et al., 2021)	Descriptive proposal and example output for force-platform tests in two aerobic gymnasts.	Explosive Push Up Test example: peak force total 1.392 N/kg, right 0.691 N/kg, left 0.700 N/kg; rate of force development total 7.636 N/s/kg, right 3.798 N/s/kg, left 3.855 N/s/kg; symmetry index 1.099; contact time 0.972 s; flight time 0.28 s.	The study concludes that Push Up Jump Test, Explosive Push Up Test, and Free Fall Test may provide useful information on upper-body force expression, bilateral asymmetry, performance monitoring, and injury prevention. The authors explicitly state that larger-sample validation is required.
(Aleksandraviciene et al., 2015)	International-level versus national-level female aerobic gymnasts during treadmill testing and competitive group routine.	Peak oxygen uptake: 45.4 ± 3.9 versus 44.7 ± 3.6 ml/kg/min, *p* = 0.81. Competitive routine oxygen uptake: 37.2 ± 3.7 versus 41.4 ± 3.4 ml/kg/min, *p* = 0.06; oxygen uptake as percentage of peak oxygen uptake: 80.1 ± 6.5% versus 92.8 ± 7.3%, *p* = 0.01. Lactate: 7.52 ± 2.07 versus 6.08 ± 1.39 mmol/L, *p* = 0.35. Net total energy: 1847.7 ± 293.9 versus 1747.3 ± 196.7 J/kg, *p* = 0.72. Net aerobic fraction: 53.5 ± 3.1% versus 60.3 ± 6.1%, *p* = 0.03; anaerobic alactic fraction: 25.4 ± 5.9% versus 21.4 ± 5.2%, *p* = 0.19; anaerobic lactic fraction: 21.1 ± 5.8% versus 18.3 ± 4.5%, *p* = 0.41.	Aerobic fitness and absolute energetic/physiological responses were generally similar between performance levels, but international-level gymnasts showed a higher relative anaerobic contribution during more difficult competitive routines.
(Alves et al., 2015)	Specific Aerobic Gymnast Anaerobic Test compared with Wingate tests, simulated competition, repeated trials, and training-cycle change.	Concurrent validity correlations: lower-body Wingate mean power *r* = −0.69, *p* = 0.03; lower-body peak power *r* = −0.72, *p* = 0.02; upper-body mean power *r* = −0.67, *p* = 0.03; upper-body peak power *r* = −0.69, *p* = 0.03. Reliability: intraclass correlation coefficient = 0.97, 95% confidence interval 0.89 to 0.99; test–retest *p* = 0.84; Cohen’s d = 0.09; average variation 0.36 s or 0.44%; 95% minimal detectable change = 0.12 s. Sensitivity: before preparatory cycle 92.5 ± 8.3 s versus competition cycle 86.1 ± 5.0 s, *p* < 0.001.	Specific Aerobic Gymnastics Anaerobic Test was reported as a valid, reliable, and sensitive sport-specific anaerobic performance test for elite female aerobic gymnasts, with lactate response similar to simulated competition and lower-body Wingate testing.
(Artemieva et al., 2019)	Special physical training methodology versus usual youth sports-school curriculum, assessed before and after a one-year training period.	Main group: vital capacity +5.37%, *p* < 0.01; Harvard Step Test Index +5.39%, *p* < 0.01; resting heart rate -2.96%, *p* < 0.05. Selected main-group motor improvements: push-ups +30.67%, *p* < 0.01; torso raises +12.65%, *p* < 0.01; wall-sit +5.86%, *p* < 0.01; running on spot +4.14%, *p* < 0.05; arm raising/lowering +18.07%, *p* < 0.01; forward bends +18.56%, *p* < 0.01; deep-squat jumps +10.52%, *p* < 0.05; alternate 90° leg swings +9.18%, *p* < 0.05. Flexibility changes were 0.2% to 1.0% and nonsignificant.	The methodology was reported to improve functional preparedness and several motor qualities in 7- to 8-year-old sports-aerobics gymnasts more than the usual program, while flexibility showed limited change likely because initial flexibility was already high.
(Ayziaatullova et al., 2022)	L-sit hold time and correlations with anthropometric body-segment ratios in sports-aerobics athletes aged 9 to 11 years.	L-sit time by dimension type: short legs-long arms 24.2 ± 9.3 s; long legs-short arms 11.7 ± 6.6 s; long legs-long arms 14.3 ± 6.9 s; short legs-short arms 21.7 ± 11.4 s; average legs-average arms 22.6 ± 8.4 s. Statistically significant correlations with L-sit: height-to-arm length ratio *r* = −0.506, *p* ≤ 0.05; leg length-to-foot length ratio *r* = 0.535, *p* ≤ 0.05.	The study concluded that body dimensions meaningfully affect acquisition/performance of the L-sit static-strength element, supporting individualized exercise selection based on anthropometric characteristics.
(Bota and Urzeală, 2013)	Functional stress by technical element during routines and initial versus final neuromuscular-control score after Ergosim training.	Heart-rate examples: Wenson hinge push-up 155–160 beats/min; switch split leap to split 185–187; helicopter to split 185–187; straddle support 1 1/2 turn 190–194; 1 1/2 turn tuck jump 191–193; free illusion 194–196; 1 1/2 turn to vertical split 196–198; L-support 2/1 turn or more 196–199. Neuromuscular-control mean score improved from 6 to 8.11; Six Sigma level improved from 0 to 3, corresponding to 93% probability of efficient/error-free movement.	Heart-rate monitoring indicated hard to above-maximum routine intensity, especially in the second half of the routine. Simulator-based training was reported to improve neuromuscular control in movements involving muscles used in difficult elements.
(Bota et al., 2014)	Observed executions of C104 1 1/2 air turn and C345 1/1 turn cossack jump compared with technical execution criteria using Xsens MVN motion capture.	No p values or group statistics reported. Descriptive kinematic findings included whole-body inclination and center-of-mass displacement during C104, foot/ankle rotations and compensatory trunk/pelvis movement during take-off preparation, and for C345 small angular values between thigh and horizontal plane plus values less than 60° between trunk and the reference plane.	The study concluded that MVN BIOMECH can provide useful kinematic parameters for detecting execution deficiencies, visualizing technical errors to coaches and athletes, identifying possible causes, and reducing correction time.
(Budiarti et al., 2022)	Content-validity and interrater-reliability assessment of a proposed aerobic-gymnastics flexibility test for the national development category.	Aiken’s V for 12 items ranged from 0.75 to 1.00, all above the stated validity threshold of 0.70. Cronbach’s α = 0.919. Single-measures intraclass correlation coefficient = 0.507, 95% confidence interval 0.303 to 0.763, *F* = 12.296, *p* < 0.001. Average-measures intraclass correlation coefficient = 0.911, 95% confidence interval 0.813 to 0.970, *F* = 12.296, *p* < 0.001.	The proposed flexibility test was considered valid and reliable by expert/rater assessment.
(Chayun, 2024)	Age-category comparison of female aerobic gymnasts aged 15–17, 12–14, and 9–11 years on psychophysiological tests.	CVMR-LSC mean reaction time: 15–17 years 583 ± 102 ms, 12–14 years 658 ± 138 ms, 9–11 years 796 ± 227 ms. FMNP total stimuli: 15–17 years 248 ± 28, 12–14 years 231 ± 19, 9–11 years 187 ± 17; correct responses 136 ± 17, 126 ± 11, and 102 ± 8, respectively. RMO mean deviation: 48 ± 13 ms, 46 ± 10 ms, and 63 ± 12 ms respectively. Authors report *p* < 0.05 for total stimuli difference between athletes aged 12 years or older and those aged 9–11 years.	Older athletes generally showed faster reaction time and higher functional mobility. Most athletes displayed a shift of nervous processes toward inhibition. The report proposes model characteristics for aerobic-gymnastics psychophysiological monitoring but does not validate the device for this sport.
(Chayun and Sharovarova, 2025)	Within-athlete longitudinal comparison across February 2022, February 2023, and August 2023 for three female youth aerobic gymnasts.	maximal oxygen uptake ml/min/kg: Athlete 1 43.2, 36.3, 40.3; Athlete 2 41, 36, 37; Athlete 3 36, 36.9, 36.6. Aerobic threshold VO2 ml/min/kg: Athlete 1 32, 30, 34; Athlete 2 30, 25, 25.4; Athlete 3 31, 28, 28.5. Anaerobic threshold VO2 ml/min/kg: Athlete 1 37, 35, 38; Athlete 2 40, 32, 30; Athlete 3 35, 35, 35. Athlete 2 reached aerobic threshold at 10 km/h and anaerobic threshold at 12 km/h in the first test.	Athletes differed in functional trajectories during puberty. Athletes 1 and 2 showed lower maximal oxygen uptake in February 2023 followed by partial recovery; Athlete 3 remained at a low maximal oxygen uptake level. Findings support a decline or heterogeneity of functional capacity during puberty and emphasize individualized monitoring.
(Chen, 2025)	Measured versus predicted energy expenditure and metabolic equivalents; sex and accelerometer-wear-location comparisons.	Substrate proportions: carbohydrate 71.2%, fat 19.6%, protein 9.1%. MET correlations included right waist vector magnitude *r* = 0.446, *p* < 0.01; left waist vector magnitude *r* = 0.431, *p* < 0.01; chest vector magnitude *r* = 0.359, *p* < 0.01. Text reports right waist vector magnitude correlation with measured energy expenditure *r* = 0.733. Prediction-model verification: no significant predicted-versus-measured difference, *p* > 0.05; energy-expenditure error 4.42% for males and 3.39% for females; metabolic-equivalent error 4.40% for males and 1.77% for females; coefficient of determination higher than 0.7.	Competitive aerobics individual events were high-intensity and carbohydrate-dominant. Right waist accelerometer placement was identified as most useful for energy-consumption prediction. The model showed promising internal accuracy but requires external and practical training-environment validation.
(D’Anna et al., 2019)	Within-athlete comparison of equal-feet pre-jump technique with flight phase versus without flight phase across Tuck Jump, Cossack Jump, and Straddle Jump.	Jump height: 53.56 ± 8.65 cm with flight phase versus 50.09 ± 8.28 cm without flight phase, *p* = 0.001, eta squared = 0.544. Flight time: 0.599 ± 0.055 s versus 0.585 ± 0.055 s, *p* = 0.023, eta squared = 0.339. Knee angle: no overall main effect; interaction *p* = 0.030, eta squared = 0.417; Straddle Jump *post-hoc p* = 0.045, eta squared = 0.585.	The pre-jump with a flight phase produced greater jump height and flight time in this small senior-athlete pilot. The study concluded that this may matter for more complex jumps requiring greater rotation, while emphasizing the need for larger samples and higher-speed analysis.
(Ding, 2023)	Elastic-band training versus routine training for three-step approach-run first-level right-step jump performance.	Experimental group: 170.23 cm to 174.78 cm, change 4.55 cm, 2.67%, paired *t* = −2.96, *p* = 0.034. Control group: 169.82 cm to 172.52 cm, change 2.70 cm, 1.59%, paired *t* = −1.95, *p* = 0.251. Post-intervention between-group *t* = 2.17, *p* = 0.186. Baseline fitness comparisons reported as all *p* > 0.05, exact values not reported.	The experimental group improved significantly within group, but the between-group post-intervention comparison was not statistically significant.
(Filippova et al., 2006)	Female sports-aerobics athletes versus non-athletic controls across five age groups for morphology, cardiorespiratory function, respiratory function, and physical fitness.	PWC170/kg significantly higher from age 11; maximum oxygen consumption significantly higher from age 13; recovery index higher from age 16; double product under load significantly lower only at age 19–22. Sport-specific fitness: push-ups, L-position hold, and flexibility were significantly higher in all age groups (*p* < 0.001), and standing long jump was significantly higher (*p* < 0.05). Example 19–22 years: maximum oxygen consumption 49.44 ± 2.79 versus 34.81 ± 0.83 ml/min/kg; PWC170/kg 14.46 ± 0.65 versus 10.41 ± 0.42; push-ups 33.1 ± 1.9 versus 14.8 ± 1.6; L-position 33.2 ± 4.8 versus 0.4 ± 0.3 s.	The study concluded that sports aerobics profoundly influences cardiorespiratory function and physical fitness and has smaller but age/ranking-related effects on morphology.
(Ge et al., 2025)	High-level versus low-level competitive aerobics athletes during 360° straight-body rotational jump landing.	Anterior tibial shear force was greater in low-level athletes from 12.87% to 26.86% of the landing phase (*p* = 0.005). Patellofemoral joint contact force was greater in high-level athletes from 0% to 0.94% (*p* = 0.049) and 8.06% to 13.39% (*p* = 0.034). Peak muscle activation differences included rectus femoris *p* = 0.002, vastus medialis *p* < 0.001, biceps femoris *p* < 0.001, vastus lateralis *p* = 0.003, soleus *p* < 0.001, and tibialis anterior *p* = 0.015.	Low-level athletes showed poorer ankle and knee stability and higher anterior cruciate ligament and ankle-inversion injury-risk profiles during rotational jump landings.
(Guo et al., 2026)	Table tennis players, action video game players, aerobic gymnastics athletes, and non-trained college students across modified Useful Field of View subtests.	Subtest 1: *F*(3, 100) = 1.32, *p* = 0.271. Subtests 2–3 mixed analysis: subtest main effect *F*(1,100) = 374.17, *p* = 1.428 × 10^−35; eccentricity *F*(1,100) = 194.62, *p* = 3.353 × 10^−25; group *F*(3,100) = 14.33, *p* = 7.775 × 10^−8; subtest-by-group interaction *F*(3,100) = 3.50, *p* = 0.018. Subtest 2 group effect *F*(3,100) = 8.72, *p* = 3.416 × 10^−5; aerobic gymnastics athletes versus non-trained students ΔM=-91.91 ms, p_holm = 0.013. Subtest 3 group effect *F*(3,100) = 10.36, *p* = 5.363 × 10^−6.	Aerobic gymnastics athletes showed a divided-attention advantage over non-trained controls but did not show a selective-attention advantage under strong distractor competition. Experience-related visual-attention advantages were component-specific rather than global.
(Hao et al., 2022)	Experimental group with core-strength training plus traditional training versus control group with traditional training.	Post-intervention stability values favored the test group in all eight reach directions (e.g., back stretch 0.98 ± 0.04 versus 0.90 ± 0.05; right back stretch 0.96 ± 0.06 versus 0.83 ± 0.02). Core-strength values favored the test group: swivel sit-ups 62.71 ± 6.50 versus 50.55 ± 3.24; prone back-up 118.32 ± 6.58 versus 100.21 ± 5.78; seat pull-down 10.55 ± 1.34 versus 8.45 ± 2.46; kick 7.21 ± 0.85 versus 6.78 ± 0.46.	The study concluded that core-strength training improved stability, core strength, and completion quality of Group C difficult movements.
(Kokarev et al., 2023a)	Innovative special physical/functional training versus traditional special physical training in qualified Ukrainian national-team aerobic gymnasts.	Stage 1 EG improvements: leg swings +6.7%, *p* < 0.05; flexibility +5.6%, *p* < 0.05; straddle L-support +11.6%, *p* < 0.01; feet lifting +13.1%, *p* < 0.01; push-ups +8.4%, *p* < 0.01; standing long jump +5.5%, *p* < 0.05; pistol squats +7.2%, *p* < 0.05. Stage 2 cross-experiment: flexibility, straddle L-support, feet lifting, and push-ups improved significantly in one or both groups; most final intergroup differences were nonsignificant except straddle L-support and feet lifting favoring the former experimental group.	The authors concluded that innovative fitness-training methods improved special physical fitness and are feasible for physical/functional training during preparatory periods. Extraction flags unclear allocation and incomplete adherence/blinding reporting; study counted independently from the related technical-fitness report.
(Kokarev et al., 2023b)	Experimental technical training program using 6-D Sliding, Procedos, EXOS, and light platform versus classical aerobic-gymnastics training program.	End preparatory/end competition between-group differences favored EG for straddle jump (+30.14%, *p* < 0.01; +27.54%, *p* < 0.01), straddle L-support with 360° turn (+43.09%, *p* < 0.01; +40.33%, *p* < 0.01), lateral balance (+11.59%, *p* < 0.01; +10.00%, *p* < 0.05), jump with 360° turn (+39.03%, *p* < 0.01; +40.00%, *p* < 0.01), artistic readiness (+9.21%, *p* < 0.05; +10.49%, *p* < 0.01), and choreographic-compositional readiness (+10.58%, *p* < 0.05; +9.67%, *p* < 0.05). Vertical split was significant at end preparatory (+13.85%, *p* < 0.01) but not at end competition (+9.29%, *p* > 0.05).	The study concluded that the experimental program improved technical, artistic, and choreographic-compositional preparedness and maintained higher levels through the competitive period.
(Kozina et al., 2017)	Within-cohort factor analysis of integral readiness indicators in female sports-aerobics athletes.	Four factors were identified: parasympathicotonia 32.5%; mobility of the nervous system 27.6%; force 22.14%; sense of time 16.06%; other factors 1.7%. Key loadings included autonomic/heart-rate variables, reaction-time variables, jump/strength/anthropometric variables, and 1-s time-interval reproduction error.	The study concluded that factor analysis can determine the overall and individual structure of athletes’ readiness and may support grouping athletes for competitive categories.
(Kyselovičová et al., 2023)	Aerobic, artistic, and rhythmic elite adolescent female gymnasts compared on Y-Balance and Biodex isokinetic outcomes; within-aerobic subgroup correlations between dynamic balance and isokinetic strength.	Y-Balance: composite dominant *F* = 3.536, *p* = .041; composite non-dominant *F* = 4.804, *p* = .015; anterior dominant *F* = 4.933, *p* = .023. Average power during knee extension/flexion at all tested velocities differed among groups, with *p*-values from.049 to.004. Peak torque/body weight did not differ significantly. In aerobic gymnasts, Y-Balance dominant composite correlated with dominant extension strength at 60°/s *r* = .54, 180°/s *r* = .87, and 300°/s *r* = .84.	Dynamic balance and isokinetic strength-power show gymnast specific patterns. In aerobic gymnasts, dynamic balance appears linked to dominant knee-extension strength across angular velocities, supporting strength and fast-speed monitoring.
(Kyselovičová and Zemková, 2024)	Within-athlete 24-month changes in a comprehensive performance-monitoring battery in one elite aerobic gymnast.	Y-Balance composite dominant 98.35 to 103.09% (+4.8%); non-dominant 109.47 to 112.76% (+3.0%). IMOOVE trunk stability 62 to 78 (+16%); postural coordination 35 to 43 (+8%); support stability 100 to 88 (−12%). Repeated-jump fatigue index 42% to 22% (−47.6%). Dominant extension mean power increased by 23.2% at 60°/s and 18.5% at 180°/s. Wingate mean power was essentially unchanged (444.50 to 444.05 W) and fatigue index increased (42.51% to 48.82%).	Over two years, dynamic balance, postural coordination, repeated-jump endurance, and selected isokinetic leg-extension variables improved, while vertical explosive power and Wingate anaerobic performance did not.
(Lamošová et al., 2020)	Within-athlete comparison of Free illusion to vertical split versus Free illusion to free vertical split using three-dimensional kinematic analysis.	First phase duration: 1.30 s for both elements; second phase differed by 0.02 s in favor of faster FIVS. Leading-leg kinematics: FIVS phase 1 amax 64.14 m/s² and vmax 7.72 m/s; FIFVS phase 1 amax 65.13 m/s² and vmax 8.02 m/s; FIVS phase 2 amax 42.39 m/s² and vmax 6.06 m/s; FIFVS phase 2 amax 45.07 m/s² and vmax 5.70 m/s. Split angle: FIVS 177.77°; FIFVS 161.31°; minimum requirement 170°. Standing-leg angle: FIVS 83.04°; FIFVS 77.58°.	The analysis identified element-specific technical errors. FIVS met the minimum split-angle requirement but had posture and transition errors; FIFVS failed to meet the split-angle requirement and showed larger standing-leg-position error.
(Lamošová et al., 2021)	Within-cohort one-year pre–post change in anthropometry and motor fitness in young female aerobic gymnasts.	Body height 130.21 ± 9.21 to 135.86 ± 9.41 cm, p ≤ 0.01; body weight 26.88 ± 6.10 to 30.30 ± 5.96 kg, *p* ≤ 0.01; body mass index 15.59 ± 1.57 to 16.29 ± 1.51, *p* ≤ 0.01. 4 × 10 m shuttle run 12.78 ± 0.67 to 12.02 ± 0.77 s, *p* ≤ 0.01; standing long jump 142.78 ± 16.38 to 153.96 ± 19.51 cm, *p* ≤ 0.01; straddle support 13.58 ± 8.81 to 19.54 ± 10.23 s, *p* ≤ 0.05; back extension 36.87 ± 10.55 to 45.83 ± 25.58, *p* ≤ 0.05; modified push-ups 17.70 ± 3.67 to 21.35 ± 4.50, *p* ≤ 0.01; sit-ups 39.43 ± 4.94 to 43.87 ± 7.32, *p* ≤ 0.01.	Regular one-year aerobic gymnastics exposure was associated with growth and significant improvements in several field motor tests.
(Maria Laroëre et al., 2024)	External focus versus internal focus versus no-focus control during L-support execution.	Main effect of attentional focus: *F*(2,20) = 14.76, *p* <.001, partial eta squared = 0.57. External focus M = 0.21 ± 0.17 deductions; internal focus M = 0.27 ± 0.20; control M = 0.32 ± 0.20. External focus versus internal focus *p* = .002, *d* = 1.27; external focus versus control *p* = .002, *d* = .96; internal focus versus control *p* = .196. Inter-rater intraclass correlation coefficient = .905, 95% confidence interval.807–.931, *p* <.001.	External focus instructions improved immediate L-support movement form by reducing execution faults compared with internal focus and no-focus conditions.
(Li, 2025)	New AI/IIoT training decision system versus traditional training decision-evaluation system.	Body control ability: 78.0 ± 5.2 versus 91.0 ± 4.7, *t* = 16.54, *p* < 0.001. Training difficulty: 81.0 ± 5.6 versus 96.0 ± 4.2, *t* = 17.23, *p* < 0.001. Performance style: 75.0 ± 6.1 versus 86.0 ± 5.1, *t* = 14.67, *p* < 0.001. Average reported training-effect increase 16.63%. Satisfaction: group A 87%, B 83%, C 90%, D 85% satisfied.	The study concludes that an AI/IIoT training decision system can improve scientific and personalized competitive-aerobics training.
(Lopes et al., 2024)	Body composition at M1 versus M2 and comparison of skinfold, ultrasound, and bioimpedance body-fat estimates.	Bioimpedance composition: body mass 53.5 [50.9–57.5] kg at M1 versus 54.0 [50.8–57.5] kg at M2, *p* = 0.483; body fat percentage by BIA 21.1% [19.1–22.8] versus 21.5% [19.4-23.7], *p* = 0.178. Body fat by skinfolds 15.3% [13.7-19.3] versus 15.2% [14.1–18.4], *p* = 0.688; ultrasound 21.1% [19.5–24.3] versus 19.3% [17.8–20.9], *p* = 0.063. Skinfolds differed from bioimpedance at M2, *p* = 0.03. Bland-Altman agreement only between ultrasound and bioimpedance.	Body composition did not change significantly across 30 days. Skinfolds may underestimate body fat compared with ultrasound and bioimpedance; absence of a gold-standard reference prevents selecting the best method.
(Luo et al., 2025)	Digitalized intelligent training program versus conventional coach-centered training, structured progressive technical training, and high-intensity conditioning-oriented training; algorithm validation also reported.	Algorithmic metrics: accuracy, precision, recall, and F1-score all above 0.90; average latency below 200 ms. countermovement jump peak vertical ground reaction force interaction *F*(3,32) = 19.8, *p* < 0.001, partial eta squared = 0.65; DITP + 11.8 ± 2.4%, CON + 2.1 ± 1.7%, P-I +6.3 ± 2.0%, P-II +4.0 ± 1.9%. countermovement jump maximum power *F*(3,32) = 21.3, *p* < 0.001, partial eta squared = 0.67; DITP + 14.6 ± 2.9%. Rate of force development *F*(3,32) = 17.4, *p* < 0.001, partial eta squared = 0.62; DITP + 18.2 ± 3.1%. Drop jump RSI *F*(3,32) = 16.0, *p* < 0.001, partial eta squared = 0.60; DITP + 15.3 ± 3.0%. CV of countermovement jump peak GRF interaction *F*(3,32) = 9.1, *p* < 0.001.	The report concludes that wearable-sensor and machine learning feedback can improve force-related neuromuscular performance and force-output stability more than conventional/progressive/conditioning training. Interpretation should note that exact raw means and exact algorithm metrics were not fully reported.
(Ma et al., 2024a)	Jumping interval training versus running high-intensity interval training versus usual training over 8 weeks.	Specific Aerobic Gymnastics Anaerobic Test interaction *F*(2,70) = 34.280, p < 0.001, partial eta squared = 0.495; jumping interval training 81.0 ± 1.4 to 79.4 ± 1.4 s; high-intensity interval training 81.2 ± 1.3 to 79.7 ± 1.1 s; control 81.1 ± 1.3 to 80.6 ± 1.1 s. countermovement jump interaction *F*(2,70) = 17.835, *p* < 0.001, partial eta squared = 0.338; jumping interval training 31.0 ± 1.6 to 32.5 ± 1.2 cm; high-intensity interval training 30.7 ± 2.4 to 31.2 ± 2.0 cm; control 30.9 ± 2.3 to 31.2 ± 1.8 cm. 20-m multistage interaction *F*(2,70) = 34.935, *p* < 0.001, partial eta squared = 0.500; jumping interval training 1307.5 ± 47.5 to 1424.2 ± 40.0 m; high-intensity interval training 1276.7 ± 49.6 to 1400.0 ± 40.0 m; control 1275.2 ± 55.5 to 1343.2 ± 51.5 m.	Both jumping interval training and high-intensity interval training improved aerobic and anaerobic performance more than usual training. Jumping interval training was the most sport-specific and produced superior countermovement-jump improvement.
(Ma et al., 2024c)	High training frequency congested period versus standard training frequency across four weekly assessments.	Interactions: countermovement jump peak power *F* = 126.855, *p* < 0.001, partial eta squared = 0.725; countermovement jump peak landing force *F* = 137.910, *p* < 0.001, partial eta squared = 0.742; SJ peak power *F* = 120.492, *p* < 0.001, partial eta squared = 0.715; SJ maximum negative displacement *F* = 55.048, *p* < 0.001, partial eta squared = 0.534; LHT time to stabilization *F* = 36.375, *p* < 0.001, partial eta squared = 0.431; LHT peak drop landing force *F* = 137.763, *p* < 0.001, partial eta squared = 0.742. Final HTF versus STF: countermovement jump peak power 28.2 ± 4.0 versus 32.1 ± 5.6 W/kg, *p* = 0.008; countermovement jump landing force 1630.5 ± 519.5 versus 1362.4 ± 375.3 N, *p* = 0.041; SJ peak power 6.21 ± 0.82 versus 7.24 ± 1.32 W/kg, p=0.002; SJ displacement 32.9 ± 10.6 versus 26.5 ± 7.3 cm, *p* = 0.015; LHT peak drop landing force 1296.3 ± 457.1 versus 1088.1 ± 240.4 N, *p* = 0.047.	Two congested weeks of high training frequency were associated with acute power decrements and increased landing forces, suggesting non-functional overreaching and potentially increased injury risk.
(Ma et al., 2024b)	Once-weekly jumping interval training versus twice-weekly jumping interval training versus usual aerobic gymnastics training control	Specific Aerobic Gymnastics Anaerobic Test interaction *F*(2,66) = 33.635, *p* < 0.001, ηp² = 0.505; countermovement jump interaction *F*(2,66) = 10.216, *p* < 0.001, ηp² = 0.236; 20-m multistage interaction *F*(2,66) =11.475, *p* < 0.001, ηp² = 0.258. twice-weekly jumping interval training group within changes: Specific Aerobic Gymnastics Anaerobic Test −1.554 s, countermovement jump +1.542 cm, 20-m +116.7 m.	Both jumping interval training frequencies improved aerobic, anaerobic, and jumping outcomes. Twice-weekly jumping interval training was generally preferable when larger improvements were desired, while once-weekly jumping interval training may be a minimal effective dose.
(Mehrtash et al., 2015)	Pre-training versus six months of specific aerobic gymnastics training	Chin-up +16.2%, legs lift in 30 s +65.2%, rope climb improved by 15.9%, tuck-up trunk and leg flexion +23.9%, single-leg squats +190.5% left and +106.8% right, horizontal jump +3.7%, press handstand +146.3%; reported *p* < 0.05 for significant changes. Legs lift from piked position was not significant.	Six months of specific aerobic gymnastics training was associated with improved motor ability in boys aged 10–12 years, but causal inference is limited by absence of a control group.
(Niu, 2022)	VR simulation training versus control training after 8 weeks	Baseline technical domains were not significantly different. Post-intervention p-values favored VR group for presentation *p* = 0.002, correctness *p* = 0.006, coherence *p* = 0.019, and rhythm *p* = 0.004; coordination was not significant *p* = 0.283.	The VR simulation system was reported to improve several technical performance domains, but limited reporting of allocation, participants, dose, scoring, and reliability requires cautious interpretation.
(Panfilova, 2025)	Age Group 1 female aerobic gymnasts aged 12–14 years versus Age Group 2 female aerobic gymnasts aged 15–17 years.	Simple Hand-Eye Response: 233.63 ± 20.36 ms in Age Group 1 and 224.43 ± 17.42 ms in Age Group 2; no significant difference reported. Distraction Tolerance: 352.64 ± 33.14 ms versus 328.42 ± 25.72 ms; no significant difference reported. Response to a Moving Object precise responses: 21.9 ± 3.79 versus 24.3 ± 4.03, *t* = −1.44, df = 17.9, *p* = 0.08, effect size = 0.68. Tapping interval: right hand 184 ± 22.9 versus 167 ± 20.5 ms, *p* = 0.06; left hand 211 ± 23.2 versus 186 ± 31.6 ms, *p* = 0.04. Right-hand intervals were shorter than left-hand intervals within both age groups, *p* = 0.01.	Aerobic gymnastics athletes showed good reaction time, high concentration, and ability to maintain rhythm. Older athletes tended toward more precise and faster movements, but evidence is limited by small sample and age/experience confounding.
(Mihaela and Dragomir, 2021)	Descriptive neuromuscular and physiological values during a three-set maximal vertical-jump protocol in junior aerobic gymnasts.	Jump counts: both feet 44.31, left foot 46.15, right foot 45.54. Contact times: 0.20 s, 0.345 s, 0.354 s. Jump heights: 33.71 cm, 12.71 cm, 12.28 cm. Power: 47.18 W, 15.01 W, 14.67 W. Maximum heart rate mean 184.92 beats/min, range 166–197; blood lactate mean 12.50 mmol/l, range 8.80–15.60.	The protocol provides objective monitoring information for lower-body neuromuscular behavior and physiological demands in aerobic gymnastics. The study supports feasibility for training planning but not intervention effects or measurement properties.
(Sang, 2023)	Functional training plus standard training versus standard training control over 12 weeks.	FMS total score improved from 9.22 to 15.65 in the experimental group and from 9.83 to 11.23 in the control group. For FMS subtests, shoulder flexibility, active lifting, body-control push-up, swivel stability, and total score had *p* < 0.05; squat, hurdle step, and straight lunge had *p* > 0.05. Special physical fitness tests showed significant post-intervention differences favoring functional training in 8 of 10 indicators; cross fork and 10 consecutive curls/split jumps were not significant.	Functional training was reported to improve movement quality and special physical fitness more than standard training alone. Interpretation remains cautious because the training protocol and outcome statistics are incompletely reported.
(Sun, 2022)	Pre- versus post-training changes after specialized core-strength training; control group mentioned but post-test comparator data are not fully reported.	Five reported indicators all *p* < 0.05: two-foot single-arm push-ups 14.96 ± 1.35 versus 10.867 ± 1.843, *t* = 5.467; acute angle and leg support 3.76 ± 0.83 versus 2.099 ± 0.67, *t* = 6.000; hanging leg lift 13.53 ± 0.703 versus 10.067 ± 1.58, t = 6.045; split-leg jump 13.06 ± 0.89 versus 10.199 ± 1.638, *t* = 6.703; standing high body forward bending 31.06 ± 2.282 versus 28.799 ± 3.52, *t* = 1.108.	The study concluded that core-strength training improves core competence and physical conditioning of female college competitive aerobics athletes.
(Tibenská and Medeková, 2014)	Individual Z-score profiles for anthropometric, body-composition, motor-test, and competition-performance indicators over two years.	Motor *Z*-scores ranged from 4.28 to −5.70; anthropometric *Z*-scores ranged from −8.72 to 7.96. Competition points/positions included L.S. 17.84/1st, F.N. 17.71/2nd, M.Z. 16.72/3rd, and F.M. 8.73/12th.	The study concludes that higher motor Z-scores combined with lower anthropometric Z-scores generally corresponded to higher competition performance, but one athlete showed discordance, supporting the need for multifactorial interpretation including psychological factors.
(Todorova et al., 2021)	Main author-model group versus control group across one annual macrocycle.	Harvard Step Test index improved from 79.32 ± 1.5 to 80.43 ± 1.2 in the main group (1.39%, *t* = 5.7, *p* < 0.001) and from 78.55 ± 1.7 to 79.39 ± 1.5 in control (1.08%, *t* = 4.2, *p* < 0.001). Integral technical preparedness changed by 11.5% in the main group and 8.8% in control.	The study concluded that the two-cycle annual training model improved special physical preparedness, technical quality/stability, and competition readiness.
(Trisanda et al., 2022)	Pre-test versus post-test after single-leg speed-hop exercise.	Combined standing broad jump: pre-test total 1,532, mean 191.50; post-test total 1575, mean 196.88; t_observed = 14.333, t_critical = 1.895, significance level 0.05. Male means 227.75 to 232.5; female means 155.25 to 161.25.	The study concluded that single-leg speed-hop exercise significantly improved leg muscle power in Riau aerobic gymnastics athletes.
(Viktorovich and Vladimirovna, 2019)	Psychophysiological status across three preparation stages in the Russian national sports-aerobics team preparing for the 2017 World Games.	Dynamic tremometry: women travel time 21,174 ms to 20,755 ms to 18,093 ms; touches 7.9 to 7.1 to 6.4. Men travel time 35,726 ms to 23516 ms to 20,759 ms; touches 6.2 to 6.3 to 7.1. Response-to-moving-object results: athletes shifting toward inhibition increased by 13% in women and 40% in men by the second stage. Zung scale: men 7 and 10 and woman 1 were borderline between normal values and mild depression at the first stage; men normalized by the third stage while woman 1 remained borderline.	Psychophysiological monitoring was reported as useful for managing training, identifying athletes needing psychological support, and creating model characteristics for highly skilled sports-aerobics athletes.
(Wang, 2015)	Fuzzy comprehensive evaluation of competitive-aerobics athlete body function.	Group fuzzy comprehensive evaluation vector [0, 0.01, 0.1175, 0.3475, 0.525] generated a score of 86.7, interpreted as good. Illustrative scores A1 = 0.22, A2 = 0.23, A3 = 0.19; text states A2 > A1 > A3 but conclusion notation appears inconsistent.	The model is reported as adaptable for body-function evaluation.
(Wu, 2023)	Pre-training versus post-training after special lower-limb strength training.	Core composite index before/after: A 27.06 to 26.01, B 27.90 to 27.17, C 27.43 to 26.74, D 26.00 to 27.30. Paired *t* = −16.417, *df* = 7, *p* < 0.001. Regression model: *r* = 0.977, adjusted *R*^2^ = 0.926, *F* = 119.342, *P* = 0.009 < 0.07. Handstand against wall 45° reportedly increased by 40 s and inversion control by 30–40 s.	The study concluded that special lower-limb strength training improved special physical quality and stability.
(You, 2023)	Twelve-week core-strength training versus traditional waist/abdominal training.	Post-test side bridge 21.2 ± 5.46 versus 16.6 ± 5.41, *t* = 2.675, *df* = 38, *p* = 0.011; sixth-grade up/supine bridge 30.2 ± 9.66 versus 21.2 ± 9.71, *t* = 2.971, *p* = 0.005; abdominal bridge 73.5 ± 21.77 versus 54.5 ± 21.08, *t* = 2.804, *p* = 0.008. Improvement rates 41.3%, 69.9%, and 58.1% versus 7.1%, 19.5%, and 17.8%.	Core-strength training improved core strength more than traditional training.
(Yu and Liu, 2024)	High-level versus average aerobic gymnasts performing C715.	Upright restoration 0.64 ± 0.03 s versus 0.16 ± 0.02 s, *p* < 0.05; pre-swing leg angle 46.52 ± 2.14° versus 36.12 ± 3.35°, *p* < 0.05; right gastrocnemius IEMG 25.77 ± 3.64 versus 8.77 ± 1.23 μV·S, *p* < 0.05; minimum right ankle angle 126.87 ± 3.21° versus 112.66 ± 2.67°, *p* = 0.021; upright max right ankle 128.68 ± 2.33° versus 110.23 ± 2.37°, *p* = 0.013.	High-level athletes showed better joint control and distinct activation.
(Yudho et al., 2024)	Active versus retired/former aerobic gymnasts during wearable-inclinometer stability tasks.	Bi-X U = 34134, *p* < 0.001; Bi-Y U = 11055, *p* < 0.001; Uni-X U = 34784, *p* = 0.002; Uni-Y U = 40359, *p* = 0.785; X-Tip U = 33188, *p* < 0.001; Y-Tip U = 38132, *p* = 0.162. Uni-X with Bi-X Kendall Tau B = 0.51, *p* < 0.001.	The inclinometer may be useful for stability mapping.
(Yusupava et al., 2021)	Exploratory factor structure of complex preparedness indicators in 20 young female aerobic gymnasts.	Six factors explained 100% of variance: speed-strength abilities 28.7%; coordination abilities 26.9%; strength qualities 20.8%; flexibility 14.6%; speed qualities 5.2%; special endurance 3.8%.	The study concluded that technical-element teaching in aerobic gymnastics should emphasize both spatiotemporal execution parameters and speed-power aspects of execution.
(Zhang, 2023)	Suspension rehabilitation training compared with traditional stretching recovery for lumbar movement and injured-ankle static-balance outcomes.	Lumbar amplitudes improved more in the suspension group than control; for example left-handed/left-rotation amplitude changed from 119.130 ± 2.447 to 161.070 ± 8.655 nm in the experimental group versus 122.713 ± 48.423 to 127.225 ± 43.203 nm in control. Injured ankle overall static parameter changed from 3.807 ± 0.734° to 2.725 ± 0.754° in the experimental group and from 3.807 ± 0.734° to 2.805 ± 0.736° in control.	The study concludes that suspension training can support joint injury control and recovery.
(Zhao et al., 2025)	Proposed deep learning plus Bayesian hierarchical ordered probit model compared with support vector machine, random forest, and non-hierarchical deep neural network baselines; risk predictors estimated using posterior coefficients.	Model performance: support vector machine accuracy 74.1%, macro-AUC 0.81, high-risk recall 76.5%; random forest 78.3%, 0.84, 80.0%; non-hierarchical deep neural network 81.0%, 0.87, 84.3%; proposed model 86.4%, 0.92, 91.2%, with macro-F1 0.85 and Brier score 0.087. Key posterior coefficients: peak vertical landing acceleration 0.42 (95% credible interval 0.28 to 0.56), peak ankle inversion angular velocity 0.37 (0.23 to 0.51), medial-lateral plantar pressure ratio 0.31 (0.18 to 0.44), random-effect standard deviation 0.63 (0.48 to 0.81).	The proposed model provided the best reported discrimination and calibration and identified landing-related variables as key risk indicators.

Twenty-one reports contributed change-related evidence. Twenty evaluated planned intervention, rehabilitation, or altered-exposure conditions, whereas one SAGAT development report compared performance across training-cycle phases. Among the 20 intervention or exposure reports, 14 reported predominantly favorable changes, 5 produced mixed, selective, or materially uncertain findings, and one prospective congested-training cohort reported adverse changes.

The SAGAT development report separately demonstrated change between preparatory and competition phases. This finding was classified as observed training-cycle change rather than formal responsiveness and was not included in the 20-report intervention or exposure denominator.

## 4 Discussion

The evidence base was broad in the number of constructs represented but uneven in depth. Strength-power and general or sport-specific physical-fitness assessments formed the largest cluster, followed by biomechanical and physiological monitoring. In contrast, aerobic gymnastics-specific evidence on validity, reliability, measurement error, external validation, and long-term responsiveness was limited for most tool families. Among the methods reviewed, the Specific Aerobic Gymnastics Anaerobic Test had the most complete preliminary aerobic gymnastics-specific measurement evidence. The available development study reported test–retest reliability, a minimal detectable change estimate, convergent associations with Wingate-derived anaerobic indices, and observed change across phases of a training cycle (Alves et al., 2015). However, this evidence was generated primarily within one research program using relatively small samples and should not be interpreted as broad validation across populations, laboratories, countries, or competitive contexts.

The review also shows that monitoring in aerobic gymnastics is intrinsically multidimensional. Competitive routines impose substantial aerobic and anaerobic demands, while successful performance also depends on strength, power, repeated-jump ability, balance, flexibility, motor control, and technically efficient management of take-off and landing loads (Aleksandraviciene et al., 2015; Mihaela and Dragomir, 2021; Kyselovičová and Zemková, 2024). No single test therefore captures the complete performance or readiness profile. The principal contribution of this review is to distinguish evidence that supports the measurement quality of a tool from evidence that merely demonstrates its use, physiological plausibility, or change after an intervention.

### 4.1 Measurement properties and the distinction between tool use and tool validation

Measurement-property evidence was the least developed component of the literature relative to the number of assessment methods used. The SAGAT currently has the most complete preliminary sport-specific evidence. Its completion time was associated with upper- and lower-body Wingate indices; test–retest reliability was reported; and a minimal detectable change estimate was calculated (Alves et al., 2015). Performance also differed between preparatory and competition phases. These findings indicate that the test is a promising discipline-specific measure of anaerobic performance and may be more closely aligned with aerobic-gymnastics movement demands than a generic cycle-ergometer assessment.

The findings should nevertheless be interpreted cautiously. The associations with Wingate outcomes represent convergent construct-validity evidence rather than definitive criterion validity because the two tests do not measure an identical construct using an accepted reference standard. Similarly, change across a training cycle indicates potential sensitivity to change but does not, by itself, establish formal responsiveness. The evidence was derived mainly from one development study with relatively small samples, and independent replication is lacking across male and female athletes, different age groups, competitive levels, countries, laboratories, and routine formats. The SAGAT should therefore be described as the method with the most complete preliminary aerobic-gymnastics-specific measurement evidence, rather than as a fully validated, reliable, and responsive test for the discipline as a whole.

Other measurement-property studies addressed narrower questions. The proposed flexibility test showed strong content-validity ratings and high reliability for averaged ratings, but its single-rater reliability was appreciably lower, and the development study relied on expert or rater assessment rather than empirical testing in the intended athlete population (Budiarti et al., 2022). The L-support scoring study reported high inter-rater reliability and demonstrated that execution scores were sensitive to an acute attentional-focus manipulation, supporting the reproducibility of judged movement form under the study conditions (Maria Laroëre et al., 2024). However, this does not establish longitudinal responsiveness or agreement across different judging panels and competitive contexts. Similarly, comparison of skinfold, ultrasound, and bioimpedance estimates identified method-dependent differences and agreement between only selected methods; without a criterion reference, the results do not identify a preferred body-composition method (Lopes et al., 2024).

Across the wider evidence base, many field batteries, force-platform variables, psychophysiological indices, and digital outputs were used as study outcomes without prior aerobic-gymnastics-specific estimates of reliability or measurement error. This creates an important interpretive asymmetry since studies frequently reported statistically significant pre–post differences but rarely established whether those differences exceeded expected biological or technical variation. The property-specific appraisal identified recurrent limitations in familiarization, assessor standardization, repeated-test conditions, missing-data reporting, and estimation of measurement error. Formal responsiveness was particularly uncommon because intervention-related change was often reported without prespecified change hypotheses, an external criterion, or comparison against a smallest detectable change. Future validation studies should therefore evaluate reliability, measurement error, construct or criterion validity, and responsiveness as separate properties rather than treating them as interchangeable.

### 4.2 Physiological and metabolic monitoring

The physiological evidence supports a mixed energetic interpretation of aerobic gymnastics. During competitive group routines, aerobic metabolism accounted for a substantial proportion of total energy demand, but anaerobic alactic and lactic contributions were also important (Aleksandraviciene et al., 2015). International- and national-level athletes did not differ clearly in peak oxygen uptake, whereas the international-level group showed a greater relative anaerobic contribution during more difficult routines. This pattern suggests that competitive level may be differentiated less by generic maximal aerobic capacity than by the ability to integrate high-intensity technical work, anaerobic contribution, and movement efficiency within the routine. Because the comparison was based on a small female sample and a specific group-routine context, this interpretation remains provisional.

Some studies provided convergent evidence that routine-like or repeated-jump tasks elicit high physiological strain. Heart-rate monitoring indicated that demanding elements performed later in routines were associated with very high heart rates, and a maximal repeated-jump protocol produced high heart-rate and blood-lactate responses in junior gymnasts (Bota and Urzeală, 2013; Mihaela and Dragomir, 2021). The lactate response observed during the SAGAT was also comparable with simulated competition and Wingate testing, strengthening the physiological relevance of that field test (Alves et al., 2015). These findings provide a physiological rationale for combining external performance measures with internal-load indicators, although the measurement properties and decision thresholds of such combinations remain insufficiently established in aerobic gymnastics.

Generic laboratory and field tests remain useful, but their role differs from that of sport-specific assessments. Incremental treadmill cardiopulmonary testing can characterize maximal oxygen uptake and threshold responses, whereas the 20-m multistage fitness test offers a more feasible field estimate of aerobic fitness. Longitudinal cardiopulmonary observations in three youth athletes showed heterogeneous trajectories during puberty, illustrating why age, maturation, and individual training history should be considered when interpreting repeated values (Chayun and Sharovarova, 2025). Cross-sectional comparisons with non-athletic controls also suggest that sustained sports-aerobics participation is associated with higher cardiorespiratory fitness and physical capacity, particularly in older age groups (Filippova et al., 2006). These studies establish physiological relevance, but they do not provide comprehensive sport-specific reference values or demonstrate that changes in generic aerobic tests translate directly into improved competitive execution.

### 4.3 Strength-power, functional preparedness, and observed change following training

Strength-power and physical-fitness testing represented the densest evidence cluster. Field batteries commonly included push-ups; supports; trunk flexion and extension tests; standing long jump; shuttle running; repeated jumps; and flexibility tasks, while laboratory assessments included countermovement jump, squat jump, isokinetic dynamometry, and Wingate testing. This concentration is consistent with the technical requirements of aerobic gymnastics, but the evidence indicates that different domains adapt at different rates. In a 2-year follow-up of one elite athlete, dynamic balance, postural coordination, repeated-jump endurance, and selected isokinetic variables improved, whereas vertical explosive power and Wingate performance remained largely unchanged (Kyselovičová and Zemková, 2024). The finding is not generalizable from a single athlete, but it illustrates why a multidimensional battery may detect adaptation that would be missed by a single global score.

The most developed interval-training evidence arose from two related randomized reports from the same research program in youth female regional-level aerobic gymnasts. One compared jumping interval training with running high-intensity interval training and usual training (Ma et al., 2024a), whereas the other compared once- and twice-weekly jumping interval training with usual training (Ma et al., 2024b). These studies provide a comparatively coherent example of outcome measures that changed in the expected direction following a targeted training stimulus. They do not, however, establish that each measure is formally responsive or suitable for routine monitoring across other populations and contexts.

A broader group of studies reported improvements after core-strength, functional, plyometric, elastic-resistance, and special physical-preparation programs (Artemieva et al., 2019; Hao et al., 2022; Kokarev et al., 2023a, 2023b; Sang, 2023; You, 2023). Although the direction of findings was generally favorable, the methodological appraisal showed that quasi-experimental evidence was dominated by high concern. Small samples, unclear allocation, incompletely described comparators, limited blinding, and sparse reporting of adherence or intervention fidelity reduce confidence that observed changes can be attributed to the experimental program. Heterogeneous test batteries and scoring systems further limit comparison across studies. Thus, the literature supports the feasibility of monitoring multiple aspects of functional preparedness, but it does not yet support a standardized hierarchy of tests or definitive conclusions about the relative effectiveness of competing training approaches.

Monitoring during congested training provides evidence of exposure-related change rather than formal responsiveness. The decreases in jump power and adverse changes in landing-force outcomes demonstrate that the measures varied during a period of increased training frequency (Ma et al., 2024c). These changes are consistent with acute neuromuscular fatigue or non-functional overreaching, but the study did not establish prospective links with injury or competitive performance. However, the study did not determine whether the instruments validly detected the intended construct of fatigue, whether the changes exceeded measurement error, or whether prespecified change thresholds predicted injury or performance. The results suggest that repeated force-platform testing may be informative during congested training when assessment conditions and recovery intervals are standardized. Replication and determination of measurement-error and decision thresholds are required before these measures can be used to classify fatigue or overreaching.

### 4.4 Biomechanical, technical, and landing-load monitoring

Biomechanical studies expanded the monitoring framework beyond capacity tests by examining how athletes execute sport-specific skills. Motion capture, force platforms, surface electromyography, inertial sensors, and calibrated video identified differences in jump preparation, joint control, muscle activation, symmetry, and landing load (Bota et al., 2014; D’Anna et al., 2019; Lamošová et al., 2020; Yu and Liu, 2024; Ge et al., 2025). For example, a pre-jump technique incorporating a flight phase was associated with greater jump height and flight time in a small senior sample, while high- and lower-level athletes differed in joint loading and muscle activation during rotational landings and complex difficulty elements (D’Anna et al., 2019; Yu and Liu, 2024; Ge et al., 2025). These findings show how laboratory measures can provide mechanistic information that is not available from a final execution score. These methods provide mechanistic and coaching-relevant information, but physiological or biomechanical plausibility should not be interpreted as evidence of measurement reliability, diagnostic validity, or prospective injury-prediction utility.

The principal limitation is that most biomechanical evidence was descriptive, cross-sectional, or based on case studies. Technical deviations identified in one athlete or a small elite group may be useful for individualized coaching, but they cannot be assumed to represent population-level risk factors. Similarly, the force-platform proposal for upper-body explosive and landing tasks was demonstrated in only two athletes and explicitly requires larger-sample validation (Albano et al., 2021). Protocols also differed in movement selection, equipment, filtering, outcome definitions, and trial selection, restricting direct comparison and preventing the establishment of reference ranges.

The injury-related evidence remains especially preliminary. Anthropometric and alignment measures were associated with season injury status in one cohort, but the resulting model had limited sensitivity and requires independent replication (Abalo-Núñez et al., 2018). A later biomechanical prediction model reported strong internal discrimination and identified landing acceleration, ankle-inversion angular velocity, and plantar-pressure distribution as important predictors, but it was appraised as high concern because validation was limited and the sample was small (Zhao et al., 2025). Prospective surveillance with prespecified outcomes, adequate event numbers, external validation, and calibration assessment is needed before injury-risk tools can support individual clinical or training decisions.

To avoid conflating measurement validation, physiological relevance, intervention-related change, and technological novelty, **Table 6** presents a provisional classification of the assessment methods according to the strongest type of evidence currently available.

**Table 6:** Provisional practical interpretation of assessment and monitoring methods in aerobic gymnastics according to the current evidence base.

Evidence category	Tool or tool family	Current evidence in aerobic gymnastics	Principal limitations	Provisional practical interpretation	Representative reports
Formal responsiveness evidence	All included tools	No report met the complete criteria for formal responsiveness.	No study combined an explicit responsiveness design with prespecified change hypotheses, an appropriate comparator or external anchor, and adequate interpretation against measurement error	No included method should currently be described as formally responsive in aerobic gymnastics	Not applicable
Preliminary sport-specific measurement-property support	Specific Aerobic Gymnastics Anaerobic Test (SAGAT)	Test–retest reliability, minimal detectable change, convergent associations with Wingate-derived anaerobic indices, and observed change across training phases.	Evidence derives mainly from one development program with relatively small samples. Independent replication is lacking across sexes, ages, competitive levels, countries, laboratories, and routine formats. Criterion validity and formal responsiveness remain incompletely established.	Currently the leading candidate for sport-specific anaerobic assessment, but not a universally validated standard. Use the published protocol, conduct local familiarization, and interpret change cautiously rather than applying universal thresholds.	Alves et al. (2015)
Preliminary sport-specific measurement-property support	Aerobic-gymnastics flexibility test	Expert-based content-validity evidence and rater-reliability estimates for a discipline-specific flexibility assessment.	Development relied on expert ratings rather than testing in the intended athlete population. Athlete-level test–retest reliability, measurement error, validity, and responsiveness were not established.	Developmental instrument only. It may help structure flexibility assessment, but it should not yet be used as a validated monitoring tool or to define athlete thresholds.	Budiarti et al. (2022)
Preliminary sport-specific measurement-property support	L-support technical scoring	Inter-rater reliability was reported for judged execution during a controlled attentional-focus experiment.	Evidence concerns one skill and one experimental context. Test–retest reliability, agreement across judging panels, longitudinal responsiveness, and relation to competition outcomes remain unknown.	May support standardized technical scoring in similar controlled conditions, but evidence is insufficient for routine longitudinal monitoring or generalized judging interpretation.	Maria Laroëre et al. (2024)
Preliminary measurement-comparison evidence	Skinfold, ultrasound, and bioimpedance body-composition assessment	Method-comparison and agreement evidence was reported among three commonly used body-composition approaches.	No accepted criterion reference was used, and the methods were not shown to be interchangeable. Aerobic-gymnastics-specific error thresholds and responsiveness were not established.	Select one feasible method and retain the same protocol for repeated assessment. Avoid comparing values obtained from different methods or treating one method as definitively superior.	Lopes et al. (2024)
Physiological or biomechanical relevance without sufficient measurement validation	Heart rate, blood lactate, gas exchange, treadmill testing, 20-m multistage testing, force platforms, motion capture, electromyography, isokinetic testing, jump testing, and balance assessment	The studies demonstrate physiological, energetic, neuromuscular, balance, technical, or mechanical relevance to aerobic-gymnastics routines and skills.	Evidence is commonly cross-sectional, descriptive, case-based, or based on small selected samples. Aerobic-gymnastics-specific reliability, measurement error, reference values, and decision thresholds are generally absent.	Use only for a clearly defined question and under standardized conditions. These methods can characterize capacity or loading, but current evidence does not support universal cutoffs or a validated discipline-wide battery.	Aleksandraviciene et al. (2015); Bota et al. (2014); D’Anna et al. (2019); Ge et al. (2025); Kyselovičová et al. (2023); Yu and Liu (2024)
Observed change after training or exposure, without formal responsiveness	Countermovement and squat jumps, repeated-jump tests, SAGAT, 20-m multistage test, field-fitness batteries, Y-Balance, isokinetic strength, Functional Movement Screen, force-platform outcomes, and psychophysiological indices	Several studies reported pre–post or longitudinal change after interval, strength, plyometric, functional, special-preparation, annual-cycle, or congested-training exposure.	Many studies were small or quasi-experimental, and change was often inferred from statistical significance without comparison against measurement error or an external criterion. Links with competition performance, injury, or athlete-important outcomes were sparse.	These measures may be used provisionally for within-athlete tracking when the protocol is stable. An observed change should not automatically be interpreted as meaningful adaptation, fatigue, or readiness unless it exceeds known error and is corroborated by training and clinical context.	Artemieva et al. (2019); Kyselovičová and Zemková (2024); Ma et al. (2024a); Ma et al. (2024b); Ma et al. (2024c)
Observed load-related change, without validated decision thresholds	Session rating of perceived exertion and repeated force-platform monitoring during congested training	Repeated measures changed alongside increased training frequency and were compatible with greater internal load and reduced neuromuscular performance.	Evidence is limited to a small number of studies, and prospective thresholds for overreaching, injury, availability, or competition performance have not been established.	Potentially useful as part of an individualized monitoring process, but values should be interpreted as contextual signals rather than diagnostic or prescriptive thresholds.	Ma et al. (2024c)
Exploratory or investigational methods	Wearable sensors, artificial intelligence and machine learning models, virtual reality systems, digital decision-support tools, injury-prediction models, and multidimensional factor or composite-readiness models	Initial studies reported promising classification, prediction, feedback, or intervention results.	Development samples were generally small; overfitting, incomplete calibration, internal validation only, unstable multivariable structures, and absence of independent external validation were common.	Restrict to research, development, or adjunctive feedback. Do not use for individual risk prediction or automated decision making until externally validated and prospectively shown to improve decisions or athlete outcomes.	Chen (2025); Kozina et al. (2017); Li (2025); Luo et al. (2025); Niu (2022); Yudho et al. (2024); Yusupava et al. (2021); Zhao et al. (2025)

### 4.5 Balance, flexibility, anthropometry, and multidimensional athlete profiling

Balance and flexibility were commonly included in applied testing but had limited standalone validation. In elite adolescent gymnasts, dynamic balance differed among gymnastics disciplines and was correlated with dominant-leg knee-extension strength within the aerobic-gymnastics subgroup, indicating that balance performance may partly reflect strength capacity at different movement velocities (Kyselovičová et al., 2023). Longitudinal changes in Y-Balance and postural-coordination measures were also observed in an elite athlete (Kyselovičová and Zemková, 2024). Flexibility scores showed little observed change when baseline values were already high, as illustrated by the absence of substantial improvement despite changes in other physical qualities (Artemieva et al., 2019). This pattern may reflect a ceiling effect, limited intervention effect, measurement insensitivity, or normal variation.

Anthropometric and body-composition monitoring appeared relevant mainly as contextual information rather than as isolated indicators of readiness. Body-segment ratios were associated with L-sit performance in young athletes, supporting consideration of morphology when interpreting technical tests and prescribing progressions (Ayziaatullova et al., 2022). Over longer follow-up, anthropometric and motor-performance profiles showed only an imperfect correspondence with competition ranking, reinforcing the multifactorial nature of performance (Tibenská and Medeková, 2014). Method-dependent differences in body-fat estimates further indicate that repeated monitoring should use the same method and protocol rather than comparing values interchangeably across skinfolds, ultrasound, and bioimpedance (Lopes et al., 2024).

Multidimensional readiness models attempted to integrate autonomic, psychophysiological, physical, technical, and anthropometric indicators. Factor-analytic studies identified clusters related to autonomic regulation, nervous-system mobility, strength, timing, speed-strength, coordination, and flexibility (Kozina et al., 2017; Yusupava et al., 2021). These approaches are conceptually aligned with the multifactorial demands of the sport, but their statistical stability is uncertain because the samples were small relative to the number of variables and the derived structures were not replicated. Composite scores and factor models should therefore complement, rather than replace, transparent reporting of individual measures and their measurement properties.

### 4.6 Psychophysiological, perceptual, and attentional monitoring

Psychophysiological and perceptual studies suggest that readiness in aerobic gymnastics may include reaction speed, nervous-system mobility, inhibitory control, tremor, mood, and visual attention. Repeated national-team monitoring identified changes across preparation stages and highlighted individual athletes who might require psychological support (Viktorovich and Vladimirovna, 2019). Cross-sectional age comparisons generally showed faster responses and greater functional mobility in older athletes, although age, maturation, and accumulated training experience could not be separated (Chayun, 2024; Panfilova, 2025). Aerobic gymnasts also demonstrated an advantage over non-trained controls in divided attention but not under all selective-attention conditions, indicating that perceptual advantages may be task-specific rather than global (Guo et al., 2026).

### 4.7 Digital systems, wearable sensors, and decision-support models

Digital monitoring was an emerging but methodologically fragile evidence cluster. Studies evaluated accelerometer-based energy-expenditure prediction, wearable inclinometers, inertial-sensor feedback, virtual-reality training, artificial-intelligence decision systems, and machine learning models for landing quality or injury risk (Niu, 2022; Yudho et al., 2024; Chen, 2025; Li, 2025; Luo et al., 2025; Zhao et al., 2025). These technologies address important practical needs, including real-time feedback, automated movement classification, continuous load capture, and integration of multiple data streams. Internal results were often promising; for example, waist-worn accelerometry showed associations with metabolic measures, and sensor-based training systems reported high classification performance and improvements in force-related outcomes (Chen, 2025; Luo et al., 2025).

However, all prediction-model and digital decision-support studies were appraised as high methodological concern. Common limitations included small or inconsistently described samples, internal validation only, unclear handling of overfitting, incomplete comparator descriptions, and limited reporting of calibration, missing data, and model updating. Performance estimates obtained from a development dataset are likely to be optimistic when applied to new athletes, clubs, devices, or routines. Moreover, an algorithm can classify movement or predict an outcome accurately without demonstrating that its feedback improves coaching decisions or athlete outcomes. External validation, prospective impact studies, transparent feature definitions, calibration analysis, and comparison against simpler baselines are therefore necessary before these systems move from exploratory research to routine monitoring.

### 4.8 Limitations of the evidence base and research priorities

The deliberately broad population criterion enabled the review to map evidence across the full aerobic-gymnastics development pathway, but it also represents a major limitation to practical interpretation. The included populations differed substantially in age, biological and training maturity, sex, competition experience, event category, training volume, and competitive level. A result obtained in a young athlete at an initial preparation stage cannot be assumed to have the same normative meaning, measurement error, sensitivity, or decision threshold in an adult international competitor. Similarly, findings from predominantly female samples cannot automatically be generalized to male athletes or mixed-pair contexts.

The evidence base was concentrated in youth or junior athletes and female samples, while adult non-elite athletes, male athletes, mixed-pair competitors, and several event categories were sparsely represented. Competitive caliber was also described inconsistently as regional, national, international, high-level, qualified, highly skilled, or elite. Consequently, the review maps where evidence exists but cannot establish population-invariant reference values, universal thresholds, or a single monitoring battery applicable across the discipline.

The review also has limitations related to information-source coverage. Searches were restricted to PubMed, Scopus, and the Web of Science Core Collection because the authors did not have access to SPORTDiscus, Embase, CINAHL, CNKI, Wanfang, or other regional bibliographic databases. Although the three searched databases provided multidisciplinary coverage, and backward citation searching was undertaken for all included studies and relevant reviews, these procedures cannot guarantee complete retrieval. Citation searching may identify connected studies that are poorly indexed or use inconsistent terminology, but it is less likely to identify isolated reports that are absent from the selected databases and are not cited by the retrieved literature.

This limitation may be particularly relevant because several included studies originated from China, Russia, Ukraine, and other non-English-speaking contexts. No language restriction was applied at eligibility, but the use of primarily English search terminology and the absence of regional-database searching may have reduced the retrieval of reports available only in local-language journals or repositories. In addition, no systematic search of trial registries, thesis repositories, preprint servers, conference archives, or other dedicated grey-literature sources was conducted. Consequently, regional-language, unpublished, null, or preliminary evidence may be underrepresented, and the evidence map should be interpreted as a map of the literature identified through the specified sources rather than a guarantee of exhaustive global coverage.

Important conceptual gaps were also evident. Measurement error and smallest detectable change were rarely reported, minimal important change was essentially absent, and responsiveness was often inferred from statistical significance rather than evaluated as a measurement property. Few studies connected monitoring changes to judged competition performance, prospective injury, availability for training, or other athlete-important outcomes. Long-term training-load interpretation was limited, and there was no sufficiently validated core monitoring battery or outcome set. External validation was particularly sparse for digital systems and prediction models, while evidence on feasibility, cost, athlete burden, and implementation across resource settings was inconsistently reported.

Future research should prioritize multicenter measurement-property studies using standardized protocols and adequately sized, sex- and age-diverse samples. For established field tests, investigators should report familiarization, test–retest reliability, measurement error, smallest detectable change, and longitudinal responsiveness. For laboratory and wearable measures, protocol harmonization should include equipment specifications, sampling and filtering procedures, trial-selection rules, and normalization methods. Training studies should prespecify primary outcomes, document adherence and co-interventions, and distinguish between changes in test performance and changes in competition execution. Injury-related studies require prospective exposure-based surveillance, sufficient injury events, external model validation, and evaluation of whether monitoring-informed actions improve outcomes. A consensus process involving athletes, coaches, clinicians, judges, and sport scientists could then develop a core monitoring framework that balances measurement quality, sport specificity, feasibility, and interpretability.

### 4.9 Practical implications

The practical implications of this review should be interpreted as a provisional framework rather than as recommendations for a validated monitoring battery. Tool selection should begin with the question the practitioner intends to answer, for example, whether the purpose is to characterize aerobic or anaerobic capacity, examine landing mechanics, track within-athlete change, or investigate fatigue during a congested training period. Evidence that a method is physiologically plausible, technically relevant, or responsive to an intervention does not necessarily establish that it is reliable enough to guide individual decisions.

**Table 6** separates methods according to the strongest type of evidence currently available. The SAGAT has the most complete preliminary aerobic-gymnastics-specific measurement evidence, but its support is derived mainly from one development study and requires independent replication. Other specific assessments, including flexibility and technical-scoring procedures, have partial content-validity or rater-reliability evidence but have not been validated comprehensively in their intended athlete populations.

Heart rate, blood lactate, gas exchange, jump, strength, balance, force-platform, motion-capture, electromyographic, and kinematic methods have physiological or biomechanical relevance to aerobic gymnastics. They may help answer targeted performance or loading questions, but most lack discipline-specific reliability, measurement-error estimates, reference values, or decision thresholds. Their use should therefore be restricted to populations and protocols comparable to those studied, and results should be interpreted descriptively rather than against universal cutoffs.

Several field and laboratory measures changed after training or altered exposure, including the SAGAT, countermovement and squat jumps, repeated-jump tests, the 20-m multistage fitness test, balance measures, isokinetic strength, field-fitness batteries, and force-platform outcomes. Such change supports their potential use in longitudinal assessment but should not automatically be labelled responsiveness. For an observed change to guide practice, it should exceed expected measurement error, occur under standardized testing conditions, and be considered alongside training exposure, symptoms, technical execution, and competition context.

Wearable sensors, machine learning algorithms, artificial intelligence systems, virtual reality applications, injury-prediction models, and multidimensional readiness models remain exploratory. Their internal results are potentially useful for research and technology development, but external validation, calibration assessment, model updating, and prospective impact studies are required before they can support automated or individual-level decisions.

Accordingly, the review does not support a validated core battery for aerobic gymnastics. A provisional assessment program may combine coaching and judging information with selected physiological, neuromuscular, or biomechanical measures, but the choice of measures should remain decision-specific, population-specific, and constrained by the measurement evidence available for the intended use.

## 5 Conclusions

Evidence in aerobic gymnastics is concentrated in strength, power, physical-fitness assessment, physiological characterization, and biomechanical description. In contrast, formal measurement properties research, long-term responsiveness, external validation of digital tools, and prospective injury-related validity remain limited. Many methods have therefore been used more often than they have been validated. The SAGAT currently has the most complete preliminary aerobic-gymnastics-specific measurement evidence, including test–retest reliability, a minimal detectable change estimate, convergent associations with anaerobic-performance measures, and observed change across a training cycle. However, this evidence derives mainly from one development study with relatively small samples, and independent replication is required across sexes, ages, competitive levels, countries, laboratories, and routine formats. It should not yet be described as a universally reliable, valid, and responsive test. The current literature supports a provisional, multidimensional, and decision-specific approach to assessment rather than a validated core monitoring battery. Physiological relevance, biomechanical plausibility, and intervention-related change should be distinguished from formal evidence of reliability, validity, measurement error, and responsiveness. Future work should prioritize standardized protocols, independent measurement-property studies, longitudinal cohorts, and prospective links with competition performance, training availability, fatigue, and injury.

## Data Availability

The original contributions presented in the study are included in the article/**Supplementary Material**. Further inquiries can be directed to the corresponding author.
