# Supplementary material for: Field-based and laboratory monitoring tools in aerobic gymnastics: a scoping review of measurement properties, physiological relevance, and change following training or competitive exposure
Source: Front Physiol. 2026 Sep 8;17:1920787. doi: 10.3389/fphys.2026.1920787 (PMC13593834; doi:10.3389/fphys.2026.1920787)
Supplement: Supplementary file 1 [file Table1.docx]

# Supplementary materials

**Supplementary Table S1. Appraisal framework assigned to each included study**

| Study ID | Citation | Appraisal framework(s) |
| --- | --- | --- |
| AGMON-001 | Abalo-Núñez et al. 2018 | JBI cohort |
| AGMON-002 | Albano et al. 2021 | JBI case series |
| AGMON-003 | Aleksandraviciene et al. 2015 | JBI analytical cross-sectional |
| AGMON-004 | Alves et al. 2015 | Adapted COSMIN-informed measurement-property appraisal |
| AGMON-005 | Lamošová et al. 2020 | JBI case report |
| AGMON-006 | Artemieva et al. 2019 | JBI quasi-experimental |
| AGMON-007 | Ayziaatullova et al. 2022 | JBI analytical cross-sectional |
| AGMON-008 | Bota and Urzeală 2013 | JBI quasi-experimental |
| AGMON-009 | Bota et al. 2014 | JBI case series |
| AGMON-010 | Budiarti et al. 2022 | Adapted COSMIN-informed measurement-property appraisal |
| AGMON-011 | Chayun and Manzheley 2019 | JBI case series |
| AGMON-012 | Chayun 2024 | JBI analytical cross-sectional |
| AGMON-013 | Chayun and Sharovarova 2025 | JBI case series |
| AGMON-014 | Chen 2025 | PROBAST (2019) |
| AGMON-015 | D’Anna et al. 2019 | JBI analytical cross-sectional |
| AGMON-016 | Ding 2023 | JBI quasi-experimental |
| AGMON-017 | Filippova et al. 2006 | JBI analytical cross-sectional |
| AGMON-018 | Ge et al. 2025 | JBI analytical cross-sectional |
| AGMON-019 | Guo et al. 2026 | JBI analytical cross-sectional |
| AGMON-020 | Hao et al. 2022 | JBI quasi-experimental |
| AGMON-021 | Kokarev et al. 2023 | JBI quasi-experimental |
| AGMON-022 | Kokarev et al. 2023b | JBI quasi-experimental |
| AGMON-023 | Kozina et al. 2017 | JBI analytical cross-sectional |
| AGMON-024 | Kyselovičová et al. 2023 | JBI analytical cross-sectional |
| AGMON-025 | Kyselovičová and Zemková 2024 | JBI case report |
| AGMON-026 | Lamošová et al. 2021 | JBI quasi-experimental |
| AGMON-027 | Laroëre et al. 2024 | JBI quasi-experimental |
| AGMON-028 | Li 2025 | PROBAST (2019) |
| AGMON-029 | Lopes et al. 2024 | Adapted COSMIN-informed measurement-property appraisal |
| AGMON-030 | Luo et al. 2025 | RoB 2 for intervention effect; PROBAST (2019) for algorithmic component |
| AGMON-031 | Ma et al. 2024 (JSSM) | RoB 2 |
| AGMON-032 | Ma et al. 2024 (Heliyon) | JBI cohort |
| AGMON-033 | Ma et al. 2025 | RoB 2 |
| AGMON-034 | Mehrtash et al. 2015 | JBI quasi-experimental |
| AGMON-035 | Niu 2022 | JBI quasi-experimental |
| AGMON-036 | Panfilova 2025 | JBI analytical cross-sectional |
| AGMON-037 | Puiu and Dragomir 2021 | JBI analytical cross-sectional |
| AGMON-038 | Sang 2023 | JBI quasi-experimental |
| AGMON-039 | Sun 2022 | JBI quasi-experimental |
| AGMON-040 | Tibenská and Medeková 2014 | JBI cohort |
| AGMON-041 | Todorova et al. 2021 | JBI quasi-experimental |
| AGMON-042 | Trisanda et al. 2022 | JBI quasi-experimental |
| AGMON-043 | Wang 2015 | JBI analytical cross-sectional |
| AGMON-044 | Wu 2023 | JBI quasi-experimental |
| AGMON-045 | You 2023 | JBI quasi-experimental |
| AGMON-046 | Yu and Liu 2024 | JBI analytical cross-sectional |
| AGMON-047 | Yudho et al. 2024 | Adapted COSMIN-informed measurement-property appraisal |
| AGMON-048 | Yusupava et al. 2021 | JBI analytical cross-sectional |
| AGMON-049 | Zhang 2023 | JBI quasi-experimental |
| AGMON-050 | Zhao et al. 2025 | PROBAST (2019) |

Note. The 50 studies generated 51 appraisal units because Luo et al. (2025) was appraised with both RoB 2 and PROBAST.

**Supplementary Table S2A. JBI Checklist for Cohort Studies: final item-level judgments**

| **Item** | **Operational wording** |
| --- | --- |
| Q1 | Were the groups similar and recruited from the same population? |
| Q2 | Were exposures measured similarly in the groups? |
| Q3 | Was the exposure measured validly and reliably? |
| Q4 | Were confounding factors identified? |
| Q5 | Were strategies to address confounding stated? |
| Q6 | Were participants free of the outcome at baseline? |
| Q7 | Were outcomes measured validly and reliably? |
| Q8 | Was follow-up time sufficient? |
| Q9 | Was follow-up complete or adequately described? |
| Q10 | Were strategies used to address incomplete follow-up? |
| Q11 | Was the statistical analysis appropriate? |

| **Study** | **Q1** | **Q2** | **Q3** | **Q4** | **Q5** | **Q6** | **Q7** | **Q8** | **Q9** | **Q10** | **Q11** |
| --- | --- | --- | --- | --- | --- | --- | --- | --- | --- | --- | --- |
| Abalo-Núñez et al. 2018 | U | U | U | U | U | U | U | Y | U | U | U |
| Ma et al. 2024 (Heliyon) | U | Y | U | U | U | Y | U | Y | U | U | U |
| Tibenská and Medeková 2014 | U | Y | U | U | U | Y | U | Y | U | U | U |

**Note.** Y, Yes; N, No; U, Unclear; NA, Not applicable. Intermediate or mixed archival ratings were conservatively standardized as U. JBI items were not summed and no global concern category was generated.

**Supplementary Table S2A-R. Principal rationale for each study**

| **Study** | **Evidence unit** | **Principal rationale** |
| --- | --- | --- |
| Abalo-Núñez et al. 2018 | Aerobic-gymnastics cohort n=51 with anthropometric predictors and season injury outcome. | Separable eligible cohort and relevant injury-prediction outcome; confounding, missing data and small-event modelling concerns. |
| Ma et al. 2024 (Heliyon) | Four-week congested-training monitoring cohort in 50 young female aerobic gymnasts. | Non-random exposure allocation and residual confounding; objective repeated monitoring. |
| Tibenská and Medeková 2014 | Two-year Z-score anthropometric and motor monitoring in junior female aerobic gymnasts. | Small descriptive cohort; limited comparator, confounding and reliability reporting. |

**Supplementary Table S2B. JBI Checklist for Case Series: final item-level judgments**

| **Item** | **Operational wording** |
| --- | --- |
| Q1 | Were inclusion criteria clearly defined? |
| Q2 | Was the condition or performance state measured in a standard, reliable way? |
| Q3 | Were valid methods used to identify the cases? |
| Q4 | Was there consecutive inclusion? |
| Q5 | Was there complete inclusion? |
| Q6 | Were participant demographics reported clearly? |
| Q7 | Was clinical or performance information reported clearly? |
| Q8 | Were outcomes or follow-up reported clearly? |
| Q9 | Was site or setting information reported clearly? |
| Q10 | Was the statistical analysis appropriate? |

| **Study** | **Q1** | **Q2** | **Q3** | **Q4** | **Q5** | **Q6** | **Q7** | **Q8** | **Q9** | **Q10** |
| --- | --- | --- | --- | --- | --- | --- | --- | --- | --- | --- |
| Albano 2021 | U | U | Y | U | U | U | Y | U | U | U |
| Bota 2014 | U | U | Y | U | U | U | Y | U | U | U |
| Chayun 2019 | U | U | Y | U | U | U | Y | U | U | U |
| Chayun 2025 | U | U | Y | U | U | U | Y | Y | U | U |

**Note.** Y, Yes; N, No; U, Unclear; NA, Not applicable. Intermediate or mixed archival ratings were conservatively standardized as U. JBI items were not summed and no global concern category was generated.

**Supplementary Table S2B-R. Principal rationale for each study**

| **Study** | **Evidence unit** | **Principal rationale** |
| --- | --- | --- |
| Albano 2021 | PUJT, EPUT and FFT force-platform tests in two aerobic gymnasts. | n=2 pilot; no reliability/validity analysis. |
| Bota 2014 | Xsens MVN motion-capture kinematic analysis of Group C jump elements in eight senior aerobic gymnasts. | Pilot case series; sampling and reliability unclear. |
| Chayun 2019 | UPFT-1/30 psychophysiological monitoring in sports-aerobics national-team athletes. | Limited numerical reporting and changing test batteries. |
| Chayun 2025 | Morphofunctional and CPET/treadmill monitoring in 3 female aerobic gymnasts. | n=3; no comparator; maturation confounding. |

**Supplementary Table S2C. JBI Checklist for Case Reports: final item-level judgments**

| **Item** | **Operational wording** |
| --- | --- |
| Q1 | Were demographic characteristics clearly described? |
| Q2 | Was athlete history or the context timeline clearly described? |
| Q3 | Was the current performance or clinical context clearly described? |
| Q4 | Were assessment methods and results clearly described? |
| Q5 | Was the intervention or procedure clearly described? |
| Q6 | Was post-assessment or follow-up clearly described? |
| Q7 | Were adverse events or harms identified and described? |
| Q8 | Were takeaway lessons provided? |

| **Study** | **Q1** | **Q2** | **Q3** | **Q4** | **Q5** | **Q6** | **Q7** | **Q8** |
| --- | --- | --- | --- | --- | --- | --- | --- | --- |
| Lamošová et al. 2020 | U | U | Y | U | NA | U | U | Y |
| Kyselovičová and Zemková 2024 | U | U | Y | Y | U | Y | U | Y |

**Note.** Y, Yes; N, No; U, Unclear; NA, Not applicable. Intermediate or mixed archival ratings were conservatively standardized as U. JBI items were not summed and no global concern category was generated.

**Supplementary Table S2C-R. Principal rationale for each study**

| **Study** | **Evidence unit** | **Principal rationale** |
| --- | --- | --- |
| Lamošová et al. 2020 | SIMI Motion 3D analysis of FIVS and FIFVS in one senior female Slovak national-team aerobic gymnast. | Single case; no reliability/repeatability; no comparator. |
| Kyselovičová and Zemková 2024 | 24-month monitoring of one elite aerobic gymnast using body composition, balance, jump, isokinetic, Wingate and lactate tests. | Single athlete with objective repeated tests, but no comparator and limited causal inference. |

**Supplementary Table S2D. JBI Checklist for Quasi-Experimental Studies: final item-level judgments**

| **Item** | **Operational wording** |
| --- | --- |
| Q1 | Was it clear what caused the effect? |
| Q2 | Were participants in compared groups similar? |
| Q3 | Did groups receive similar care apart from the intervention? |
| Q4 | Was there a control group? |
| Q5 | Were there multiple outcome measurements before and after the intervention? |
| Q6 | Was follow-up complete or adequately described? |
| Q7 | Were outcomes measured in the same way across groups or time points? |
| Q8 | Were outcomes measured reliably? |
| Q9 | Was the statistical analysis appropriate? |

| **Study** | **Q1** | **Q2** | **Q3** | **Q4** | **Q5** | **Q6** | **Q7** | **Q8** | **Q9** |
| --- | --- | --- | --- | --- | --- | --- | --- | --- | --- |
| Ding 2023 | U | U | U | Y | U | U | U | U | U |
| Hao 2022 | U | U | U | Y | U | U | U | U | U |
| Kokarev 2023 | U | U | U | U | U | U | U | U | U |
| Kokarev 2023b | U | U | U | U | U | U | U | U | U |
| Lamošová 2021 | U | U | U | Y | U | U | U | U | U |
| Laroëre et al. 2024 | U | U | U | U | U | U | U | U | U |
| Mehrtash 2015 | U | U | U | Y | U | U | U | U | U |
| Niu 2022 | U | U | U | Y | U | U | U | U | U |
| Sang 2023 | U | U | U | Y | U | U | U | U | U |
| Sun 2022 | U | U | U | Y | U | U | U | U | U |
| Todorova 2021 | U | U | U | Y | U | U | U | U | U |
| Trisanda 2022 | U | U | U | Y | U | U | U | U | U |
| Wu 2023 | U | U | U | Y | U | U | U | U | U |
| You 2023 | U | U | U | Y | U | U | U | U | U |
| Zhang 2023 | U | U | U | U | U | U | U | U | U |
| Artemieva 2019 | U | U | U | Y | U | U | U | U | U |
| Bota 2013 | U | U | U | N | U | U | U | U | U |

**Note.** Y, Yes; N, No; U, Unclear; NA, Not applicable. Intermediate or mixed archival ratings were conservatively standardized as U. JBI items were not summed and no global concern category was generated.

**Supplementary Table S2D-R. Principal rationale for each study**

| **Study** | **Evidence unit** | **Principal rationale** |
| --- | --- | --- |
| Ding 2023 | Elastic-band training intervention with jump/strength monitoring outcomes. | Poor allocation reporting, sparse intervention/control detail and internal sample-size inconsistency. |
| Hao 2022 | Core-strength training intervention with stability/core-strength monitoring. | Allocation, adherence, attrition, blinding, reliability and pre/post statistics insufficiently reported. |
| Kokarev 2023 | Innovative fitness methods for special physical fitness. | Allocation, baseline comparability and reliability/fidelity reporting limited. |
| Kokarev 2023b | Innovative fitness methods for technical fitness. | Likely companion sample/context; limited allocation and measurement reporting. |
| Lamošová 2021 | One-year anthropometric and motor-test monitoring in young aerobic gymnasts. | No control; growth/maturation confounding; limited training-load and reliability reporting. |
| Laroëre et al. 2024 | Within-gymnast comparison of attentional-focus conditions during L-support execution. | Counterbalanced within-participant design supports inference, but sample is small and scorer blinding/reliability are unclear. |
| Mehrtash 2015 | Six-month one-group aerobic-gymnastics training responsiveness study in boys. | No control, growth/maturation confounding and limited blinding/reliability reporting. |
| Niu 2022 | 8-week VR aerobics training simulation-system evaluation in 24 athletes. | Sparse participant, allocation, intervention-dose, rater-reliability and statistical reporting. |
| Sang 2023 | 12-week functional-training intervention with FMS and special physical-fitness outcomes. | Very small sample, unclear allocation concealment, sparse intervention details and limited reliability/blinding reporting. |
| Sun 2022 | Core-strength training responsiveness in female college/competitive aerobics athletes. | Very small groups, unclear selection/allocation, sparse intervention reporting and limited reliability/blinding details. |
| Todorova 2021 | One-year annual-cycle training model in 12-14-year-old aerobic-gymnastics athletes. | Unclear allocation/concealment, no blinding, limited outcome-test reliability and maturation/co-intervention concerns. |
| Trisanda 2022 | Single-leg speed hop training with standing-broad-jump leg-power pre/post testing. | n=8, no control, incomplete intervention/adherence reporting and incomplete control of outside training. |
| Wu 2023 | Special lower-limb strength training with pre/post lower-extremity/core physical-fitness outcomes. | n=4, no control, unclear intervention dose/adherence, no blinding/reliability reporting and sparse/inconsistent reporting. |
| You 2023 | 12-week abdominal/core-strength intervention in 40 young competitive/aerobic-gymnastics athletes. | Sparse intervention details, unclear allocation implementation, no blinding/reliability reporting, unclear attrition and limited statistical transparency. |
| Zhang 2023 | 8-week suspension-training injury-control/recovery protocol in injured aerobic gymnasts. | Small sample, unclear allocation/concealment, no blinding/reliability reporting, short follow-up and several table/unit inconsistencies. |
| Artemieva 2019 | One-year special physical training methodology in 32 female 7-8-year-old sports-aerobics gymnasts. | Eligible training responsiveness but allocation, attrition, blinding and maturation confounding concerns. |
| Bota 2013 | HR-based routine functional-stress monitoring plus Ergosim neuromuscular-control pre-post evidence in 11 junior aerobic gymnasts. | No external control, small sample and limited statistics. |

**Supplementary Table S2E. JBI Checklist for Analytical Cross-Sectional Studies: final item-level judgments**

| **Item** | **Operational wording** |
| --- | --- |
| Q1 | Were inclusion criteria clearly defined? |
| Q2 | Were participants and setting described in detail? |
| Q3 | Was the exposure or monitoring variable measured validly and reliably? |
| Q4 | Were objective, standard criteria used for the condition or comparison? |
| Q5 | Were confounding factors identified? |
| Q6 | Were strategies to address confounding stated? |
| Q7 | Were outcomes measured validly and reliably? |
| Q8 | Was the statistical analysis appropriate? |

| **Study** | **Q1** | **Q2** | **Q3** | **Q4** | **Q5** | **Q6** | **Q7** | **Q8** |
| --- | --- | --- | --- | --- | --- | --- | --- | --- |
| Chayun 2024 | U | U | U | U | U | U | U | U |
| D’Anna et al. 2019 | U | U | U | U | U | U | U | U |
| Filippova 2006 | U | U | U | U | U | U | U | U |
| Ge 2025 | U | U | U | U | U | U | U | U |
| Guo 2026 | U | U | U | U | U | U | U | U |
| Kozina 2017 | U | U | U | U | U | U | U | U |
| Kyselovičová et al. 2023 | U | U | U | U | U | U | U | U |
| Panfilova 2025 | U | U | U | U | U | U | U | U |
| Puiu and Dragomir 2021 | U | U | U | U | U | U | U | U |
| Wang 2015 | U | U | U | U | U | U | U | U |
| Yu 2024 | U | U | U | U | U | U | U | U |
| Yusupava 2021 | U | U | U | U | U | U | U | U |
| Aleksandraviciene et al. 2015 | Y | Y | U | U | U | U | Y | U |
| Ayziaatullova et al. 2022 | U | U | U | U | U | N | U | U |

**Note.** Y, Yes; N, No; U, Unclear; NA, Not applicable. Intermediate or mixed archival ratings were conservatively standardized as U. JBI items were not summed and no global concern category was generated.

**Supplementary Table S2E-R. Principal rationale for each study**

| **Study** | **Evidence unit** | **Principal rationale** |
| --- | --- | --- |
| Chayun 2024 | Age-category psychophysiological monitoring profile in 38 female aerobic gymnasts. | Regional convenience sample and limited confounding/normative validation. |
| D’Anna et al. 2019 | Video/Kinovea kinematic comparison of pre-jump techniques. | n=4, pilot design, no measurement reliability and limited fatigue/order control. |
| Filippova 2006 | Morphofunctional/cardiorespiratory/fitness monitoring profile. | Standardized assessments/control comparison, but recruitment/confounding/reliability reporting limited. |
| Ge 2025 | Lower-limb biomechanical monitoring during rotational landings. | Objective instrumentation, but cross-sectional convenience sample and limited confounder control. |
| Guo 2026 | Aerobic-gymnastics subgroup from modified UFOV testing. | Eligible subgroup, but mixed context and limited confounding/generalizability. |
| Kozina 2017 | Multidomain readiness-monitoring profile. | Very small sample for factor/cluster analysis and limited reliability/confounding reporting. |
| Kyselovičová et al. 2023 | Aerobic-gymnastics subgroup from Y-Balance and Biodex testing. | Objective tools, but small subgroup and mixed-discipline context. |
| Panfilova 2025 | NS-Psychotest.Sport psychomotor monitoring in AG1 versus AG2 aerobic gymnasts. | Clear eligible sample and named tests, but small convenience sample and limited confounder control. |
| Puiu and Dragomir 2021 | Maximal vertical-jump protocol with OptoJump, HR and lactate in junior aerobic gymnasts. | Objective tools and clear protocol; small sample and limited validity/reliability evidence. |
| Wang 2015 | Fuzzy comprehensive body-function evaluation model for competitive aerobics athletes. | Poor sampling, eligibility, measurement-protocol, reliability, criterion-validity and external-validation reporting. |
| Yu 2024 | Vicon + sEMG biomechanical analysis of C715 in 16 aerobic gymnasts. | Strong instrumentation and task definition; small convenience sample, group-classification reliability and confounding control limited. |
| Yusupava 2021 | Complex preparedness test battery and factor structure in 20 young aerobic-gymnastics athletes. | Factor analysis with very small sample relative to indicators; limited test reliability, sampling detail and confounding control. |
| Aleksandraviciene et al. 2015 | All 15 female aerobic gymnasts; treadmill and competitive-routine physiological/energetic monitoring. | Objective physiological instruments but small sample and limited confounding control. |
| Ayziaatullova et al. 2022 | Anthropometric/body-segment assessment and L-sit performance in 9-11-year-old sports-aerobics gymnasts. | Limited sample, reliability and confounding reporting. |

**Supplementary Table S3A. Adapted COSMIN-informed framework and decision rules**

| **Property** | **COSMIN basis** | **Retained criteria** | **Modification for performance/physiological tests** | **Excluded or not applicable** | **Decision rule** |
| --- | --- | --- | --- | --- | --- |
| Reliability | COSMIN taxonomy and COSMIN Risk of Bias tool for reliability/measurement error | Stable construct between repeated measurements; appropriate interval; comparable test conditions; independent administration/scoring; suitable agreement statistic. | Also considered familiarization, warm-up, test order, equipment/software version and calibration, assessor instructions, preceding training/recovery, and environmental standardization. | Internal consistency and structural validity for single-score performance or physiological tests. | Criterion-level judgments: Met, Partly met/unclear, Not met, or Not applicable. No aggregate study score. ICC or weighted kappa was required for relative reliability; correlation alone was insufficient. |
| Measurement error | COSMIN taxonomy and COSMIN Risk of Bias tool for reliability/measurement error | SEM, SDC/MDC, limits of agreement, or justified coefficient of variation; repeated conditions comparable; error source compatible with intended use. | Interpretation considered biological day-to-day variation and technical variation arising from equipment, assessor, familiarization, recovery, and protocol standardization. | PROM-specific item-level error concepts. | Evidence was judged interpretable only when the error statistic and calculation were reported. Sufficiency relative to an MIC was not inferred when no MIC existed. |
| Content validity | COSMIN taxonomy; PROM-specific procedures not applied in full | Relevance, comprehensiveness, and comprehensibility of the task or test content for the intended construct and athlete population. | Expert review and athlete/task correspondence were considered; lack of athlete testing or stakeholder input was recorded explicitly. | PROM item-generation and cognitive-interview requirements when the instrument was not a questionnaire. | Formal content-validity evidence required an explicit development process; sport specificity alone was not treated as formal content validity. |
| Criterion validity | COSMIN taxonomy | Comparator had to be an acceptable reference standard for the same construct; analysis had to be appropriate. | Generic laboratory tests were not automatically treated as gold standards for sport-specific performance constructs. | PROM-specific reference standards. | Associations with a related but non-equivalent test were classified as construct validity rather than criterion validity. |
| Construct validity | COSMIN taxonomy and hypotheses-testing principles | Expected direction and magnitude of associations or group differences; comparator measured a related construct; appropriate analysis. | Known-groups evidence, convergent associations, and task-specific physiological/biomechanical relationships were eligible. | Structural validity for non-scale instruments. | Without a priori hypotheses, evidence was described as exploratory and not rated as confirmatory construct validity. |
| Responsiveness | COSMIN taxonomy; responsiveness treated as longitudinal validity | Explicit change-score question; expected direction/magnitude or external anchor; appropriate longitudinal analysis. | Training-cycle or intervention change was considered only when the study addressed the measurement method's ability to detect change rather than merely intervention efficacy. | Statistically significant pre-post change alone. | Observed change after training was recorded separately from formal responsiveness; significance alone did not establish responsiveness. |

**Note.** The adapted framework is not a standard full COSMIN rating. Each property was considered separately and no aggregate study score was calculated.

**Supplementary Table S3B. Property-specific appraisal of measurement-property studies**

| **Study** | **Instrument/evidence unit** | **Property** | **Evidence conclusion** | **Rationale** | **Principal limitations** |
| --- | --- | --- | --- | --- | --- |
| Alves 2015 | SAGAT/Borelli test: sport-specific anaerobic field test for elite female aerobic gymnasts. | Content validity | Not formally evaluated | Sport-specific task content was described, but no formal content-validity study was extracted. | Sport-specific test with validity/reliability/responsiveness evidence; small subsamples. |
| Alves 2015 | SAGAT/Borelli test: sport-specific anaerobic field test for elite female aerobic gymnasts. | Construct validity | Partly supported / exploratory | Associations with Wingate-derived indices and simulated competition support convergent validity; a priori hypotheses and equivalence to a reference standard were not established. | Sport-specific test with validity/reliability/responsiveness evidence; small subsamples. |
| Alves 2015 | SAGAT/Borelli test: sport-specific anaerobic field test for elite female aerobic gymnasts. | Reliability | Supported with limitations | Test-retest reliability was reported; small subsamples and incomplete detail on all repeat-test conditions limit certainty. | Sport-specific test with validity/reliability/responsiveness evidence; small subsamples. |
| Alves 2015 | SAGAT/Borelli test: sport-specific anaerobic field test for elite female aerobic gymnasts. | Measurement error | Partly supported | A minimal detectable change was reported; interpretation against an athlete-important change threshold was unavailable. | Sport-specific test with validity/reliability/responsiveness evidence; small subsamples. |
| Alves 2015 | SAGAT/Borelli test: sport-specific anaerobic field test for elite female aerobic gymnasts. | Responsiveness | Partly supported / exploratory | Change across training phases was reported, but formal responsiveness hypotheses or an external change anchor were not available. | Sport-specific test with validity/reliability/responsiveness evidence; small subsamples. |
| Budiarti 2022 | Aerobic-gymnastics flexibility test for National Development category athletes. | Content validity | Partly supported | Expert content-validity evidence was reported; direct athlete testing and stakeholder comprehensibility evidence were limited. | No empirical test-retest/criterion validity/responsiveness in athlete sample. |
| Budiarti 2022 | Aerobic-gymnastics flexibility test for National Development category athletes. | Reliability | Partly supported | Rater-based reliability was reported, but empirical test-retest reliability in the intended athlete population was not established. | No empirical test-retest/criterion validity/responsiveness in athlete sample. |
| Budiarti 2022 | Aerobic-gymnastics flexibility test for National Development category athletes. | Measurement error | Not demonstrated | No interpretable SEM, SDC/MDC, or limits-of-agreement estimate was extracted. | No empirical test-retest/criterion validity/responsiveness in athlete sample. |
| Budiarti 2022 | Aerobic-gymnastics flexibility test for National Development category athletes. | Criterion validity | Not evaluated | No acceptable reference standard was used. | No empirical test-retest/criterion validity/responsiveness in athlete sample. |
| Budiarti 2022 | Aerobic-gymnastics flexibility test for National Development category athletes. | Responsiveness | Not evaluated | No longitudinal responsiveness assessment was reported. | No empirical test-retest/criterion validity/responsiveness in athlete sample. |
| Lopes 2024 | Body-composition method comparison in six Brazilian female aerobic gymnasts. | Criterion validity | Not demonstrated | Methods were compared without a criterion reference such as DXA. | Very small sample, no criterion method and limited reliability/error evidence. |
| Lopes 2024 | Body-composition method comparison in six Brazilian female aerobic gymnasts. | Construct validity | Exploratory | Method-dependent differences and agreement between selected methods were reported. | Very small sample, no criterion method and limited reliability/error evidence. |
| Lopes 2024 | Body-composition method comparison in six Brazilian female aerobic gymnasts. | Reliability | Not evaluated | No test-retest reliability estimate was reported. | Very small sample, no criterion method and limited reliability/error evidence. |
| Lopes 2024 | Body-composition method comparison in six Brazilian female aerobic gymnasts. | Measurement error | Partly supported with major limitations | Bland-Altman analysis was reported, but the sample was six athletes and no interpretable change threshold was available. | Very small sample, no criterion method and limited reliability/error evidence. |
| Lopes 2024 | Body-composition method comparison in six Brazilian female aerobic gymnasts. | Responsiveness | Not evaluated | The 30-day comparison did not constitute a formal responsiveness study. | Very small sample, no criterion method and limited reliability/error evidence. |
| Yudho 2024 | Wireless inclinometer postural-stability monitoring in active and retired aerobic-gymnastics athletes. | Construct validity | Exploratory / insufficient | Known-groups comparisons and sensor-derived stability measures were reported in eight participants. | Known-groups/sensor evidence but no criterion validity, test-retest reliability or measurement error. |
| Yudho 2024 | Wireless inclinometer postural-stability monitoring in active and retired aerobic-gymnastics athletes. | Criterion validity | Not evaluated | No force-platform or motion-capture reference standard was used. | Known-groups/sensor evidence but no criterion validity, test-retest reliability or measurement error. |
| Yudho 2024 | Wireless inclinometer postural-stability monitoring in active and retired aerobic-gymnastics athletes. | Reliability | Not evaluated | No test-retest or inter-device reliability estimate was reported. | Known-groups/sensor evidence but no criterion validity, test-retest reliability or measurement error. |
| Yudho 2024 | Wireless inclinometer postural-stability monitoring in active and retired aerobic-gymnastics athletes. | Measurement error | Not evaluated | No SEM, SDC/MDC, coefficient of variation, or limits of agreement were reported. | Known-groups/sensor evidence but no criterion validity, test-retest reliability or measurement error. |
| Yudho 2024 | Wireless inclinometer postural-stability monitoring in active and retired aerobic-gymnastics athletes. | Responsiveness | Not evaluated | No longitudinal change assessment was reported. | Known-groups/sensor evidence but no criterion validity, test-retest reliability or measurement error. |

**Supplementary Table S4A. PROBAST signalling-question key**

| **Item** | **Operational wording** |
| --- | --- |
| 1.1 | Appropriate data source used |
| 1.2 | Participant inclusions and exclusions appropriate |
| 2.1 | Predictors defined and assessed similarly for all participants |
| 2.2 | Predictors assessed without knowledge of outcome |
| 2.3 | Predictors available when the model would be used |
| 3.1 | Outcome determined appropriately |
| 3.2 | Prespecified or standard outcome definition used |
| 3.3 | Predictors excluded from the outcome definition |
| 3.4 | Outcome defined and determined similarly for all participants |
| 3.5 | Outcome determined without knowledge of predictors |
| 3.6 | Time interval between predictor and outcome appropriate |
| 4.1 | Reasonable number of participants with the outcome |
| 4.2 | Continuous and categorical predictors handled appropriately |
| 4.3 | All enrolled participants included in analysis |
| 4.4 | Missing data handled appropriately |
| 4.5 | Predictor selection did not rely on univariable analysis |
| 4.6 | Data complexities handled appropriately |
| 4.7 | Relevant model-performance measures evaluated appropriately |
| 4.8 | Overfitting and optimism addressed |
| 4.9 | Final predictor weights corresponded to multivariable analysis |

**Note.** Responses: Y, Yes; PY, Probably yes; PN, Probably no; N, No; NI, No information. All four studies were model-development or internal-evaluation reports; no independent external validation study was identified.

**Supplementary Table S4B1. PROBAST item-level judgments**

| **Study** | **1.1** | **1.2** | **2.1** | **2.2** | **2.3** | **3.1** | **3.2** | **3.3** | **3.4** | **3.5** | **3.6** |
| --- | --- | --- | --- | --- | --- | --- | --- | --- | --- | --- | --- |
| Chen 2025 | PY | NI | PY | NI | Y | PY | PY | Y | Y | NI | Y |
| Li 2025 | NI | NI | NI | NI | PY | NI | NI | NI | NI | NI | NI |
| Luo et al. 2025 | PY | NI | PY | NI | Y | PY | NI | Y | PY | NI | Y |
| Zhao 2025 | PY | PY | Y | NI | Y | PY | PY | Y | Y | NI | Y |

**Note.** Y, Yes; PY, Probably yes; PN, Probably no; N, No; NI, No information. Item wording is given in Table S4A.

**Supplementary Table S4B2. PROBAST item-level judgments**

| **Study** | **4.1** | **4.2** | **4.3** | **4.4** | **4.5** | **4.6** | **4.7** | **4.8** | **4.9** |
| --- | --- | --- | --- | --- | --- | --- | --- | --- | --- |
| Chen 2025 | PN | NI | NI | NI | PY | PY | PN | PN | NI |
| Li 2025 | PN | NI | NI | NI | NI | NI | PN | PN | NI |
| Luo et al. 2025 | PN | PY | Y | NI | PY | Y | PY | PN | NI |
| Zhao 2025 | N | PY | PY | NI | PY | PN | Y | PN | PY |

**Note.** Y, Yes; PY, Probably yes; PN, Probably no; N, No; NI, No information. Item wording is given in Table S4A.

**Supplementary Table S4C. PROBAST domain-level, overall, and applicability judgments**

| **Study** | **Participants** | **Predictors** | **Outcome** | **Analysis** | **Overall RoB** | **Applicability** | **Principal rationale** |
| --- | --- | --- | --- | --- | --- | --- | --- |
| Chen 2025 | Unclear | Unclear | Unclear | High | High | Unclear | Eligible sensor prediction evidence, but internal validation only and small validation sample. |
| Li 2025 | Unclear | Unclear | Unclear | High | High | Unclear | Eligible decision-support evidence with very limited certainty. |
| Luo et al. 2025 | Unclear | Unclear | Unclear | High | High | Unclear | Classifier eligible but limited by annotation and internal validation. |
| Zhao 2025 | High | Unclear | Unclear | High | High | Unclear | Strong sport-specific instrumentation, but sample size, internal validation and segment/athlete label structure drive high RoB. |

**Supplementary Table S5A. RoB 2 signalling-question key**

| **Item** | **Operational wording** |
| --- | --- |
| 1.1 | Allocation sequence random |
| 1.2 | Allocation sequence concealed until enrolment and assignment |
| 1.3 | Baseline differences suggest a problem with randomization |
| 2.1 | Participants aware of assigned intervention |
| 2.2 | Carers or intervention deliverers aware of assigned intervention |
| 2.3 | Deviations from intended intervention arose because of trial context |
| 2.4 | Such deviations were likely to affect the outcome |
| 2.5 | Such deviations were balanced between groups |
| 2.6 | Analysis appropriate for the effect of assignment |
| 2.7 | Failure to analyse in assigned groups could materially affect the result |
| 3.1 | Outcome data available for all or nearly all randomized participants |
| 3.2 | Evidence that the result was not biased by missing outcome data |
| 3.3 | Missingness could depend on the true outcome value |
| 3.4 | Missingness was likely to depend on the true outcome value |
| 4.1 | Outcome measurement method inappropriate |
| 4.2 | Outcome measurement or ascertainment could differ between groups |
| 4.3 | Outcome assessors aware of intervention received |
| 4.4 | Outcome assessment could be influenced by knowledge of intervention |
| 4.5 | Outcome assessment was likely influenced |
| 5.1 | Analysis followed a prespecified plan finalized before unblinded data |
| 5.2 | Result likely selected from multiple eligible outcome measurements |
| 5.3 | Result likely selected from multiple eligible analyses |

**Note.** Responses follow the RoB 2 signalling-question format. The assessment targets the effect of assignment to intervention.

**Supplementary Table S5B1. RoB 2 signalling-question judgments**

| **Study** | **1.1** | **1.2** | **1.3** |
| --- | --- | --- | --- |
| Luo et al. 2025 | PY | NI | PN |
| Ma et al. 2024 (JSSM) | Y | NI | PN |
| Ma et al. 2025 | Y | Y | PN |

**Note.** See Table S5A for item wording. Domain judgments are reported in Table S5C; signalling-question responses should be interpreted using the RoB 2 algorithms rather than by simple counting.

**Supplementary Table S5B2. RoB 2 signalling-question judgments**

| **Study** | **2.1** | **2.2** | **2.3** | **2.4** | **2.5** | **2.6** | **2.7** |
| --- | --- | --- | --- | --- | --- | --- | --- |
| Luo et al. 2025 | Y | Y | NI | NI | NI | PY | NI |
| Ma et al. 2024 (JSSM) | Y | Y | NI | NI | NI | Y | PY |
| Ma et al. 2025 | Y | Y | NI | NI | NI | PY | PN |

**Note.** See Table S5A for item wording. Domain judgments are reported in Table S5C; signalling-question responses should be interpreted using the RoB 2 algorithms rather than by simple counting.

**Supplementary Table S5B3. RoB 2 signalling-question judgments**

| **Study** | **3.1** | **3.2** | **3.3** | **3.4** |
| --- | --- | --- | --- | --- |
| Luo et al. 2025 | PY | PY | PN | NI |
| Ma et al. 2024 (JSSM) | PY | PY | PN | NI |
| Ma et al. 2025 | Y | NI | PN | NI |

**Note.** See Table S5A for item wording. Domain judgments are reported in Table S5C; signalling-question responses should be interpreted using the RoB 2 algorithms rather than by simple counting.

**Supplementary Table S5B4. RoB 2 signalling-question judgments**

| **Study** | **4.1** | **4.2** | **4.3** | **4.4** | **4.5** |
| --- | --- | --- | --- | --- | --- |
| Luo et al. 2025 | N | PN | NI | PN | NI |
| Ma et al. 2024 (JSSM) | N | N | N | N | N |
| Ma et al. 2025 | N | N | N | N | N |

**Note.** See Table S5A for item wording. Domain judgments are reported in Table S5C; signalling-question responses should be interpreted using the RoB 2 algorithms rather than by simple counting.

**Supplementary Table S5B5. RoB 2 signalling-question judgments**

| **Study** | **5.1** | **5.2** | **5.3** |
| --- | --- | --- | --- |
| Luo et al. 2025 | NI | NI | NI |
| Ma et al. 2024 (JSSM) | NI | NI | NI |
| Ma et al. 2025 | NI | NI | NI |

**Note.** See Table S5A for item wording. Domain judgments are reported in Table S5C; signalling-question responses should be interpreted using the RoB 2 algorithms rather than by simple counting.

**Supplementary Table S5C. RoB 2 domain-level and overall judgments**

| **Study** | **D1 Randomization** | **D2 Deviations** | **D3 Missing data** | **D4 Outcome measurement** | **D5 Selective reporting** | **Overall** | **Principal rationale** |
| --- | --- | --- | --- | --- | --- | --- | --- |
| Luo et al. 2025 | Some concerns | Some concerns | Some concerns | Some concerns | Some concerns | Some concerns | Randomization/adherence/blinding/prespecification reporting incomplete. |
| Ma et al. 2024 (JSSM) | Some concerns | Some concerns | Some concerns | Low risk | Some concerns | Some concerns | Randomized design and blinded evaluators, but no registration/protocol and incomplete adherence/co-intervention reporting. |
| Ma et al. 2025 | Low risk | Some concerns | Some concerns | Low risk | Some concerns | Some concerns | Random sequence and opaque-envelope allocation were reported; three participants did not complete final assessment, no intention-to-treat analysis or prespecified protocol was available, and some outcome values were presented graphically. Independent blinded assessors and objective performance tests reduced outcome-measurement bias. |
